# Adaptively evolved *Methylorubrum extorquens* with enhanced formate tolerance and its application in 3-hydroxypropionic acid production

**DOI:** 10.1128/aem.02560-24

**Published:** 2025-08-13

**Authors:** Xuhua Mo, Yan Zhao, Lin Zhu, Changtai Zhang, Zhe Liu, Zengxin Ma, Kai Bao, Song Yang

**Affiliations:** 1 School of Life Sciences, Shandong Province Key Laboratory of Applied Mycology, and Qingdao International Center on Microbes Utilizing Biogas, Qingdao Agricultural University 98431, Qingdao, Shandong, People’s Republic of China; 2 School of Life Sciences, Hubei University 12563, Wuhan, Hubei, People’s Republic of China; 3 Key Laboratory of Systems Bioengineering, Ministry of Education, Tianjin University 643578 , Tianjin, People’s Republic of China; University of Nebraska-Lincoln, Lincoln, Nebraska, USA

**Keywords:** *Methylorubrum extorquens*, formate-utilizing bacterium, adaptive laboratory evolution, formate oxidation and assimilation pathway, 3-hydroxypropionic acid production

## Abstract

*Methylorubrum extorquens* AM1, a native formate-utilizing bacterium, has exhibited limited capacity to tolerate formate. In this study, we employed an adaptive laboratory evolution (ALE) strategy to develop an evolved strain FT3 derived from *M. extorquens* AM1, with enhanced formate tolerance. When cultivated with a mixture of carbon sources containing 90 mM formate and 30 mM methanol, the FT3 strain exhibited 5.3 times higher optical density (OD_600_) compared to the parental strain. FT3 strain was shown to efficiently utilize both methanol and formate in experiments using ^13^C-labeled carbon sources. Furthermore, the mechanism underlying the enhanced formate tolerance in FT3 strain was investigated through a combination of DNA re-sequencing, transcriptome analysis, and ALE-inspired gene manipulation experiments. The FT3 strain was identified as a hypermutant, and its enhanced formate tolerance was attributed to increased formate transport, an improved methanol oxidation pathway, and enhanced formate oxidation and assimilation pathways. In addition, gene overexpression experiments indicated the involvement of genes META1_0287*, *META1_3027*, *META1_3028*, *META1_3029*, *META1_1261*, *META1_1418*, and *META1_2965* in formate tolerance. Notably, the addition of formate resulted in a significant improvement in the generation of NADH and NADPH in the FT3 strain. Moreover, using the FT3 strain as a chassis, an improved 3-hydroxypropionic acid (3-HP) production of 2.47 g/L through fed-batch fermentation was achieved. This study provides an important foundation for further engineering of the evolved *M. extorquens* strain as an efficient platform for the co-utilization of methanol and formate in the production of reduced chemicals.

**IMPORTANCE:**

In the present study, we successfully obtained an evolved strain FT3 derived from M. extorquens AM1 with high formate tolerance using the ALE strategy. The FT3 strain was identified as a hypermutant, with its enhanced formate tolerance attributed to increased formate transport, an improved methanol oxidation pathway, and enhanced formate oxidation and assimilation pathways. Through transcriptome analysis and ALE-inspired gene manipulation experiments, we identified several genes that contribute to the FT3 strain’s tolerance to formate. The enhanced levels of reducing equivalents and the increased tolerance to 3-HP make FT3 a suitable chassis for 3-HP production, achieving an improved yield of 2.47 g/L through fed-batch fermentation. This study provides an important foundation for further engineering of the evolved *M. extorquens* strain as an efficient platform for the co-utilization of methanol and formate in the production of reduced chemicals.

## INTRODUCTION

Currently, fossil fuels and sugar-based raw materials are still the main sources used to produce value-added chemicals, which not only compete with human consumption but also threaten food security (1, 2). In addition, the extensive use of fossils has led to energy shortages and the emission of greenhouse gases such as carbon dioxide (CO_2_), which has spurred global concerns. Therefore, there is an urgency to develop green and sustainable approaches to access chemical production, rather than relying on fossil fuels and sugars (1, 2). Among the methods, the electrochemical reduction of CO_2_ to formate is a promising avenue of research (3–6). Formate has become a mediator between the physico-chemical and biological realms, as it can serve as the sole carbon and energy source for microbial growth (2). Furthermore, formate has emerged as an alternative feedstock for microbial fermentation due to its relatively low cost, high abundance, and high solubility (3, 4, 7).

Native formatotrophic microbes employ two different strategies to grow on formate. In the first strategy, the reducing equivalents generated from the oxidation of formate support carbon fixation through the Calvin-Benson-Bassham cycle and promote cell growth, which has been identified in *Cupriavidus necator* and *Paracoccus denitrificans* (8–10). The second strategy involves the direct condensation of formate with tetrahydrofolate (THF), catalyzed by formate-tetrahydrofolate ligase. This leads to the production of the intermediate formyl-THF, which serves as a precursor for several pathways, including the reductive acetyl-CoA pathway (also known as the Wood-Ljungdahl pathway), the serine cycle, and the reductive glycine pathway (10–15). The application of native formate-utilizing microbes is currently constrained by a number of factors, including their slow growth and low titer and yield of the chemical production (7, 16–18). To address these issues and render formate a more feasible carbon source in biorefinery applications, the adaptive laboratory evolution (ALE) has been conducted with native formate-utilizing microbes, such as *Thermococcus onnurineus* and *C. necator* (19, 20). This approach has resulted in a notable enhancement in the tolerance and utilization of formate by the evolved microbes. More recently, the formate assimilation pathways have been introduced into traditional industrial microorganisms, such as *Escherichia coli* and yeast, thereby creating the synthetic formatotrophic microbes (21–26). The combination of ALE technology with synthetic formatotrophic microbes has demonstrated the enhanced formate tolerance and utilization (21, 23, 25, 26). However, the growth of synthetic microbes generally requires the utilization of other carbon sources, with a notable reduction in efficiency compared to traditional carbon sources (21–26).

*Methylorubrum extorquens* AM1 (also known as *Methylobacterium extorquens* AM1) is a representative of methylotrophs capable of utilizing one-carbon compounds (such as methanol and formate) as their carbon and energy source (13). Formate, the oxidative product of methanol, is an important branch point intermediate in methylotrophic metabolism as it can either be oxidized to CO_2_ to generate reducing equivalents or be condensed with THF to be assimilated through the serine cycle (27, 28). It has been observed that the addition of formate as a supplemental source significantly increases the reducing equivalents and enhances the production of chemicals such as mevalonate, poly-3-hydroxybutyrate, and polyhydroxyalkanoates in *M. extorquens* AM1 or its derivative strains (28–31). Nevertheless, despite being a native formatotroph, it has been demonstrated that a severe reduction in growth occurs when the formate concentration is increased to more than 20 mM (Fig. S1), which is significantly lower than the standard cultivation concentration of methanol (i.e., 120 mM). Such a low tolerance to formate restricts the potential for the use of high concentrations of formate in the bioprocess of *M. extorquens* AM1.

In this study, we employed the ALE method to obtain an evolved *M. extorquens* strain that exhibited tolerance to and assimilation of high concentrations of formate. Subsequently, we investigated the underlying mechanisms of formate tolerance through a combination of DNA re-sequencing, transcriptome analysis, and ALE-inspired gene manipulation experiments. Moreover, in comparison to the native *M. extorquens* AM1 host chassis, the production of 3-hydroxypropionic acid (3-HP) was significantly increased in the evolved strain using the combined methanol and formate carbon sources. The present study highlighted the potential of the evolved *M. extorquens* strain with enhanced formate tolerance in the production of reduced chemicals.

## MATERIALS AND METHODS

### Strains, media, and culture conditions

The plasmids and strains used and generated in this study are listed in Table 1. All *E. coli* strains were grown on Luria-Bertani (LB) agar or liquid medium with appropriate antibiotics at 37°C. The final concentrations of antibiotics used in this study were 20 µg/mL tetracycline (Tet), 25 µg/mL kanamycin (Km), and 80 µg/ml apramycin (Apr). Unless otherwise stated, all chemicals used in the culture medium were purchased from Sigma-Aldrich (St. Louis, MO, USA).

**TABLE 1:** Plasmids and strains used in this study

Plasmid or strain	Description	Source
Plasmids		
pCM80	Vector used for gene expression in *M. extorquens* AM1; promoter, *P_mxaF_*; antibiotics, Tet^R^	(32)
pCM80-Apr	Vector used for gene expression in *M. extorquens* FT3; promoter, *P_mxaF_*; antibiotics, Apr^R^	This study
pCM80-Apr-mcr	pCM80-Apr containing the operon *P_mxaF_*::*mcr-P_meta1_*__3616_::*mcr*_550–1219_ from pYM07 for the synthesis of 3-HP	This study
pCM130	Promoter probe vector with XylE as reporter	(32)
pCM130-P_mxaF*_	Plasmid carrying the mutated promoter P_mxaF*_ to drive *xylE*	This study
pCM130-*P_mxaF_*	Plasmid carrying the promoter *P_mxaF_* to drive *xylE*	This study
pCM130-P_fdh2*_	Plasmid carrying the mutated promoter P_fdh2*_ to drive *xylE*	This study
pCM130-*P_fdh2_*	Plasmid carrying the promoter *P_fdh2_* to drive *xylE*	This study
pYM07	pCM80 derivative strain harboring P*_mxaF_*::*mcr-P_meta1_3616_*::*mcr_550_*_–_*_1219_*	(33)
pCM80-0287	Plasmid overexpressing the gene *META1_0287*	This study
pCM80-0287*	Plasmid overexpressing the mutated gene META1_0287*	This study
pCM80-2965	Plasmid overexpressing the gene *META1_2965*	This study
pCM80-3029	Plasmid overexpressing the gene *META1_3029*	This study
pCM80-1261	Plasmid overexpressing the gene *META1_1261*	This study
pCM80-1418	Plasmid overexpressing the gene *META1_1418*	This study
pCM80-3027	Plasmid overexpressing the gene *META1_3027*	This study
pCM80-3028	Plasmid overexpressing the gene *META1_3028*	This study
pCM80-1261-1260	Plasmid overexpressing the genes *META1_1261* and *META1_1260*	This study
pCM80-3028-3027	Plasmid overexpressing the genes *META1_3028* and *META1_3027*	This study
pCM80-3028-3027-3029	Plasmid overexpressing the genes *META1_3028*, *META1_3027,* and *META1_3029*	This study
pCM433-MxaF*	Plasmid for homologous exchange of *P_mxaF_* promoter of MxaF with P_mxaF_*	This study
pCM433-*P_mxaF_-P_3028-3027_*	Plasmid for homologous exchange of a native promoter of the operon *META1_*3028-*META1_*3027 with *P_mxaF_* promoter	This study
Strains		
*M. extorquens* AM1	Wild-type strain	(9)
*M. extorquens* AM1Δ*celAB*	The gene *celAB* was knocked out in *M. extorquens* AM1	This study
FT1 to FT12	Adaptively evolved strains of WTKC with formate tolerance	This study
*M. extorquens* AM1Δ*celAB-*Pro	*M. extorquens* AM1Δ*celAB* carrying the plasmid pYM07	This study
FT3::pCM80-Apr-mcr	FT3 carrying the plasmid pCM80-Apr-mcr	This study
AM1::pCM130-P_mxaF*_	*M. extorquens* AM1 carrying the plasmid pCM130-P_mxaF*_	This study
AM1::pCM130-*P_mxaF_*	*M. extorquens* AM1 carrying the plasmid pCM130-*P_mxaF_*	This study
AM1-MxaF*	The promoter of *P_mxaF_* in AM1 is replaced by the mutated P_mxaF__*_	This study
AM1::pCM130-P_fdh2*_	*M. extorquens* AM1 carrying the plasmid pCM130-P_fdh2*_	This study
AM1::pCM130-*P_fdh2_*	*M. extorquens* AM1 carrying the plasmid pCM130-*P_fdh2_*	This study
AM1::pCM80-0287	*M. extorquens* AM1 carrying the plasmid pCM80-0287	This study
AM1::pCM80-0287*	*M. extorquens* AM1 carrying the plasmid pCM80-0287*	This study
AM1::pCM80-2965	*M. extorquens* AM1 carrying the plasmid pCM80-2965	This study
AM1::pCM80-3029	*M. extorquens* AM1 carrying the plasmid pCM80-3029	This study
AM1::pCM80-1261	*M. extorquens* AM1 carrying the plasmid pCM80-1261	This study
AM1::pCM80-1418	*M. extorquens* AM1 carrying the plasmid pCM80-1418	This study
AM1::pCM80-3027	*M. extorquens* AM1 carrying the plasmid pCM80-3027	This study
AM1::pCM80-3028	*M. extorquens* AM1 carrying the plasmid pCM80-3028	This study
AM1::pCM80-1261-1260	*M. extorquens* AM1 carrying the plasmid pCM80-1261-1260	This study
AM1::pCM80-3028-3027	*M. extorquens* AM1 carrying the plasmid pCM80-3028-3027	This study
AM1::pCM80-3028-3027-3029	*M. extorquens* AM1 carrying the plasmid pCM80-3028-3027-3029	This study
AM1-*P_maxF_*-3028-3027	The operon of *META1_3028* and META1_3027*M* in *M. extorquens* AM1 is driven by promoter *P_maxF_*	This study
*E. coli* DH5α	Gene cloning in host bacteria	Lab storage
*E. coli* Top10	Gene cloning in host bacteria	Lab storage

### Analysis of growth and biomass

*M. extorquens* AM1 and its derivative strains were first cultivated as seed cultures in test tubes according to previously described methods. The seed cultures were then transferred to 250 mL flasks containing 50 mL of Hypho minimal medium with different carbon sources (34). Unless otherwise noted, 120 mM methanol or 150 mM methanol was used as the sole carbon source. For cultivating the *M. extorquens* FT3 with different carbon sources, 34 mM ethanol, 5 mM acetate, 68 mM 1,2-propanediol, or 36 mM pyruvate was used as the sole carbon source. To investigate the tolerance of the FT3 strain to 3-HP or formaldehyde, the *M. extorquens* FT3 strains were first cultivated with 120 mM methanol to an optical density at 600 nm (OD_600_) of 0.8. Then, 3-HP was added at final concentrations of 100 mg/L, 200 mg/L, 500 mg/L, and 1,000 mg/L, while formaldehyde was added at final concentrations of 2.5 mM, 5 mM, 7.5 mM, 10 mM, and 12.5 mM. For investigating the derivative strains with overexpressing genes, these strains were cultivated with 120 mM methanol and 10 mM, 15 mM, or 20 mM formate, respectively.

All the tubes or flasks were incubated on a rotary shaker at 200 rpm and 30°C with an initial OD_600_ of approximately 0.02. A 0.5 mL sample was taken at each time point for OD_600_ measurement using a UV-visible spectrophotometer (Genesys10S, CA, USA). The specific growth rates were calculated by fitting an exponential growth model using Curve Fitter software (35). The presented specific growth rates represent the mean plus standard deviations calculated from triplicate biological replicates.

### ALE of *M. extorquens* AM1

The ALE of the *M. extorquens* AM1 strain (*celAB* deleted) was conducted using Hypho medium at 30°C. One colony of *M. extorquens* AM1*ΔcelAB* strain was initially inoculated into the flask for cultivation on medium with 90 mM methanol and 30 mM sodium formate, with four parallel cultures prepared. ALE was then conducted through serial transfer every 48 hours. After 10 passages of ALE (approximately 10th generation), the maximum biomass of the AM1 strain gradually increased from an OD_600_ of 0.2 to 1.2. The microbial dilutions were then plated on solid media containing methanol and sodium formate as the carbon sources. Three individual colonies were selected from each plate, resulting in a total of 12 colonies for further subculture. During the subculture process, the methanol concentration in the medium was gradually decreased from 90 mM to 30 mM, while the concentration of sodium formate was increased from 30 mM to 90 mM. For each adjustment, 5 mM of sodium formate was added, and 5 mM of methanol was removed. This adjustment was continued until the OD_600_ value of each subculture became stable. At the 30th, 90th, 150th, 250th, and 300th generation (corresponding to 15th, 25th, 35th, 55th, and 70th passages, respectively), the maximum OD_600_ and formate uptake rates were measured, respectively. After 300 generations, the 12 evolved lineages showed significant growth on medium containing 90 mM sodium formate and 30 mM methanol. Then the strains from the 12 evolved lineages were plated on solid media containing 90 mM sodium formate and 30 mM methanol. Based on morphology, one large colony was selected for each evolved lineage and further evaluated for specific growth rate under cultivation with either 90 mM sodium formate and 30 mM methanol or 120 mM formate.

### Measurement of methanol, formate, and 3-HP concentrations in the culture medium

To determine the concentrations of methanol, sodium formate, and 3-HP in the culture medium or fermentation broth, a subculture of 800 µL was harvested and centrifuged at 13,000 rpm for 10 min at 4°C. The resulting supernatant was then filtered using a 0.22 µm membrane and subjected to high-performance liquid chromatography (HPLC) analysis as described previously (36). The HPLC analysis was performed on Wooking K2025 (China) equipped with a PDA (K2025DAD, Wooking Corporation, China) and a refractive index detector (RID-20A, Shimadzu Corporation, Japan). The analytical procedures were as follows: the mobile phase sample volume was 30 µL, with a flow rate of 0.6 mL/min. The column temperature was maintained at 65°C and the detector temperature at 35°C.

### Measurement of carotenoids from the evolved strain *M. extorquens* FT3

*M. extorquens* FT3 was cultivated in Hypho medium containing either 120 mM methanol, 90 mM methanol and 30 mM formate, or 60 mM methanol and 60 mM formate. Three replicates were prepared for each culture condition. After a cultivation period of 4 days, the broths were centrifuged at 8,000 × *g* for 3 min. The resulting pellets were then extracted and analyzed according to the method described previously (34).

### Determination of ^13^C-labeled amino acids in the *M. extorquens* FT3 strain

For the ^13^C labeling assay, the evolved strain FT3 was cultivated using two different carbon sources: 90 mM ^13^C-labeled sodium formate and 30 mM methanol, and 90 mM sodium formate and 30 mM ^13^C methanol. When the FT3 strain reached an OD_600_ of 0.6, the cells were collected from a 20 mL culture using a percolator with a 0.22 µm filter membrane. Protein extraction was performed as previously described with slight modifications (37, 38). The collected cells were rapidly frozen using liquid nitrogen and then dried in a lyophilizer for 12 hours. 20 mL of boiling water was then added and incubated in a water bath at 100°C for 10 min, vortexing three times for approximately 5 seconds each time. Next, the proteins were precipitated at 0°C for 20 minutes and centrifuged twice at 5,000 rpm for 20 min at 4°C. The supernatant was discarded, and the cell pellet was hydrolyzed by using 1 mL of 6 M HCl at 105°C for 24 hours. HCl was removed using a nitrogen blower, and the hydrolyzed samples were re-dissolved in 500 µL of ddH_2_O and filtered through a 0.22 µm membrane. For derivatization, 50 µL of solvent was evaporated and re-dissolved in 50 µL of 25 mg/mL methoxylamine hydrochloride in pyridine was added to the samples, which were incubated at 60°C for 30 minutes and vortexed occasionally. Then, 50 µL of *N*-methyl-*N*-trimethylsilyltrifluoroacetamide was added, and the samples were incubated at 30°C for an additional 90 minutes. After the derivatization reactions, the samples were centrifuged at 12,000 × *g* for 10 min, and the supernatant was subjected to GC-MS. The derivatized amino acid samples were analyzed by GC/Q-TOF-MS with an Agilent 5975B/6890N gas chromatography-mass spectrometer equipped with an HP-5MS (30 m × 0.25 mm×0.25 µm) chromatographic column. The carrier gas used was ultra-high-pressure pure helium, with a flow rate of 1 mL/min. The injection volume was set at 1 µL in the no-shunt mode. The injection port and transfer tube temperatures were kept at 280°C. The temperature gradient for the column was as follows: starting at 60°C for 0.25 minutes, with an increase of 5 °C/min until reaching 280°C, and holding this temperature for 10 minutes. Mass spectra of amino acids were in the mass range of 50–650 m/z at an acquisition rate of 5 spectra/s. The ion source temperature was set at 230°C. Finally, the resulting data from the chromatographic mass spectra were analyzed using GC-MS analytical software.

### Whole-genome sequencing of the *M. extorquens* FT3 strain

Genomic DNA was extracted from the evolved strain FT3 using the Wizard Genomic DNA Purification Kit (Promega (Beijing) Biotech Co., China). The genomic DNA sequencing was performed by Novogene (Tianjing, China) using the PacBio RS II platform. The process followed Novogene’s standardized protocols.

### Transcriptome analysis

The *M. extorquens* AM1 *ΔcelAB* strain was cultivated on medium with 150 mM methanol as the carbon source. The *M. extorquens* FT3 strain was cultivated with medium containing either 150 mM methanol or a combination of 120 mM methanol and 30 mM formate. Three parallels were prepared for each sample. Cells were collected during the middle exponential phase by centrifugation at 4°C for RNA isolation. RNA preparation, library construction, and RNA sequencing were conducted by Novogene (Tianjin, China). The quality of raw sequence reads was evaluated using the FASTQC software (v.0.10.1). Low-quality reads and bases from both ends of raw Illumina reads were removed and trimmed using the NGSQC Toolkit (v.2.3.3). BWA alignment software (v.0.7.17) was used to align the high-quality reads against the *M. extorquens* AM1 reference genome. SAM tools software (v.1.9) was used to sort and index the mapping results. Raw read counts from the resulting BAM files were obtained using HTSeq software (v.0.11.2). The raw-count table was further processed using the DESeq function of the DESeq2 package (v.1.18.1) to obtain gene expression data. Genes with a false discovery rate (FDR) *P*-value < 0.05 and log_2_ (fold change) >0.5 or < −0.5 were considered to be differentially expressed. Pearson’s linear correlation coefficients between variables were calculated using the R package “stats” and plotted using “corrplot.”

### Measurement of NADH and NADPH

The *M. extorquens* AM1 *ΔcelAB* strain was cultivated on medium with 120 mM methanol as the carbon source. The *M. extorquens* FT3 strain was cultivated with medium containing either 120 mM methanol or a combination of 90 mM methanol and 30 mM formate. Three parallels were prepared for each sample. When the strains reached an OD_600_ of approximately 0.6, 10 mL of cells were collected by centrifugation to measure the intracellular concentrations of NADH and NADPH. The collected samples were immediately quenched with liquid nitrogen and then dried using a freeze-dryer at −45°C. NADH and NADPH were extracted, and the concentrations were determined as previously described with minor revisions (39, 40). Briefly, 1 mL of buffer (300 mM KOH, 1% Triton X-100) was added to the samples, followed by incubation in a water bath at 85°C for 3 minutes with occasional shaking. The samples were then cooled on ice for an additional 3 minutes and centrifuged at 6,000 rpm for 10 minutes at 4°C. The supernatant was transferred to a new tube, and the pH was adjusted to 10 with potassium perchlorate. After centrifugation at 14,000 rpm for 15 minutes, the supernatant was subjected to HPLC analysis. The analyses of NADH and NADPH were performed on HPLC equipped with a NovaPac C18 RP column (3.9 × 150 mm, 60 Å, 4 µm, Waters) with elution gradient conditions as follows: 0–4 min, 100% mobile phase A; 4–18 min, 0%–30% mobile phase B; 18–23 min, 30%–100% mobile phase B; 23–25 min, 100% mobile phase B; 25–26 min, 100% mobile phase A; 26–50 min, 100% mobile phase A.

### Gene overexpression and gene mutation in *M. extorquens* AM1

The plasmid of pCM80 was used for gene overexpression in the *M. extorquens* strains. The targeted genes were amplified from the genomic DNA of the *M. extorquens* strains by PCR using the corresponding primers. The PCR products were inserted into the pCM80 plasmid under the control of the *P_mxaF_* promoter using the ClonExpress II One Step Cloning Kit (Vazyme Biotech, China). The resulting plasmids were introduced into the *M. extorquens* strains by electroporation. The *M. extorquens* AM1 *ΔcelAB* strain and SNP mutant strains were obtained from the *M. extorquens* strains using previously described methods (34). The genotypes of the *M. extorquens* derivatives were verified by PCR and DNA sequencing.

### Determining the activities of FDH

The 50 mL of the *M. extorquens* AM1 *ΔcelAB* and *M. extorquens* FT3 strains was, respectively, collected by centrifugation when the OD_600_ reached 0.8. The cells were washed twice, resuspended in 50 mM Tricine-KOH buffer (pH 7.0), and lysed using a French pressure cell at 1.2 × 10^8^ Pa. After centrifugation for 10 min at 12,000 *× g*, the supernatants were transferred to a new tube, and the final volume was adjusted to 7 mL with 50 mM Tricine-KOH buffer (pH 7.0). The protein concentration in the crude extract was determined using the BCA Protein Assay Kit (Sangon Biotech., China). The measurements were performed at room temperature. The reaction system containing 0.5 mM NAD^+^ and 1 mg/mL crude extract proteins was used to assay the FDH activity. The formate dehydrogenase activity was determined as described previously (41). Enzyme assays were performed in triplicate.

### Determination of the promoter strength using XylE-based experiments

The promoter strengths were determined using XylE-based experiments. The *P_mxaF_* and *P_fdh2_* promoters were amplified from the genomic DNA of the *M. extorquens* AM1 *ΔcelAB* strain, and the P_mxaF*_ and P_fdh2*_ promoters with SNP mutations were amplified from the genomic DNA of the *M. extorquens* FT3 strain. The PCR fragments were inserted into the pCM130 vector to control the expression of *xylE*. The resulting plasmids were separately introduced into *M. extorquens* AM1. These *M. extorquens* AM1 strains were cultivated on medium containing 120 mM methanol, and the cells were collected when the OD_600_ reached 0.8. The cells were lysed using the One-Shot Press (Constant Systems Cell Disruptor, Constant Systems, UK). The debris was discarded by centrifugation, and the supernatant was used to detect catechol dioxygenase activity as described previously (41). The reaction system, containing 5 mM catechol and 4 mg/mL crude extract proteins, was used for the assay of catechol dioxygenase activity. Enzyme assays were performed in triplicate.

### Fed-batch fermentation of *M. extorquens* FT3::pCM80-Apr-mcr

The 3-HP synthetic operon was amplified from the plasmid of pYM07 and then inserted into pCM80-Apr to generate the plasmid pCM80-Apr-mcr. The plasmid pCM80-Apr-mcr was further introduced into the *M. extorquens* FT3 strain to generate the *M. extorquens* FT3::pCM80-Apr-mcr strain. The strain *M. extorquens* FT3::pCM80-Apr-mcr was initially cultivated as a seed culture in a 500 mL flask. When the culture reached an OD_600_ of approximately 1.0, it was transferred to a 3 L fermenter containing 1.7 L of Choi3 medium (5,370 mg/L Na_2_HPO_4_·12H_2_O, 1305 mg/L KH_2_PO_4_, 450 mg/L MgSO_4_·7H_2_O, 250 mg/L (NH_4_)_2_SO_4_, 10 mg/L Na_2_EDTA, 1 mg/L FeSO_4_·7H_2_O, 1.4 mg/L CaCl_2_·2H_2_O, 1 mg/L MnCl_2_·4H_2_O, 0.2 mg/L Na_2_MoO_4_·2H_2_O, 0.3 mg/L CuSO_4_·5H_2_O, 3.2 mg/L CoCl_2_·6H_2_O, 4.4 mg/L ZnSO_4_·7H_2_O). The initial fermentation conditions included a temperature of 30℃, a stirring speed of 500 rpm, and a ventilation rate of 1 L/min. The aeration rate (ranging from 1 to 3 L/min) and the stirring speed (ranging from 500 to 800 rpm) were adjusted based on the dissolved oxygen level to maintain it above 20%. The initial concentration of methanol was 120 mM, while the concentration of sodium formate was 21.2 mM. Methanol concentration was monitored using a methanol electrode, and the concentration of formate was kept within the range of 8 to 12.7 mM. During the fermentation, the pH value was maintained at 6.9 by adding 1M sodium hydroxide for about 40 hours. The nitrogen concentration was maintained at 1 to 2 g/L by adding ammonium sulfate. After 40 hours, measurements were taken every 6 hours to determine the OD_600_, methanol and formate consumption, dry cell weight, and 3-HP titer.

## RESULTS

### Generating the evolved strains with enhanced tolerance to formate using ALE

**Fig 1:**
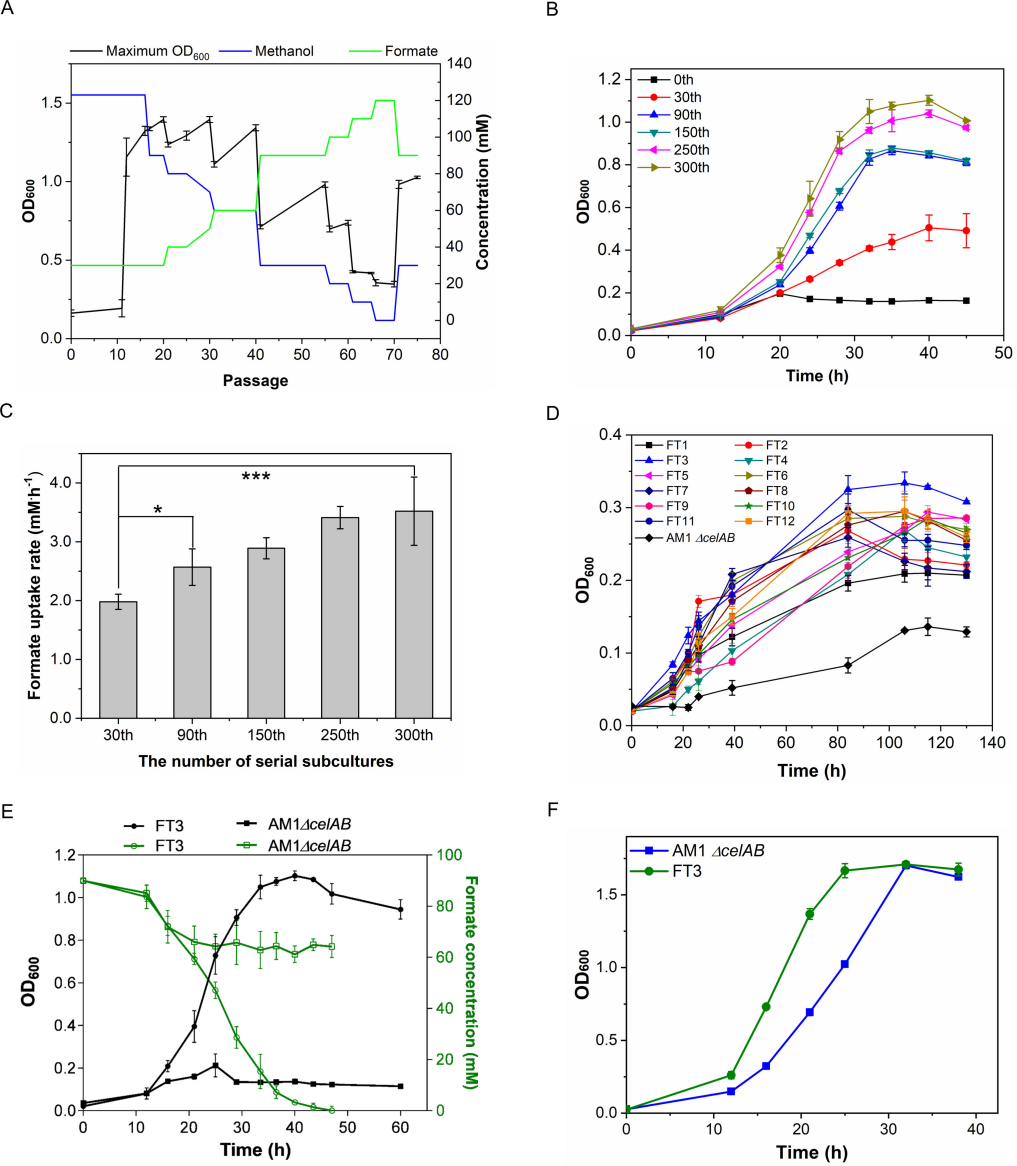
A summary of the evolved strains and an illustration of the isolated FT3 strain. (**A**) The scheme of evolution of the *M. extorquens* AM1 *ΔcelAB* strain. For the 10th passage, the OD_600_ values were presented as the average of 4 replicates, while for the other passages, the OD_600_ values were presented as the average of 12 replicates, with the standard deviations indicated as error bars. (**B**) Growth of the 12 evolved populations over different generations cultivated with 30 mM methanol and 90 mM formate, with standard deviations indicated as error bars. (**C**) Formate consumption rate of the 12 evolved populations across different generations cultivated with 30 mM methanol and 90 mM formate, with standard deviations indicated as error bars. (**D**) Growth of the 12 isolated strains cultivated with 120 mM formate. (**E**) A comparison of the growth and formate consumption rates between the FT3 strain and the parental *M. extorquens* AM1 *ΔcelAB* strain when cultivated with 30 mM methanol and 90 mM formate. (**F**) A comparison of the growth between the FT3 strain and the parental *M. extorquens* AM1 *ΔcelAB* strain when cultivated with 120 mM methanol. In panels D, E, and F, the data were presented as the average of three replicates, with standard deviations indicated as error bars. Statistical analysis was performed using a two-tailed Student’s *t*-test (*P < 0.05; ***P < 0.01).

ALE was employed to evolve *M. extorquens* AM1 to achieve tolerance to a high concentration of formate. The growth of *M. extorquens* AM1 is severely inhibited when formate is present at concentrations up to 15 mM as the sole carbon source. Consequently, during the ALE process, methanol is used as an additional carbon source. *M. extorquens* AM1 *ΔcelAB* (this deletion can prevent cell aggregation in laboratory cultures) (42) was first cultivated on 90 mM methanol and 30 mM formate. ALE was then carried out through serial transfer every 48 hours. After 10 passages of ALE, the maximum biomass of *M. extorquens* AM1*ΔcelAB* gradually increased from the optical density (OD_600_) value of 0.2 to 1.2. In subsequent passages, the concentration of formate incrementally increased and the concentration of methanol decreased accordingly (Fig. 1A). Eventually, the evolved strains were able to grow on the medium containing 30 mM methanol and 90 mM formate, reaching a maximum OD_600_ of 1.12, which was 5.3 times higher than the parental *M. extorquens* AM1 *ΔcelAB* strain (Fig. 1B). The maximum biomass and formate consumption rate of the evolved strains at the 30th, 90th, 150th, 250th, and 300th generations were measured by cultivating with 30 mM methanol and 90 mM formate, respectively (Fig. 1B and C). A comparison of the formate consumption rates at the 30th and 300th generations revealed an increase from 1.98 mM .h^−1^ to 3.52 mM .h^−1^ (Fig. 1C). The evolved strains at the 300th generation exhibited a significantly enhanced level of biomass and formate consumption rate compared to the parental strain when grown on 30 mM methanol and 90 mM formate. Therefore, each colony of the evolved strains was isolated at the 300th generation, and 12 individual colonies (named as the FT1 strain to the FT12 strain) were selected for evaluation by cultivating them with 30 mM methanol and 90 mM formate, as well as with 120 mM formate.

Among the 12 colonies, the evolved strain FT3 strain exhibited the best performance when cultivated on a medium containing either a mixture of 30 mM methanol and 90 mM formate or only 120 mM formate (Fig. 1D; Fig. S2). When a mixture of formate and methanol was used as the carbon sources, the FT3 strain completely exhausted the 30 mM formate within 48 hours, whereas the parental *M. extorquens* AM1 *ΔcelAB* strain exhibited a significantly lower consumption rate, with only 30% of the formate consumed (Fig. 1E). The FT3 strain achieved a maximum OD_600_ of 0.35 when 120 mM formate was used as the sole carbon source, which was approximately three times higher than that of the *M. extorquens* AM1 *ΔcelAB* strain (Fig. 1D). Moreover, when cultivated on a medium with methanol as the sole carbon source, the FT3 strain and the *M. extorquens* AM1 *ΔcelAB* strain exhibited similar biomass accumulation, but the FT3 strain displayed a specific growth rate of 0.14 h^−1^, which was 28% higher than that of the *M. extorquens* AM1 *ΔcelAB* strain (Fig. 1F). Notably, when cultivated with 120 mM methanol, the FT3 strain displayed a darker pink color compared to the *M. extorquens* AM1 *ΔcelAB* strain (Fig. S3), indicating enhanced synthesis of C_30_ carotenoid pigment in the cell membrane of the FT3 strain. In addition, when comparing to the carotenoids in the FT3 strain cultivated with 120 mM methanol as the sole carbon source, the amounts of C_30_ carotenoids in the FT3 strain grown with 90 mM methanol and 30 mM formate, or 60 mM methanol and 60 mM formate, increased to 1.86-fold and 2.72-fold, respectively (Fig. S4). This finding indicated that increasing the formate concentration could enhance the synthesis of C_30_ carotenoids in the FT3 strain.

**Fig 2:**
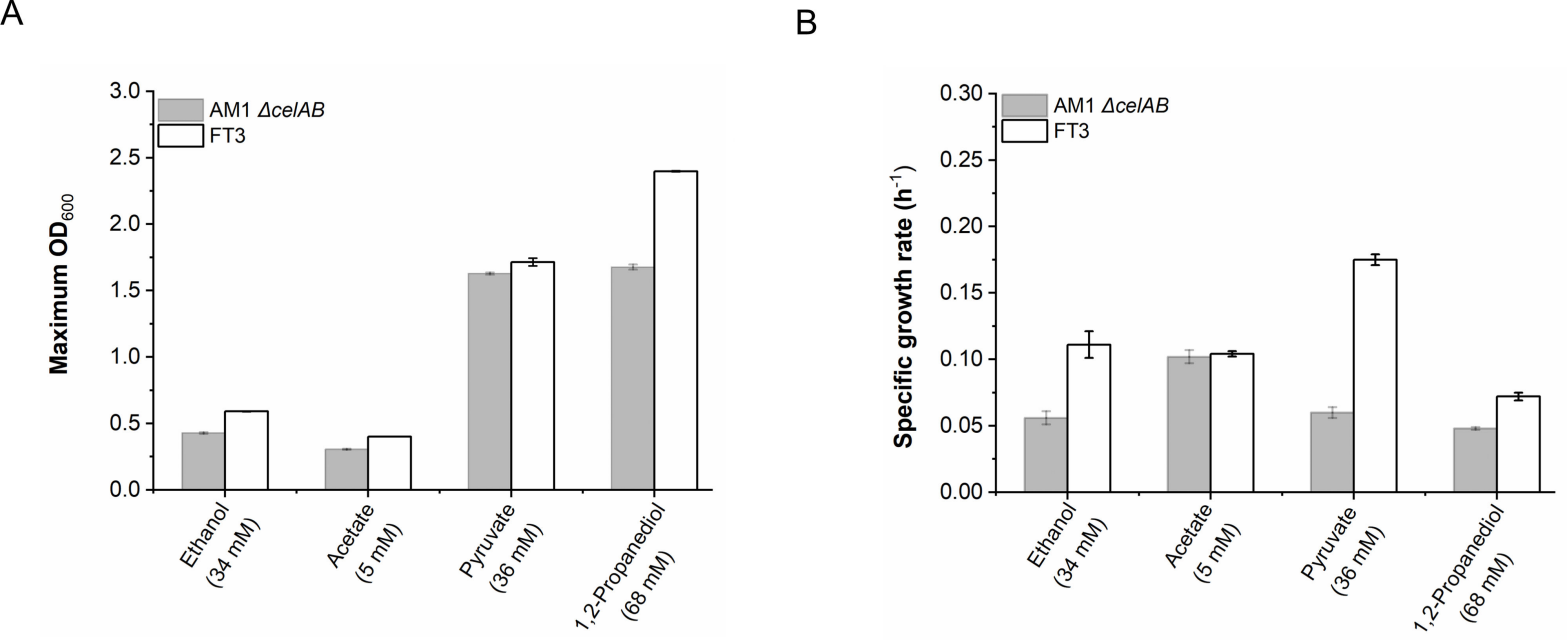
A comparison of the maximum OD_600_ values (**A**) and specific growth rates (**B**) of the FT3 strain and its parental strain on different carbon sources, including 34 mM ethanol, 5 mM acetate, 36 mM pyruvate, and 68 mM 1,2-propanediol. The data were presented as the average of three replicates, with standard deviations indicated as error bars.

Next, we investigated whether the FT3 strain also performed well when exposed to other weak organic acids and alcohols. As shown in Fig. 2, the specific growth rates of the FT3 strain when cultivated with 34 mM ethanol, 68 mM 1,2-propanediol, and 36 mM pyruvate were 0.111 h^−1^, 0.072 h^−1^, and 0.175 h^−1^, respectively. These rates were 1.98-, 1.5-, and 2.92-fold higher than those observed for the *M. extorquens* AM1 *ΔcelAB* strain. The maximum OD_600_ of the FT3 strain increased by 37.5% and 42.9% when cultivated with ethanol and 1,2-propanediol, respectively (Fig. 2; Fig. S5).

### Analyzing the ^13^C-labeled amino acids in the FT3 strain

**Fig 3:**
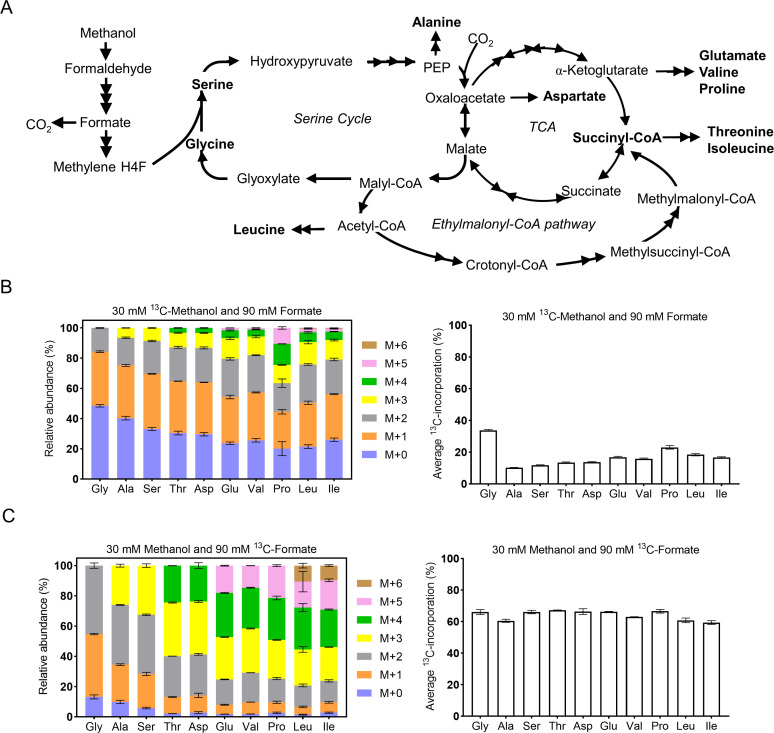
Analysis of the co-assimilation of methanol and formate using ^13^C-labeled carbon sources. (**A**) The 10 proteinogenic amino acids detected were synthesized from the intermediates involved in the serine cycle, TCA cycle, and ethylmalonyl-CoA pathway. (**B**) The labeling pattern and average carbon incorporation of proteinogenic amino acids in the FT3 strain cultivated with ^13^C-methanol and ^12^C-formate. (**C**) The labeling pattern and average carbon incorporation of proteinogenic amino acids in the FT3 strain cultivated with ^13^C-formate and ^12^C-methanol. M + X, where X indicates the number of ^13^C-labeled carbons. The data were presented as the average of three replicates, with standard deviations indicated as error bars.

To assess the incorporation of formate and methanol into cellular biomass by the FT3 strain, ^13^C-labeled method was conducted to analyze the proteinogenic amino acids. The FT3 strain was cultivated on media containing two different ^13^C-labeled carbon sources: (i) 30 mM ^13^C-methanol and 90 mM ^12^C-formate or (ii) 30 mM ^12^C-methanol and 90 mM ^13^C-formate. When the FT3 strain was grown to an OD_600_ value of about 0.6, the cells were harvested and subjected to hydrolysis for GC-MS analysis (Fig. S6). All 10 detected proteinogenic amino acids, belonging to four groups, were ^13^C-labeled. These included glycine, alanine, serine, glutamate, proline, aspartate, valine, threonine, leucine, and isoleucine (Fig. 3). The average carbon labeling ratio of 10 proteinogenic amino acids when cultured with 30 mM ^13^C-methanol and 90 mM ^12^C-formate ranged from 10.1% to 33.7%, which was consistent with the ratio (25%) of ^13^C-labeled carbon source. When cultivated with 30 mM ^12^C-methanol and 90 mM ^13^C-formate, the labeled ratios of these amino acids were found to range from 59.4% to 67.5%, which was also in line with the ratio (75%) of labeled carbon source. Overall, these results suggested that the FT3 strain was capable of effectively co-utilizing formate and methanol.

### Identification of the FT3 strain as a hypermutant strain with a considerable impact on the metabolic pathways

To uncover the mechanism of the increased tolerance and assimilation of formate, a comprehensive genomic and transcriptomic analysis was conducted on the FT3 strain. The genomic data indicated that the FT3 strain exhibited hypermutations, a trait that was different from the previously evolved strains derived from *M. extorquens* AM1 by ALE (43–46). A total of 5,551 single-nucleotide polymorphisms (SNPs) were identified in the coding sequence (CDS) regions of 459 genes, and 2,053 intergenic mutations and 31 insertion/deletion (Indel) mutations were identified in the genome of the FT3 strain (Data set S1). In addition to the proteins with unknown function, proteins with SNPs were classified into various pathways, including gluconeogenesis, oxidative phosphorylation, the serine cycle, the ethylmalonyl-CoA pathway (i.e., glyoxylate regeneration pathway), DNA replication and repair, secondary metabolism, amino acid metabolism, transporters, and enzymes involved in co-factor biosynthesis (Data set S1). The hypermutant property of the FT3 strain can be attributed to mutations in the DNA repair and DNA replication systems (Data set S1).

These SNPs, occurring within or in the intergenic regions of genes, exhibited significant impact on gene transcription. When the FT3 strain was cultivated on 150 mM methanol as the sole carbon source, a notable alteration in the transcription profile of the FT3 strain was observed in comparison to the *M. extorquens* AM1. Our findings revealed that 244 genes were upregulated and 2,430 genes were downregulated in the FT3 strain (Fig. S7). In the presence of 30 mM formate, the FT3 strain was found to exhibit only 244 upregulated genes and 90 downregulated genes (Fig. S8). It has been demonstrated that the majority of mutations are neutral, with only a small number conferring a beneficial allele (47). Consequently, identifying the pivotal genes involved in formate tolerance represented a substantial challenge. In this study, we employed the genomic and transcriptomic data to narrow down a list of candidate genes that were likely to be involved in formate tolerance and assimilation by identifying those that are in the central metabolic pathways.

#### Mutation of the FocA homologue META1_0287 enhanced the transport of formate

**Fig 4:**
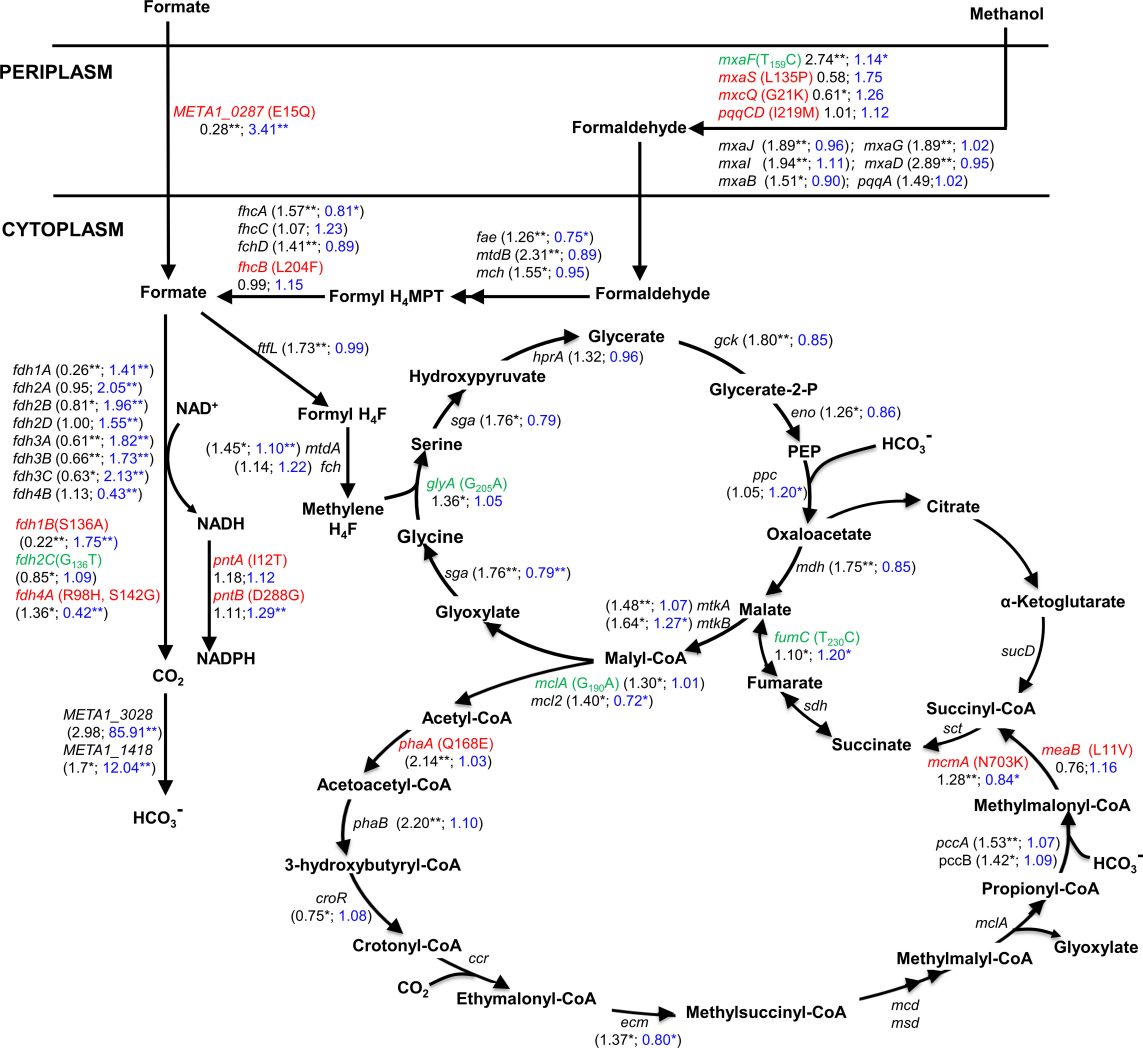
A detailed examination of the SNPs within the genomic and transcriptomic data of pivotal enzymes engaged in central metabolic pathways in the FT3 strain. Amino acid SNPs occurring in the CDS regions are marked in red, while nucleotide SNPs in the intergenic regions are marked in green. The data represented in black indicate the fold change in the gene transcription of the FT3 strain compared to the parental *M. extorquens* AM1 *ΔcelAB* strain, which was cultivated with 150 mM methanol. The fold change in gene transcription of the FT3 strain compared to the parental *M. extorquens* AM1 *ΔcelAB* strain in the presence of 120 mM methanol and 30 mM formate is shown with the data in blue. The data were presented as the average of three replicates. Significant differentially expressed genes were defined as having a (FDR) *P <0.05 and **P <0.01.

**Fig 5:**
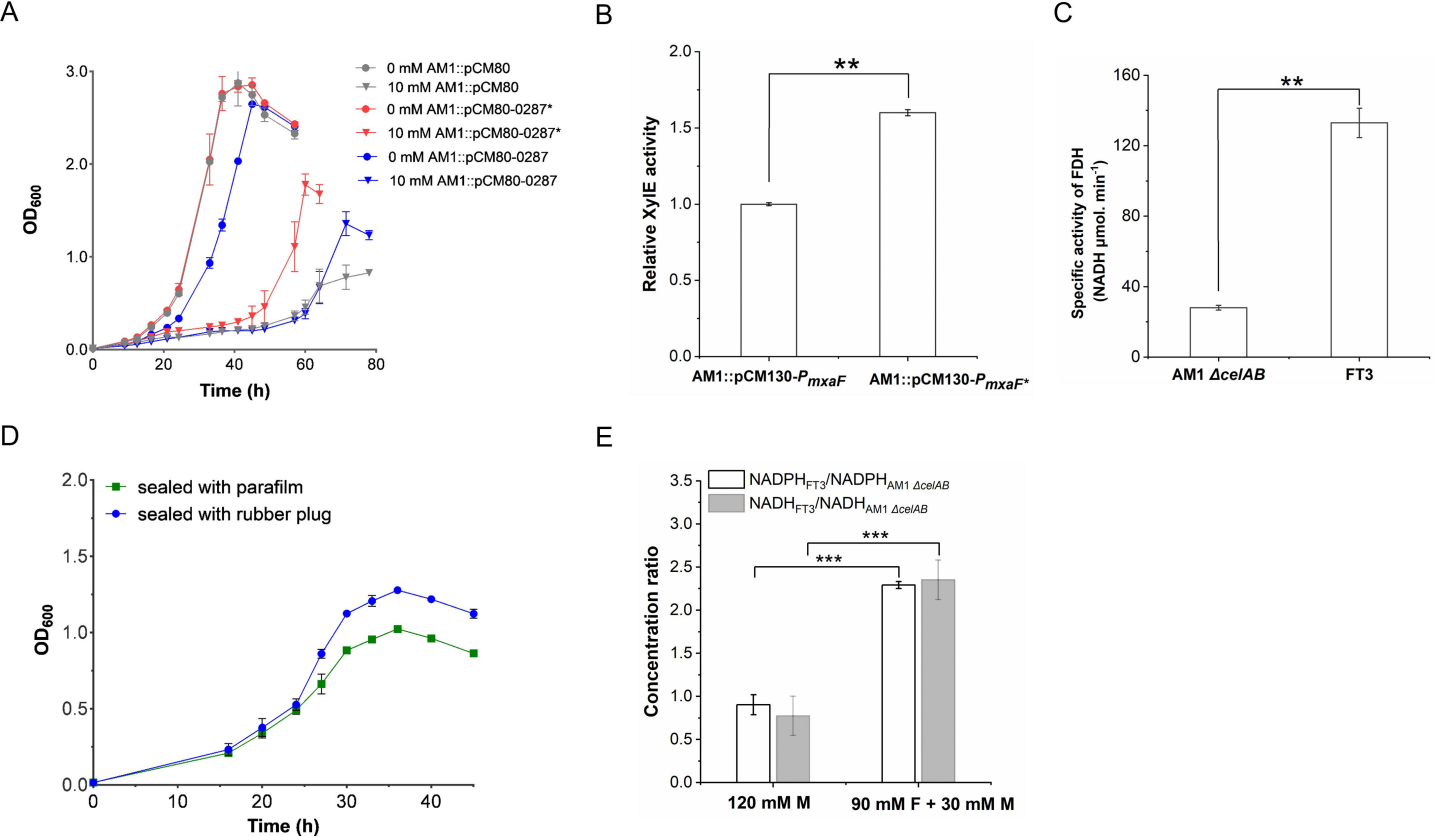
The characteristics of the *M. extorquens* AM1 derivative strains. (**A**) Growth curves of the engineered strains harboring plasmid pCM80-based overexpression of the native nitrate-formate transporter gene *META1_0287* and the mutated META1_0287* from *M. extorquens* FT3 in the presence of different formate concentrations. (**B**) Assessment of the promoter strength of *P*_mxaf_ from the *M. extorquens* AM1 strain and *P*_mxaf*_ (T_159_ to C_159_) from the FT3 strain through an *XylE*-based experiment. (**C**) Measurement of formate dehydrogenase (FDH) activity in the *M. extorquens* AM1 *ΔcelAB* strain and the FT3 strain. (**D**) Growth curves of the FT3 strain cultivated on medium containing 90 mM formate and 30 mM methanol in flasks with two different sealing methods. (**E**) Comparison of the ratios of NADH and NADPH between the FT3 strain and the *M. extorquens* AM1 *ΔcelAB* strain. The concentrations of NADPH and NADH in the *M. extorquens* AM1 *ΔcelAB* strain were determined by cultivation in a medium containing 120 mM methanol (M), while the FT3 strain was cultivated in a medium containing either 120 mM methanol (M) or 90 mM formate (F) in combination with 30 mM methanol (M). The data were presented as the average of three replicates, with standard deviations indicated as error bars. Statistical analysis was performed using a two-tailed Student’s *t*-test (**P < 0.01; ***P < 0.001).

A high concentration of formate can inhibit proton transfer across the cell membrane, which leads to the inhibition of ATP generation and consequently, the toxicity of cells (48, 49). To detoxify high concentrations of formate, it is necessary for formate to be transported into the cell and subsequently assimilated by the FT3 strain. The nitrate-formate transporter FocA and oxalate:formate antiporter have been reported to be capable of transporting formate into the cell (50, 51). Mutations have also been observed in the FocA homologue protein META1_0287 (E5Q) and the oxalate:formate antiporter META1_0992 in the FT3 strain (Data set S1). The transcription level of *META1_0992* was considerably lower than that of *META1_0287* when the FT3 strain was cultivated with 150 mM methanol. The transcriptional level of *META1_0287* in the FT3 strain was observed to significantly increase to 3.41-fold in the presence of 30 mM formate (Fig. 4; Data set S2). Based on these findings, it was hypothesized that FocA homolog META1_0287 was responsible for the transport of formate into the cell in the FT3 strain. To test this hypothesis, the native *META1_0287* and its mutated META1_0287* were overexpressed in the *M. extorquens* AM1 strain. Subsequently, the strains were cultivated on the medium with 120 mM methanol as the sole carbon source. The strain AM1::pCM80-0287* exhibited a similar specific growth rate to the control strain AM1::pCM80, which was slightly higher than the specific growth rate of the AM1::pCM80-0287 strain (Fig. 5A). The addition of 10 mM formate resulted in a shorter lag phase (30 hours) for the strain AM1::pCM80-0287* compared to both the strain AM1::pCM80 (60 hours) and the strain AM1::pCM80-0287 (60 hours) (Fig. 5A). Moreover, the strain AM1::pCM80-0287* reached a maximum OD_600_ value of 1.78, which was significantly higher than that of the strain AM1::pCM80-0287 and AM1::pCM80 (Fig. 5A). These results indicated that the E5Q mutation in the *N*-terminal of FocA homolog META1_0287* played a crucial role in formate tolerance. It has been demonstrated that FocA is capable of transporting formate in a pH-dependent manner, with the ability to transport formate in both protonated and neutralized forms (52). The presence of 10 mM sodium formate resulted in a pH increase from 7.27 to 8.15 within the first 25 hours (Fig. S9), suggesting that formate was transported into the cell in its protonated formic acid state, as previously reported (52).

#### Metabolic pathway of methanol oxidation to formate was significantly affected

The metabolic pathway of methanol oxidation to formate was found to be significantly affected. A number of mutations were identified in several regions, including the CDS region of the methanol dehydrogenase system MxaS (L135P) and MxcQ (G21K), the PQQ synthase PqqCD (I219M), and the formyltransferase/hydrolase complex Fhc (L204F) (Fig. 4). Moreover, an intergenic SNP mutation, T_159_ to C_159_, located 159 base pairs upstream of the methanol dehydrogenase gene *mxaF* was found (Fig. 4). The XylE-based experiment demonstrated that the T_159_ to C_159_ mutation resulted in a 60% increase in the promoter strength of *mxaF* (Fig. 5B). However, the engineered strain AM1-MxaF* harboring C_159_ mutation showed no enhancement on formate tolerance (Fig. S10), suggesting this mutation may not be related to formate tolerance or formate utilization. In comparison to the *M. extorquens* AM1 grown on 150 mM methanol, the transcriptomic data indicated that the transcription of the majority of genes involved in this process in the FT3 strain was enhanced, particularly *mxaFJGI*, *mxaD*, and *pqqA*. This suggested an increased conversion of methanol to formaldehyde (Fig. 4; Data set S2). However, when exposed to 30 mM formate, the transcriptional levels of *fae* and *fhcA* were significantly downregulated to 0.75- and 0.81-fold, indicating a decreased conversion of formaldehyde to formate (Fig. 4). The cultivation of the FT3 strain resulted in the increased absorption of formate by the mutated FocA homologue META1_0287* and an increase in the cellular formate concentration. Consequently, the conversion of formaldehyde to formate was decreased, thereby alleviating the formate stress. The enhanced conversion from methanol to formaldehyde and the decreased conversion from formaldehyde to formate led to an increased concentration of formaldehyde, suggesting that the FT3 strain could exhibit a higher tolerance to formaldehyde. Further investigation into formaldehyde tolerance also demonstrated that the FT3 strain exhibited tolerance up to 10 mM formaldehyde, whereas the *M. extorquens* AM1 *ΔcelAB* strain showed tolerance up to 7.5 mM formaldehyde (Fig. S11).

#### Pathways related to formate metabolism were notably impacted in the FT3 strain

Formate can be converted to CO_2_ or be assimilated into the cell through the serine cycle coupled to the ethylmalonyl-CoA pathway. The mutations involved in the formate assimilation pathways included the CDS regions of β-ketothiolase PhaA (Q168E), methylmalonyl-CoA mutase McmA (N703K), and succinyl-CoA/methylmalonyl-CoA mutase accessory protein MeaB (L11V). Moreover, the intergenic SNPs were observed in the serine hydroxymethyltransferase gene *glyA* (G_205_ to A_205_), malyl-CoA thioesterase gene *mclA* (G_190_ to A_190_), and fumarase gene *fumC* (T_230_ to C_230_) (Fig. 4). Transcriptomic data further revealed that most of the genes involved in the formate assimilation pathways exhibited enhanced transcription in the FT3 strain grown on 150 mM methanol, especially for *ftfL*, *glyA,* and *mtkAB* (Fig. 4), which are three key enzymes involved in the formate assimilation pathway (53). These genes were upregulated to 1.73-, 1.36-, and 1.64-fold, respectively (Fig. 4). Concerning the oxidation of the formate pathway, it was observed that SNP mutations occurred in the CDS region of the FDH genes of *fdh4A* (R98H and S142G) and *fdh1B* (S36A), as well as in the intergenic region of the *fdh2CBAD* operon (Fig. 4). Subsequently, the XylE-based experiments demonstrated that the G_136_ to T_136_ mutation located upstream of the *fdh2CBAD* operon had no influence on promoter strength, which was consistent with the transcriptomic data (Fig. S12). Notably, the transcription levels of *fdh1A*, *fdh1B*, *fdh3A*, *fdh3B,* and *fdh3C* in the FT3 strain grown on methanol were all downregulated significantly, with expression levels reduced to 0.26-, 0.22-, 0.61-, 0.66-, and 0.63-fold, respectively (Fig. 4). In the presence of 30 mM formate, the transcription levels of *fdh1B*, *fdh2A*, *fdh2B*, *fdh2D*, *fdh3A*, *fdh3B,* and *fdh3C* in the FT3 strain were upregulated significantly, whereas only *fdh4A* and *fdh4B* were downregulated to 0.42- and 0.43-fold, respectively (Fig. 4). Given the enhanced transcription of most of the FDH genes, we investigated whether the activity of FDH in the FT3 strain was also elevated. When compared to *M. extorquens* AM1 cultivated with 120 mM methanol, the specific activity of FDH in the FT3 strain cultivated with 90 mM methanol and 30 mM formate was 4.75 times higher than that in *M. extorquens* AM1 (Fig. 5C). As there were no significant changes in the transcription levels of the genes involved in the formate assimilation pathway in the presence of 30 mM formate, we speculated that the enhanced conversion of formate to CO_2_ resulted in increased carbon loss. This was supported by the previous observation that the maximum biomass of the FT3 strain was substantially decreased when cultivated on 90 mM formate and 30 mM methanol (Fig. 1D).

Interestingly, when exposed to 30 mM formate, two genes coding carbonate dehydratases *META1_3028* and *META1_1418*, involved in reversible hydration of dissolved CO_2_ into carbonic acid, showed significant upregulation (Fig. 4). Therefore, we hypothesized that the FT3 strain utilized these two carbonate dehydratases to facilitate the dissolution of CO_2_ within the cell, whereby bicarbonate was formed. This can then be catalyzed by PEP carboxylase Ppc or propionyl-CoA carboxylase PccAB to synthesize oxaloacetate or methylmalonyl-CoA (Fig. 4). Furthermore, the cultivation of the FT3 strain in flasks sealed with either parafilm or a rubber plug demonstrated that, compared to the flask sealed with parafilm, the growth rate and maximum biomass of the FT3 strain increased by 11% and 21.4%, respectively, in flasks sealed with a rubber plug when 30 mM formate and 90 mM methanol were used as the carbon sources (Fig. 5D). When methanol was used as the sole carbon source, no difference was observed in the FT3 strain cultivated with flasks sealed with either parafilm or a rubber plug (Fig. S13). These results indicated that the emission of CO_2_ from the flask can be prevented by a rubber plug, which can be readily dissolved in the cell by enhanced carbonate dehydratase and subsequently utilized for intermediate synthesis, thereby improving biomass production.

The enhanced formate oxidation pathway could result in a high production of NADH, which could subsequently be converted to NADPH by the membrane-bound NADH/NADPH transhydrogenase. The mutations were observed in the transhydrogenases PntA (I12T) and PntB (D288G) (Fig. 4). When exposed to 30 mM formate, *pntB* was found to be upregulated to 1.29-fold (Fig. 4). Furthermore, we analyzed the pools of reducing equivalents in the FT3 strain. When the FT3 strain was cultivated with 120 mM methanol as the sole carbon source, the concentrations of NADH and NADPH were slightly lower than those of the *M. extorquens* AM1 *ΔcelAB* strain (Fig. 5E). However, when the FT3 strain was cultivated in the presence of 30 mM formate, the concentrations of NADH and NADPH increased to 3.04-fold and 2.54-fold, respectively, in comparison with that grown on 120 mM methanol (Fig. 5E). Moreover, the concentrations of NADH and NADPH in the FT3 strain cultivated with 30 mM formate and 90 mM methanol were found to be 2.29-fold and 2.35-fold higher, respectively, than those observed in the *M. extorquens* AM1 *ΔcelAB* strain grown on methanol (Fig. 5E). These results suggested that the production of reduced equivalents was improved in the FT3 strain with the addition of formate.

### Assessing the genes that conferred tolerance to formate by using an ALE-inspired overexpression method

**Fig 6:**
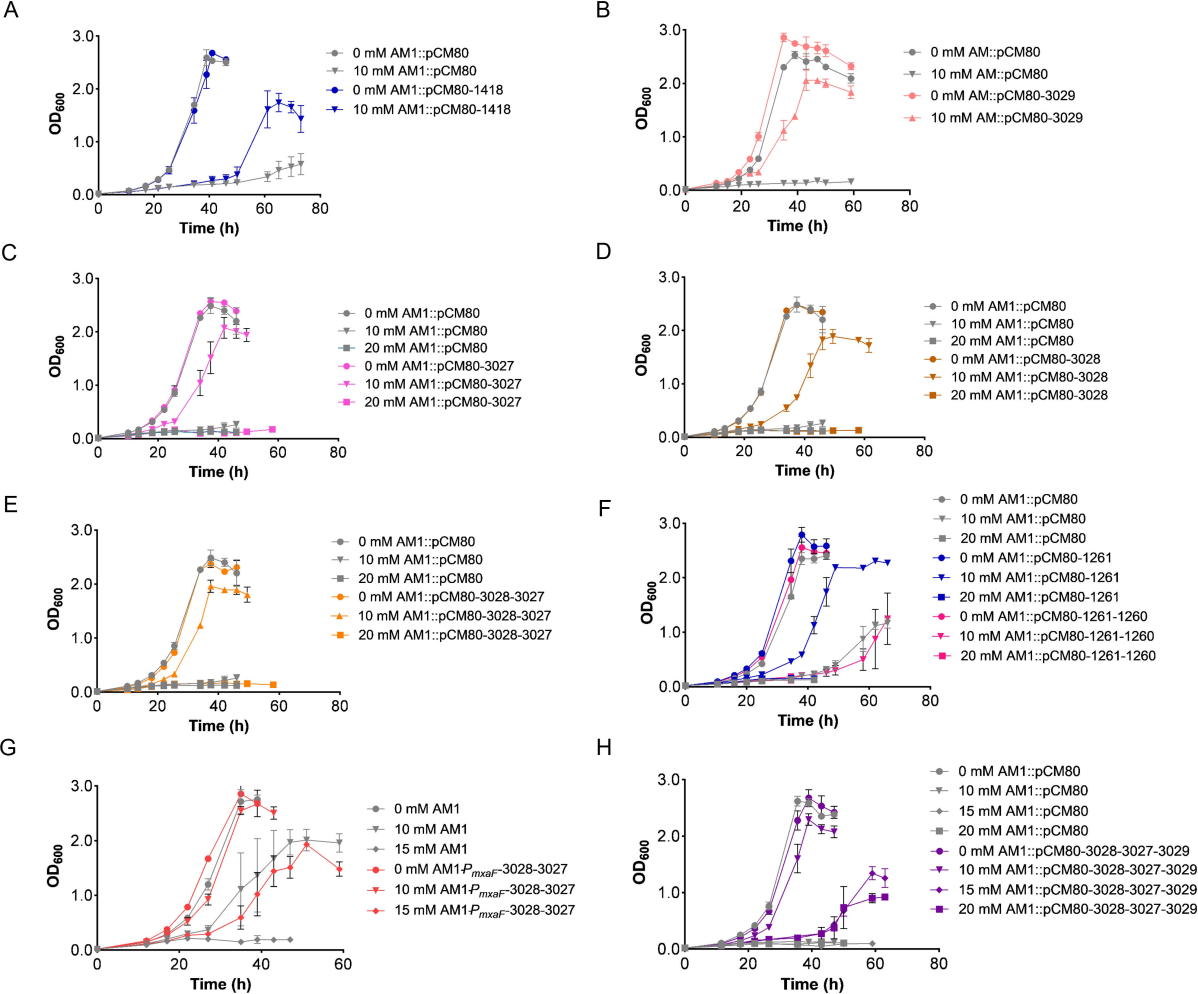
Investigation of the formate tolerance through the overexpression of selected genes in *M. extorquens* AM1. The growth curves of the *M. extorquens* AM1 derivative strains harboring plasmid pCM80, which overexpressed *META1_1418* (**A**), *META1_3029* (**B**), *META1_3027* (**C**), *META1_3028* (**D**), or the operon *META1_3028* and *META1_3027* (**E**), *META1_1261*, or the operon *META1_1261* and *META1_1260* (**F**), and the artificial operon of *META1_3027*, *META1_3028*, and *META1_3029* (**H**) in different formate concentrations. (**G**) The growth curves of the engineered strain *M. extorquens* AM1-*P*_maxF_-3028-3027, in which the operon *META1_3028* and *META1_3027* were driven by the promoter *P*_maxF_. The data were presented as the average of three replicates, with standard deviations indicated as error bars.

Among the top 20 significantly upregulated genes in the FT3 strain in the presence of 30 mM formate, two carbonate dehydratase genes (*META1_3028* and *META1_1418*), a transporter gene *META1_3027*, an RNA polymerase sigma factor (*META1_1261*), and a transmembrane anti-sigma factor (*META1_1260*) were observed (Data set S2), which were found to be upregulated to 85.9-, 12.0-, 82.2-, 18.4-, and 32.6-fold, respectively. Furthermore, five hypothetical protein genes *META1_3029, META1_3458, META1_2965, META1_2964,* and *META1_0394* were upregulated to 90.3-, 222.7-, 49.8-, 95.0-, and 7.5-fold, respectively. These genes were then overexpressed in *M. extorquens* AM1, respectively. Derivative strains with overexpression of *META1_3027*, *META1_3028*, *META1_3029*, *META1_1261*, and *META1_1418* demonstrated enhanced growth in the presence of 10 mM formate compared to the *M. extorquens* AM1 strain with empty plasmid pCM80, indicating their involvement in formate tolerance (Table 2; Fig. 6). The strain AM1::pCM80-1261 displayed a maximum OD_600_ value of 2.32, followed by AM1::pCM80-3027 (OD_600_ = 2.08), and AM1::pCM80-3029 (OD_600_ = 2.06) (Table 2). Compared to the strains with overexpression of a single gene, the superior performance of the FT3 strain at elevated formate levels suggested that the capacity to tolerate high levels of formate was conferred by the action of multiple genes.

**TABLE 2:** Specific growth rates and maximum OD_600_ values of the *M. extorquens* AM1 derivative strains

Strain	Overexpression of genes	Formate concentration (mM)	Specific growth rate (h^−1^)	Maximum OD_600_
AM1::pCM80-1261	*META1_1261*	10	0.124 ± 0.010	2.32 ± 0.02
AM1::pCM80-3027	*META1_3027*	10	0.121 ± 0.010	2.08 ± 0.21
AM1::pCM80-3029	*META1_3029*	10	0.094 ± 0.004	2.06 ± 0.01
AM1::pCM80-1418	*META1_1418*	10	0.088 ± 0.006	1.74 ± 0.18
AM1::pCM80-3028	*META1_3028*	10	0.118 ± 0.016	1.89 ± 0.132
AM1::pCM80-3028-3027	*META1_3028* and *META1_3027*	10	0.154 ± 0.010	1.89 ± 0.04
AM1::pCM80-3028-3027-3029	*META1_3028*, *META1_3027,* and *META1_3029*	10	0.151 ± 0.003	2.30 ± 0.07
		15	0.093 ± 0.047	1.40 ± 0.119
		20	0.070 ± 0.020	0.93 ± 0.06
AM1-*p_maxF_*-3028-3027	*META1_3028* and *META1_3027*	15	0.073 ± 0.009	1.93 ± 0.02

The genes *META1_3027* and *META1_3028* are assembled as an operon in the genome, as are the genes *META1_1261* and *META1_1260*. Based on the results of the overexpression of individual gene, then, these two operons were further co-expressed in *M. extorquens* AM1 (Fig. 6E and F). The strain AM1::pCM80-1261-1260 failed to improve growth in the medium containing 10 mM formate compared to the overexpression of the individual gene *META1_1261*, suggesting that this operon may not be involved in formate tolerance (Fig. 6F). The specific growth rate of the strain AM1::pCM80-3028-3027 was 0.154 h^−1^ in the presence of 10 mM formate, which was higher than that of the strain AM1::pCM80-3028 or AM1::pCM80-3027 (Fig. 6E; Table 2). This indicated that the overexpression of this operon exhibited better performance at high concentrations of formate. Furthermore, the native promoter of the *META1_3027* and *META1_3028* operon was replaced with the strong promoter *P_mxaf_* to initiate the expression. The engineered strain *M. extorquens* AM1-*P_mxaF_*-3028-3027 demonstrated tolerance to 15 mM formate, showing a specific growth rate value of 0.073 h^−1^ and a maximum OD_600_ value of 1.93 (Fig. 6G). The gene *META1_3029* is adjacent to *META1_3027* and *META1_3028* (Fig. S14), and overexpression of this gene was shown to enhance tolerance to formate (Fig. 6B). Consequently, the genes *META1_3027*, *META1_3028*, and *META1_3029* as an artificial operon were overexpressed in *M. extorquens* AM1. The strain AM1::pCM80-3028-3027-3029 was found to be able to grow on the medium containing 15 mM formate, exhibiting a specific growth rate of 0.093 h^−1^ and reaching a maximum OD_600_ value of 1.40 (Fig. 6H). Notably, in the presence of 20 mM formate, the AM1::pCM80-3028-3027-3029 exhibited a specific growth rate of 0.07 h^−1^ and a maximum OD_600_ value of 0.93 (Table 2). The results obtained from the combination of overexpressing the transporter gene *META1_3027*, the carbonate dehydratase gene *META1_3028*, and the hypothetical protein gene *META1_3029* demonstrated a synergistic effect‌, suggesting that these three genes were likely involved in formate tolerance and utilization.

### FT3 strain as a chassis with mixed methanol and formate to produce reduced chemicals of 3-HP

**Fig 7:**
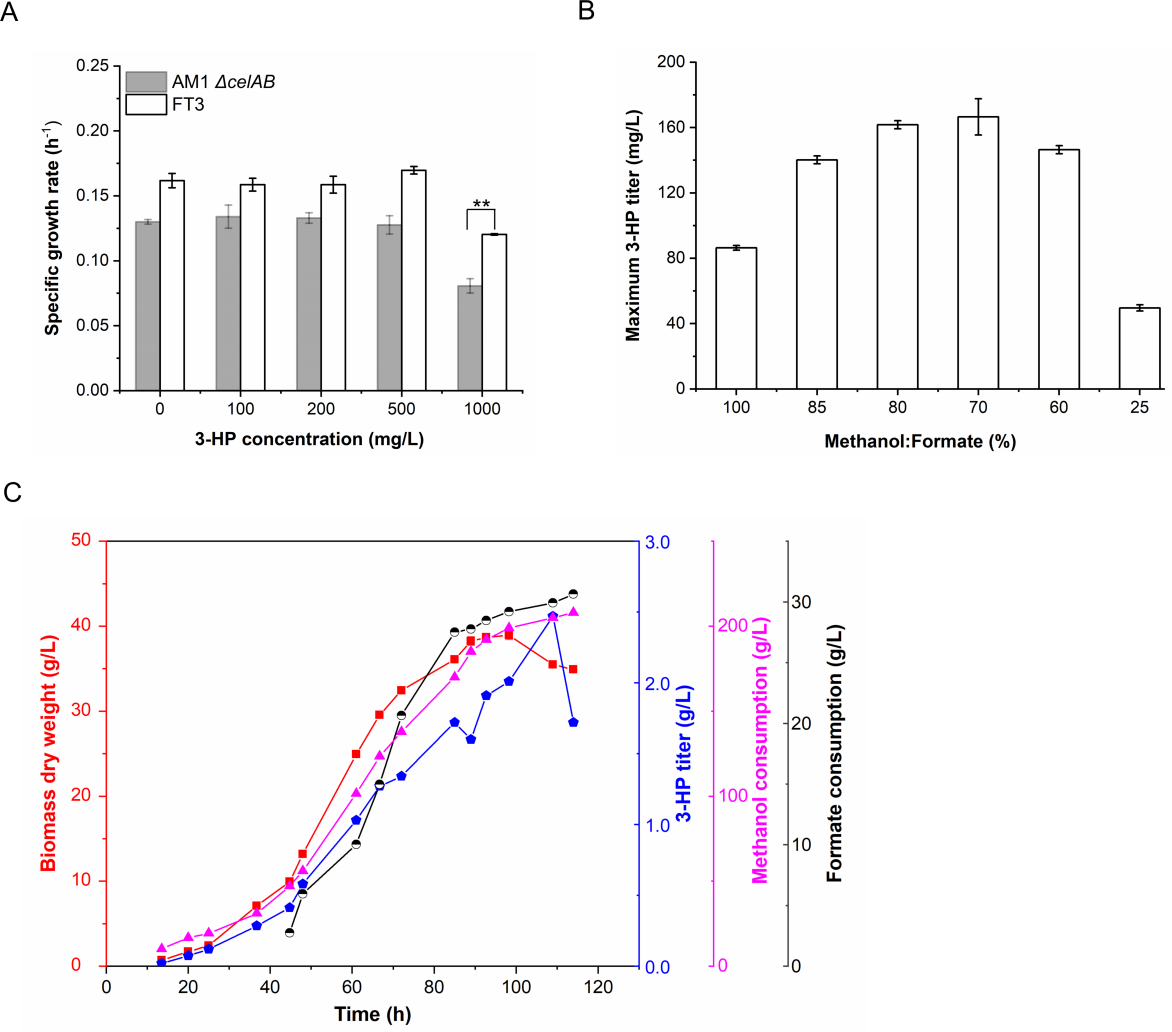
The FT3 strain used as the host chassis to produce 3-HP. (**A**) Investigation of the tolerance of the FT3 strain to 3-HP. (**B**) Investigation of the optimal ratio of methanol to formate to produce 3-HP in the FT3::pCM80-Apr-mcr strain. (**C**) Analysis of 3-HP production in the FT3::pCM80-Apr-mcr strain using fed-batch fermentation with the mixed carbon sources of methanol (0.3%, wt/vol) and formate (in the range of 8 to 12.7 mM). The data in B and C were presented as the average of three replicates, with standard deviations indicated as error bars. Statistical analysis was performed using a two-tailed Student’s t-test (*P < 0.05; **P < 0.01).

Previously, *M. extorquens* AM1 was identified as a chassis capable of producing reduced chemicals of 3-HP with methanol as the sole carbon source (33, 36, 40). In the present study, a comparison of the *M. extorquens* AM1 strain and the FT3 strain revealed that the latter exhibited better tolerance to 3-HP. When exposed to 1,000 mg/L 3-HP, the growth rate of the FT3 strain was 0.120 h^−1^, representing a 1.48-fold higher rate than *M. extorquens* AM1 (Fig. 7A). Based on the better tolerance to 3-HP and increased pools of reducing equivalents in the FT3 strain in the presence of formate, we speculated that the FT3 strain was a more suitable chassis for the production of 3-HP. The plasmid pCM80-Apr-mcr containing a 3-HP synthetic pathway was introduced into the FT3 strain, generating the FT3::pCM80-Apr-mcr strain. The production of 3-HP was quantified when the FT3 strain was grown on a mixture of carbon sources with varying ratios of methanol to formate. Methanol was used as the sole carbon source, serving as the control. The addition of formate at concentrations within the range of 22.5 mM to 60 mM (i.e., a reduction in the methanol to formate ratio from 85% to 70%) resulted in a continuous increase in 3-HP titer, reaching a range of 110 mg/L to 175 mg/L in flask culture (Fig. 7B). The highest titer of 175 mg/L was achieved when the FT3::pCM80-Apr-mcr strain was cultivated with 45 mM formate and 105 mM methanol. This titer was significantly elevated in comparison to the methanol, which served as the sole carbon source (Fig. 7B). Furthermore, fed-batch fermentation was conducted in a 3L biofermentor by using mixed carbon sources of methanol (0.3%, wt/vol) and formate (in the range of 8–12.7 mM). As shown in Fig. 7C, following a cultivation of 98 hours, the maximum biomass of the FT3::pCM80-Apr-mcr strain reached 38.9 g/L. After 109 hours of cultivation, the maximum titer of 3-HP was up to 2.47 g/L. This titer was approximately 14-fold higher than the highest titer obtained by shake-flask cultivation and also exceeded our previously reported production in the engineered *M. extorquens* AM1 strain (40). The current yield and productivity of 3-HP remain relatively low. Engineering of the one-carbon assimilation pathway will be required to further increase the one-carbon flow to the target product (40).

## DISCUSSION

Formate can be electrochemically converted from CO_2_ and represents a promising feedstock for biorefinery applications (3–7). Over the past decade, considerable research has been conducted to utilize formate as a carbon source for the production of high-value chemicals (15, 28–31, 54). In this study, the ALE strategy was employed to obtain an evolved strain FT3 derived from *M. extorquens* AM1, which demonstrated enhanced tolerance and assimilation of high concentrations of formate. Furthermore, this strain was proven to be a more effective chassis for producing 3-HP through the utilization of a combined methanol and formate as the carbon sources.

ALE is a frequently utilized methodology for the evolution of *M. extorquens* AM1 with new properties (43–46, 55). Typically, these evolved strains exhibit a few SNP mutations (43–46, 55). For example, an engineered strain with modified central metabolism that employs glutathione as a formaldehyde transporter, instead of the H_4_MPT pathway, demonstrates four SNP mutations (44). Similarly, an evolved strain with high tolerance to butanol concentration exhibits only one SNP mutation (45). However, this study revealed that the evolved strain FT3, which was identified for its tolerance to high formate concentration, displayed the hypermutant phenomenon. To our knowledge, this represents the first report of hypermutations occurring in *M. extorquens* AM1. Genome re-sequencing indicated that these hypermutations may be attributed to mutations in enzymes responsible for DNA repair and DNA synthesis systems. Hypermutability facilitates enhanced ALE fitness and allows for significant advancements across the fitness landscape by providing a diverse range of mutations with complex epistatic interactions (56, 57). However, the hypermutant strains could exhibit disadvantages regarding genome stability and adaptive potential. Further evaluation is required to ascertain whether the FT3 strain may continue to evolve as a result of mutations present in the DNA repair and replication systems. In the FT3 strain, the majority of mutations were classified as neutral, while a limited number were identified as beneficial alleles. Accordingly, the present study was primarily concerned with investigating the mutations involved in methanol and formate oxidation, formate transport, formate assimilation, and adjacent pathways. Based on the genomic and transcriptomic data, a number of genes were selected for further characterization through ALE-inspired gene manipulation experiments. The results indicated that the high formate tolerance observed in the FT3 strain was attributed to the action of multiple genes. The subsequent discussion is presented below.

The mutation of FocA has been demonstrated to be important for formate tolerance (20). In the present study, it was observed that the mutated FocA homologue protein META1_0287 conferred tolerance to formate in the FT3 strain by increasing the uptake of formate into the cell. Subsequently, the cellular formate can enter either the formate oxidation pathway or the assimilation pathway. Previous studies have demonstrated that enhancing either the formate assimilation pathway, such as through the overexpression of the *ftfL* gene, or the formate oxidation pathway, such as through the overexpression or upregulation of the *fdh* gene, can improve formate tolerance (21, 24, 29, 30, 58–60). This study demonstrated that the formate oxidation pathway played a more important role in conferring tolerance to formate in the FT3 strain, as evidenced by the significant enhancement of formate dehydrogenase activity (Fig. 5C), while the transcription of key genes involved in the formate assimilation pathway remained unaltered in the presence of formate (Fig. 4). The addition of formate has been demonstrated to significantly enhance the level of reduced equivalents in *M. extorquens* AM1 (28). Furthermore, this study revealed that the FT3 strain exhibited an increased level of NADH and NADPH when exposed to 30 mM formate (Fig. 5E). It was postulated that the enhanced oxidation of formate resulted in the generation of a greater number of NADH, which could be further converted to NADPH by the PntAB enzyme to form NADPH. In synthetic formatotrophic bacteria that harbor the reductive glycine pathway, it has been demonstrated that enhancing the conversion of NADH to NADPH by PntAB is an important factor for growth on formate (24). It would be beneficial to investigate whether the mutation of PntAB in the FT3 strain plays a similar role in formate tolerance and assimilation.

The enhanced formate dehydrogenase activities resulted in a faster conversion of formate to CO_2_, a portion of which can be mobilized by the two carbonate dehydratases, META1_3028 and META1_1418, to form bicarbonate and a proton that can be used as an intermediate for regulating cellular pH. The direction of formate transportation by FocA is regulated by intracellular pH (52). It was therefore postulated that the cellular pH affected by these two carbonate dehydratases may be related to the transportation of formate directed by the mutated FocA homolog META1_0287*. This may explain why the carbonate dehydratases META1_3028 and META1_1418 were observed to exhibit formate tolerance (Fig. 6A and D). On the other hand, the bicarbonate converted from CO_2_ within the cell can be catalyzed by Ppc or PccAB to produce corresponding metabolites, oxaloacetate or methylmalonyl-CoA. Indeed, enhanced biomass was observed when the FT3 strain was cultivated with 30 mM formate in flasks sealed with a rubber plug (Fig. 5D), suggesting that blocking CO_2_ emission can improve biomass accumulation. The FT3 strain displayed an improved growth when cultivated with 120 mM formate as the sole carbon source, although the maximum OD_600_ value remained low (Fig. 1D). It can therefore be posited that enhancing the concentration of CO_2_ and enhancing the CO_2_ fixation may prove an effective way to improve the biomass accumulation in the FT3 strain cultivated with formate as the sole carbon source. It has previously been demonstrated that the synthetic formatotrophs are capable of achieving enhanced growth when formate and a high concentration of CO_2_ or bicarbonate are utilized (22, 24). This study presents an additional strategy to modify the native formatotrophs to promote growth in high concentrations of formate.

It has been demonstrated that alterations in the composition of the cell membrane are crucial for improving resistance to various abiotic stresses (61). Hopanoids and C_30_ carotenoids are integral components of the cell membrane in *M. extorquens* AM1 (62). Notably, hopanoids play a pivotal role in maintaining the stability and permeability of the cell membrane (62). Strains deficient in hopanoids exhibit increased membrane permeability, resulting in poor growth (63). Recent research has also demonstrated that reduced membrane permeability and altered membrane composition are important for formaldehyde-acclimated *M. extorquens* strains (64). In this study, exposure of the FT3 strain to formate increased the quantity of C_30_ carotenoid pigments. The transcriptomic data indicated that the transcriptional levels of *META1_1817*, which is involved in the synthesis of the precursor squalene, as well as *META1_3665*, *META1_3670*, *META1_3663*, and *META1_3664*, which are involved in the post-modification pathway of carotenoid pigment synthesis (62, 65), showed an increase in the presence of 30 mM formate (Fig. S15; Data set S2). This finding corresponds with the enhanced C30 terpenoid synthesis (Fig. S3). In *M. extorquens* AM1, hopanoids share the common precursor squalene with the C_30_ carotenoid pigments. Notably, the transcription of the squalene-hopene cyclase gene *shc* (*META1_1818*), a critical enzyme responsible for hopanoid synthesis, was approximately three times higher in the FT3 strain than in the *M. extorquens* AM1 *ΔcelAB* strain cultivated solely with methanol (Fig. S15; Data set S2). Given the significantly increased transcription of *shc* in the FT3 strain, it is reasonable to propose that hopanoid synthesis has also increased in the FT3 strain, potentially influencing the permeability of the cell membrane to adapt to environmental conditions, which may be related to the enhanced formate tolerance.

In *Cupriavidus necator* H16, the deletion of the transcriptional regulator PhcA has been shown to result in reduced expression of several operons, thereby enhancing growth on formate (19). Based on this observation, we hypothesized that alterations in the expression of certain operons in the FT3 strain would improve the formate tolerance. It is well established that sigma factors are involved in genome-wide regulatory processes. In our research, we found a significant increase in the transcription level of the sigma factor META1_1261 in the FT3 strain, indicating its potential role as a regulator of key genes associated with formate tolerance. Indeed, overexpression of the *META1_1261* gene was found to enhance formate tolerance (Fig. 6F). *META1_1261* and the anti-sigma factor *META1_1260* were identified as components of an operon. However, overexpression of this operon resulted in poor growth in the medium containing 10 mM formate compared to the overexpression of individual gene *META1_1261* (Fig. 6F). This implies that the anti-sigma factor *META1_1260* within the operon impairs the function of *META1_1261*. These results suggest that FT3 may have a complex regulatory network involved in formate tolerance. Transporters have been shown to relate to formate tolerance; for example, an A269T SNP mutation in the bicarbonate transporter SO_3578 enables it to transport formate, conferring *Shewanella oneidensis* with tolerance to 100 mM formate (66). In this study, we observed that overexpression of the sulfate transporter META1_3027 can also enhance tolerance to formate (Fig. 6C), although whether META1_3027 is involved in formate transportation requires further investigation. In addition, upon exposure to formate, certain genes with unknown functions, such as META1_3029, exhibited high levels of transcription and have been confirmed to enhance formate tolerance, which warrants further investigation in the future.

### Conclusion

In this study, an evolved FT3 strain derived from *M. extorquens* AM1, exhibiting a high tolerance to formate, was obtained through the application of the ALE strategy. Further feeding experiments with ^13^C-labeled one-carbon sources demonstrated that the FT3 strain was capable of effectively utilizing methanol and formate. In contrast to previously reported evolved strains of *M. extorquens* AM1, the FT3 strain exhibited hypermutations. Furthermore, a combination of DNA re-sequencing, transcriptome analysis, and ALE-inspired gene manipulation was employed to investigate the potential mechanism of formate tolerance. The elevated tolerance to high concentrations of formate was attributed to alterations in metabolic pathways, including those involved in the transport of formate, methanol oxidation, formate oxidation, and assimilation pathways. The FT3 strain, which exhibited a significantly increased synthesis of reducing equivalents in the presence of formate and enhanced 3-HP tolerance, was deemed an appropriate chassis for the production of reduced chemicals of 3-HP. A 3-HP titer of 2.47 g/L was attained through fed-batch fermentation by utilizing the combined carbon sources of methanol and formate.

## Data Availability

The whole-genome re-sequencing data and transcriptome data are deposited in NCBI. The accession numbers for the genome sequences of *Methylorubrum extorquens* FT3 deposited in NCBI are CP195989 and CP195990. The BioProject accession number for the RNASeq data is PRJNA1285123. The sequences of the plasmids generated in this study are listed in the supplemental material.

## SUPPLEMENTAL MATERIAL

Data Set S1 aem.02560-24-s0001.xlsx SNP and InDel mutations in FT3.

Data Set S2 aem.02560-24-s0002.xls Top 20 significantly up-regulated genes in *M. extorquens* FT3 in the presence of 30 mM formate.

Supplemental material aem.02560-24-s0003.docx Table S1, Fig. S1 to S15, and sequences of the plasmids generated in this study.

ASM does not own the copyrights to Supplemental Material that may be linked to, or accessed through, an article. The authors have granted ASM a non-exclusive, world-wide license to publish the Supplemental Material files. Please contact the corresponding author directly for reuse.
