# Supplementary material for: Adaptively evolved Methylorubrum extorquens with enhanced formate tolerance and its application in 3-hydroxypropionic acid production
Source: Appl Environ Microbiol. 2025 Aug 13;91(9):e02560-24. doi: 10.1128/aem.02560-24 (PMC12445348; doi:10.1128/aem.02560-24)
Supplement: Supplemental material — Table S1, Fig. S1 to S15, and sequences of the plasmids generated in this study. [file aem.02560-24-s0003.docx]

**Adaptively evolved *Methylorubrum extorquens* with enhanced formate tolerance and its application in 3-hydroxypropionic acid production**

Xuhua Mo^1#^, Yan Zhao^1#^, Lin Zhu^1#^, Changtai Zhang^1#^, Zengxin Ma^1^, Kai Bao^2^, Song Yang^1, 3*^

1. School of Life Sciences, Shandong Province Key Laboratory of Applied Mycology, and Qingdao International Center on Microbes Utilizing Biogas, Qingdao Agricultural University, Qingdao, Shandong Province, People’s Republic of China
2. School of Life Sciences, Hubei University, Wuhan 430062, Hubei Province, People’s Republic of China
3. Key Laboratory of Systems Bioengineering, Ministry of Education, Tianjin University, Tianjin, People’s Republic of China

^#^ # Xuhua Mo, Yan Zhao, Lin Zhu and Changtai Zhang contributed equally to this work.

*Corresponding author: Song Yang, SongYang@qau.edu.cn or [yangsong1209@163.com](mailto:yangsong1209@163.com)

The lists of supplementary materials

| **Table S1.** The primers used in this study. |
| --- |
| **Figure S1**. Growth of *M. extorquens* AM1 on media containing varying concentrations of formate as the sole carbon source. |
| **Figure S2**. Growth of the 12 evolved strains (FT1 to FT12) isolated at the 300^th^ generation, cultivated with 30 mM methanol and 90 mM formate. |
| **Figure S3**. Growth of evolved strain *M. extorquens* FT3 and wild type strain *M. extorquens* AM1 *ΔcelAB* on the media containing 120 mM methanol as the sole carbon source. |
| **Figure S4**. The C_30_ carotenoids concentration ratio of *M. extorquens* FT3 cultivated with Hypho medium containing 90 mM methanol (M) and 30 mM formate (F), 60 mM methanol and 60 mM formate, and 120 mM methanol, respectively. |
| **Figure S5**. Analyses the tolerance of the *M. extorquens* AM1 *ΔcelAB* strain and the FT3 strain to other organic acids and alcohols. |
| **Figure S6**. The scheme of analyses of 13C-labled amino acids in the FT3 strain. |
| **Figure S7**. Transcriptome analysis of the *M. extorquens* AM1*ΔcelAB* strain and the FT3 strain cultivated with Hypho medium supplemented with 150 mM methanol. |
| **Figure S8**. Transcriptome analysis of the FT3 strain cultivated with Hypho medium supplemented with either 150 mM methanol or 120 mM methanol and 30 mM formate. |
| **Figure S9.** Analysis of pH change in the culture by addition of 10 mM formate. |
| **Figure S10.** Growth curves of *M. extorquens* AM1 and the engineered strain *M. extorquens* AM1-MxaF* harboring C_159_ SNP mutation in the promoter *P_maxF_* in the presence of different formate concentrations. |
| **Figure S11**. Investigating the formaldehyde tolerance of the *M. extorquens* AM1 *ΔcelAB* strain and the FT3 strain. |
| **Figure S12**. Comparing the promoter strength of *fdh2CBAD* operon derived from *M. extorquens* AM1 and the FT3 strain harboring a G_136_ to T_136_ mutation. |
| **Figure S13**. Growth curves of the FT3 strain cultivated in Hypho medium containing 120 mM methanol, employing flasks with two different sealing methods. |
| **Figure S14**. The gene organization of *META1_3029*, *META1_3028* and *META1_3027* on the chromosome of *M. extorquens* AM1. |
| **Figure S15**. Comparison of the transcriptomic levels of genes involved in the synthesis of C_30_ terpenoid and hopanoid. The fold change of gene transcription in the FT3 strain compared to the parental *M. extorquens* AM1 *ΔcelAB* strain. |
| **Appendixes:** The sequences of the plasmids constructed in this study. |

**Table S1**. The primers used in this study.

| **Primer** **name** | **Sequence (5' to 3')** | **Usage** |
| --- | --- | --- |
| pCM80-Apr-F | tggttcactcgaacgacgtcggttcatgtgcagctccat | Primers for generating pCM80-Apr plasmid |
| pCM80-Apr-R | tcgccatcagtgatatctcagccaatcgactggcgagc | Primers for generating pCM80-Apr plasmid |
| pCM80-Apr-mcr-F | tacgccaagctttctagattcccgcttggtcgggccgc | Primers for generating pCM80-Apr-mcr plasmid |
| pCM80-Apr-mcr-R | cggccagtgaattcgagctcttacacggtaatcgcccg | Primers for generating pCM80-Apr-mcr plasmid |
| *fdh1*-F | gaaacgctggtggggtatgctc | Primers to verify the SNP mutation in *Fdh1* |
| *fdh1*-R | cgaactacttccgcgccaagcag | Primers to verify the the SNP mutation in *Fdh1* |
| *fdh2*-F | tgaagcgttacgcacagttccg | Primers to verify the SNP mutation in *Fdh2* |
| *fdh2*-R | tcagacccgcatccgcctc | Primers to verify the SNP mutation in *Fdh2* |
| *fdh3*-F | agtcgatgagcgagccggag | Primers to verify the SNP mutation in *Fdh3* |
| *fdh3*-R | ctccggctgcttcacgaatg | Primers to verify the SNP mutation in *Fdh3* |
| *fdh4*-F | cgcatggtctctttcagtgggctc | Primers to verify the SNP mutation in *Fdh4* |
| *fdh4*-R | caccctgattcgaaccgagctg | Primers to verify the SNP mutation in *Fdh4* |
| *P_mxaF_*-F | cccgcttggtcgggccgctt | Primers used to amplify the promoter *P_mxaF_* |
| *P_mxaF_*-R | gcggtatctctcagacgtt | Primers used to amplify the promoter *P_mxaF_* |
| *P_fdh2_*-F | cccgatggcctcgaagaaaat | Primers used to amplify the promoter *P_fdh2_* |
| *P_fdh2_*-R | cgtttcctgcccaacttg | Primers used to amplify the promoter *P_fdh2_* |
| pCM80-0287-F | accatgattacgccaagcttttgggctgcgacgagacgac | Amplification of *META1_0287* for overexpression |
| pCM80-0287-R | agctcggtacccggggatcctcaggcgccgacgcgattg | Amplification of *META1_0287* for overexpression |
| pCM80-2965-F | accatgattacgccaagcttgtgactggaacgacatctttg | Amplification of *META1_2965* for overexpression |
| pCM80-2965-R | agctcggtacccggggatcctcagcagccgacacaaatg | Amplification of *META1_2965* for overexpression |
| pCM80-3029-F | accatgattacgccaagcttatgttcgatatgaagcggattgtg | Amplification of *META1_3029* for overexpression |
| pCM80-3029-R | agctcggtacccggggatccttagcgcgcgcggcgcggcgcggt | Amplification of *META1_3029* for overexpression |
| pCM80-1418-F | accatgattacgccaagcttatgtttcctcaggttctgaccgac | Amplification of *META1_1418* for overexpression |
| pCM80-1418-R | agctcggtacccggggatccttaagccgcggtgcagccga | Amplification of *META1_1418* for overexpression |
| pCM80-3027-F | accatgattacgccaagcttatgcgcgcctccctcgccc | Amplification of *META1_3027* for overexpression |
| pCM80-3027-R | agctcggtacccggggatcctcagtccttcggcgcggcgtga | Amplification of *META1_3027* for overexpression |
| pCM80-3028-F | accatgattacgccaagcttgtggacggcatcatccaaggcctg | Amplification of *META1_3028* for overexpression |
| pCM80-3028-R | agctcggtacccggggatccctactccgctgcgatagcgtg | Amplification of *META1_3028* for overexpression |
| pCM80-1260-F | accatgattacgccaagcttatggtcgatccggttacggaaac | Amplification of *META1_1260* for overexpression |
| pCM80-1260-R | agctcggtacccggggatccttacttccggagcgcggca | Amplification of *META1_1260* for overexpression |
| pCM80-1261-F | tatgaccatgattacgccaagcttatggtcgatccggttacggaaac | Amplification of *META1_1261* for overexpression |
| pCM80-1261-R | agctcggtacccggggatccttacttccggagcgcggca | Amplification of *META1_1261* |
| pCM80-3028-3027-F | accatgattacgccaagcttgtggacggcatcatccaaggcctg | Amplification of the *META1_3028* and *META1_3027* operon for overexpression |
| pCM80-3028-3027-R | agctcggtacccggggatcctcagtccttcggcgcggcgtga | Amplification of the *META1_3028* and *META1_3027* operon for overexpression |
| RBS-3029 | ataacaaccgttggggaggcatcccatgttcgatatgaagcggat | Amplification of *META1_3029* for overexpression with the operon *META1_3027* and *META1_3028* |
| pCM80-3028-3027-RBS-3029-F | aaggactgaggatccccgggtaccataacaaccgttggggaggca | Amplification of *META1_3027* and *META1_3028* for overexpression with *META1_3029* |
| pCM80-3027-3028-3029-R | aacgacggccagtgaattcgagctcttagcgcgcgcggcgcgcggcgcggt | Amplification of *META1_3027* and *META1_3028* for overexpression with *META1_3029* |
| pCM433-MxaF*-F | aagtgccacctgacgtctagatctgatcgtcatcgtccacttgttg | Amplification of *mxaF** for homologous exchange of *mxaF* in AM1 |
| pCM433-MxaF*-R | tcggctggatcctctagtgagctccctcgtcgaacagtcccgaac | Amplification of *mxaF** for homologous exchange of *mxaF* in AM1 |
| pCM433-*P_mxaF_/P_3028-3027_*-UP-F | aagtgccacctgacgtctagatcttcggtcagcacgtggatctg | Primers for P_mxaF_ homologous exchange of *META1_*3028-*META1_*3027 promoter |
| pCM433-*P_mxaF_/P_3028-3027_*-UP-R | aaacgtctgagagataccgcgtggacggcatcatccaag | Primers for P_mxaF_ homologous exchange of *META1_*3028-*META1_*3027 promoter |
| pCM433-*P_mxaF_/P_3028-3027_*-PmxaF-F | ccttggatgatgccgtccacgcggtatctctcagacgtt | Primers for P_mxaF_ homologous exchange of *META1_*3028-*META1_*3027 promoter |
| pCM433-*P_mxaF_/P_3028-3027_-*PmxaF-R | cgccgcgccgcgcgcgctaacccgcttggtcgggccgct | Primers for P_mxaF_ homologous exchange of *META1_*3028-*META1_*3027 promoter |
| pCM433-*P_mxaF_/P_3028-3027_*-DOWN-F | aagcggcccgaccaagcgggttagcgcgcgcggcgcggcgcggt | Primers for P_mxaF_ homologous exchange of *META1_*3028-*META1_*3027 promoter |
| pCM433-*P_mxaF_/P_3028-3027_*-DOWN-R | tcggctggatcctctagtgagctcaatgtggggaccaccgaaag | Primers for P_mxaF_ homologous exchange of *META1_*3028-*META1_*3027 promoter |
| cel-up-F | acacaggaaacagctatgacatgattacgaattctcccgggaatctctgtattcttgtc | Amplification of upstream sequence of *celAB* for homologous exchange |
| cel-up-R | acttgtagagatcgaaggcgtcctccgatttccggatgcgttcacgacgc | Amplification of upstream sequence of *celAB* for homologous exchange |
| cel-down-F | gcgtcgtgaacgcatccggaaatcggaggacgccttcgatctctacaagt | Amplification of downstream sequence of *celAB* for homologous exchange |
| cel-down-R | acgacgttgtaaaacgacggccagtgccaagcttgtagctgccgggcgcgaccggctcg | Amplification of downstream sequence of *celAB* for homologous exchange |
| cel-test-F | gcaggcccgagcggcctgaatccgc | Used for verify the AM1 *ΔcelAB* strain |
| cel-test-R | cctgctggtcctgccagagcgtgcc | Used for verify the AM1 *ΔcelAB* strain |


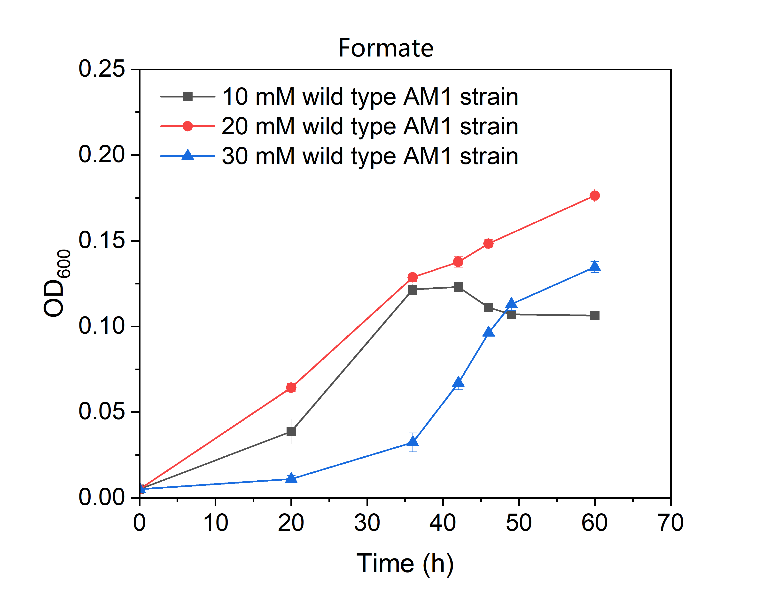


**Figure S1**. Growth of *M. extorquens* AM1 on media containing varying concentrations of formate as the sole carbon source.





**Figure S2**. Growth of the 12 evolved strains (FT1 to FT12) isolated at the 300^th^ generation, cultivated with 30 mM methanol and 90 mM formate.


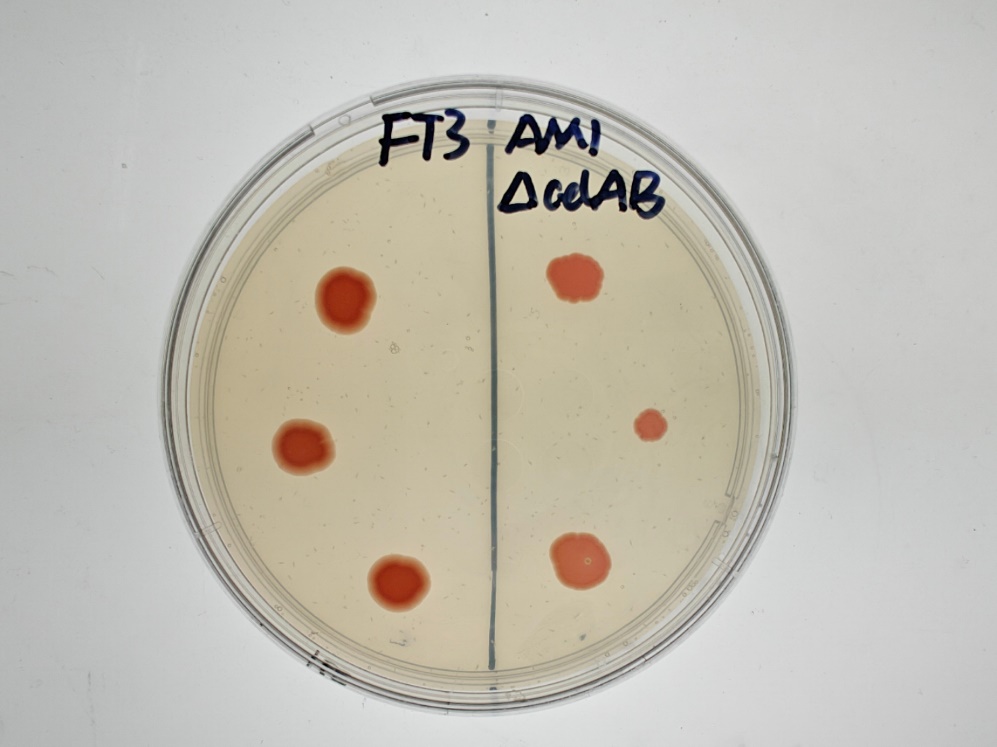


**Figure S3**. Growth of evolved strain *M. extorquens* FT3 and wild type strain *M. extorquens* AM1 *ΔcelAB* on the media containing 120 mM methanol as the sole carbon source.


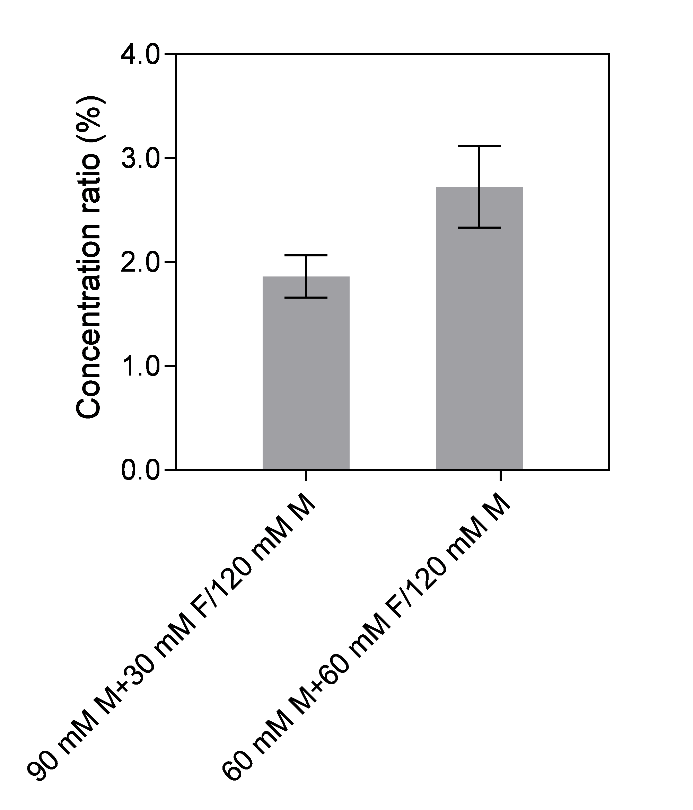


**Figure S4**. The C_30_ carotenoids concentration ratio of *M. extorquens* FT3 cultivated with Hypho medium containing 90 mM methanol (M) and 30 mM formate (F), 60 mM methanol and 60 mM formate, and 120 mM methanol, respectively. The data were presented as the average of three replicates.


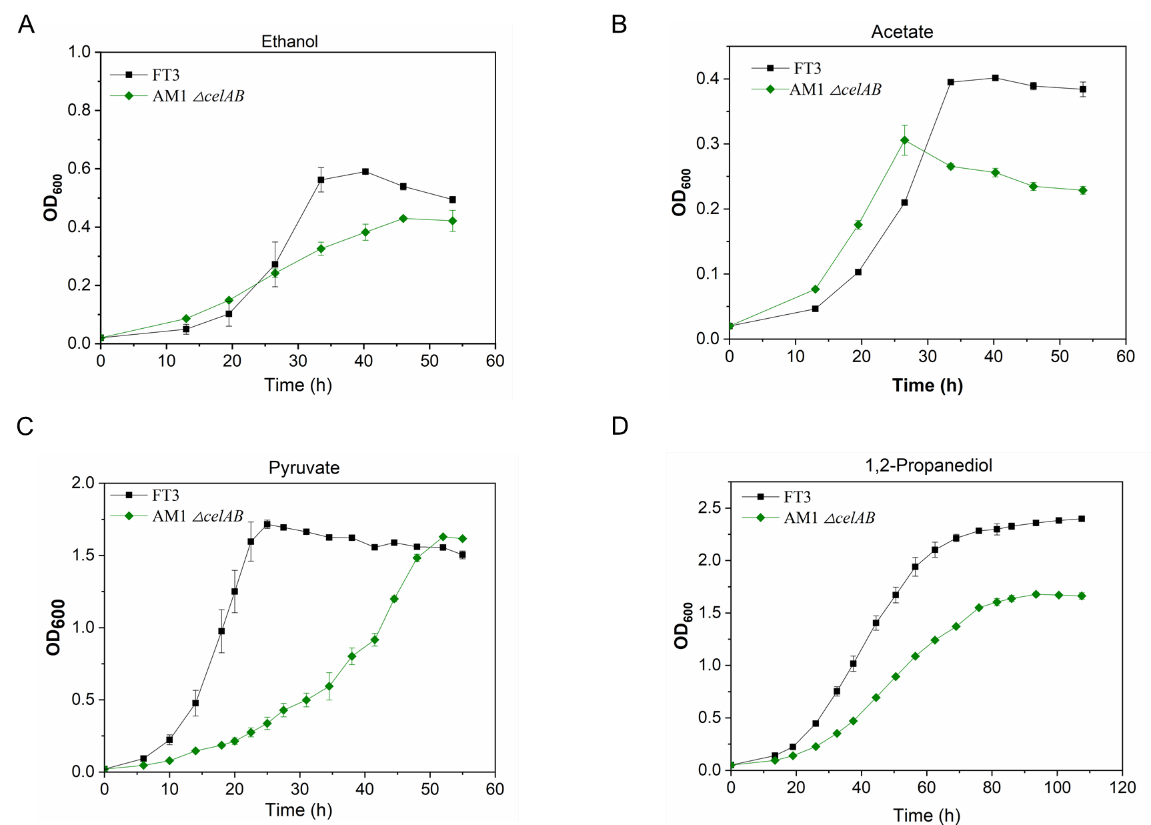


**Figure S5**. Analyses the tolerance of the *M. extorquens* AM1 *ΔcelAB* strain and the FT3 strain to other organic acids and alcohols. (A) Ethanol (34 mM), (B) acetate (5 mM), (C) pyruvate (36 mM), and (D) 1,2-propanediol (68 mM).


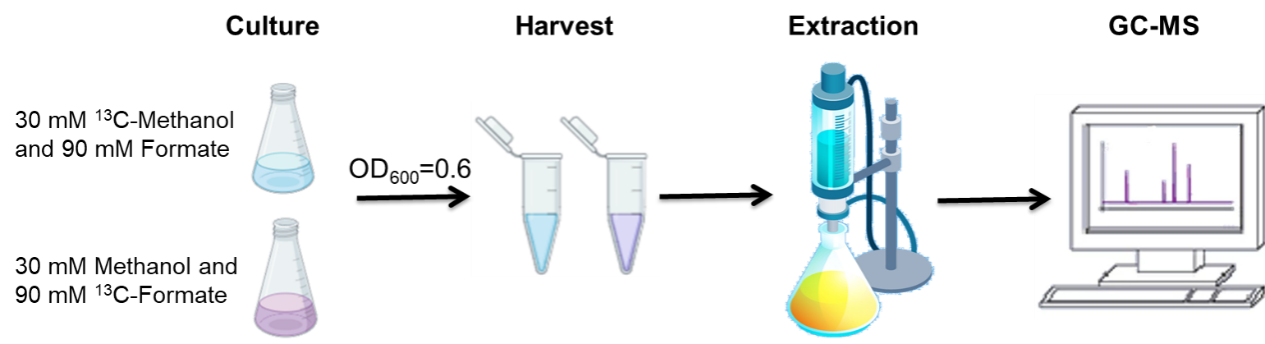


**Figure S6**. The scheme of analyses of ^13^C-labled amino acids in the FT3 strain cultivated with (i) 30 mM ^13^C-methanol and 90 mM ^12^C-formate, or (ii) 30 mM ^12^C-methanol and 90 mM ^13^C-formate.

**Figure S7**. Transcriptome analysis of the *M. extorquens* AM1*ΔcelAB* strain and the FT3 strain cultivated with Hypho medium supplemented with 150 mM methanol. The volcano plot of differential transcriptional levels as determined by RNA sequencing (*n* = 3), the pearson correlation is 0.8461.

**Figure S8**. Transcriptome analysis of the FT3 strain cultivated with Hypho medium supplemented with either 150 mM methanol or 120 mM methanol and 30 mM formate. The volcano plot of differential transcript levels as determined by RNA sequencing (*n* = 3), the pearson correlation is 0.9757.


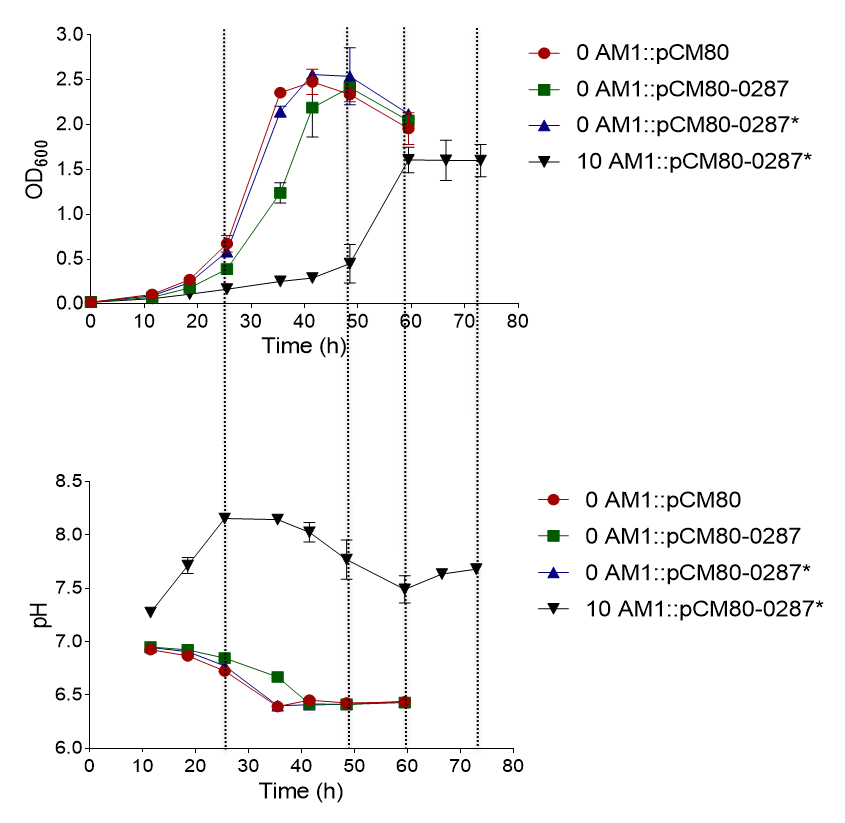


**Figure S9.** Analysis of pH change in the culture by addition of 10 mM formate.


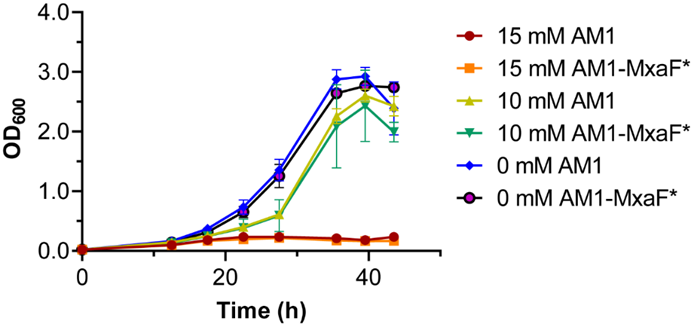


**Figure S10.** Growth curves of *M. extorquens* AM1 and the engineered strain *M. extorquens* AM1-MxaF* harboring C_159_ SNP mutation in the promoter *P_maxF_* in the presence of different formate concentrations.


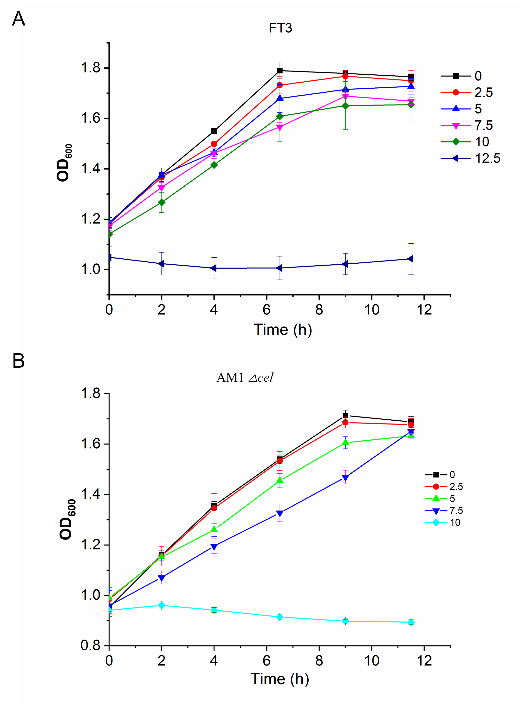


**Figure S11**. Investigating the formaldehyde tolerance of the *M. extorquens* AM1 *ΔcelAB* strain and the FT3 strain. (A) The growth curves of the FT3 strain cultivated with 120 mM methanol and 0 mM, 2.5 mM, 5 mM, 7.5 mM, 10 mM, and 12.5 mM formaldehyde, respectively. (B) The growth curves of the *M. extorquens* AM1 *ΔcelAB* strain cultivated with 120 mM methanol and 0 mM, 2.5 mM, 5 mM, 7.5 mM, and 10 mM formaldehyde, respectively.


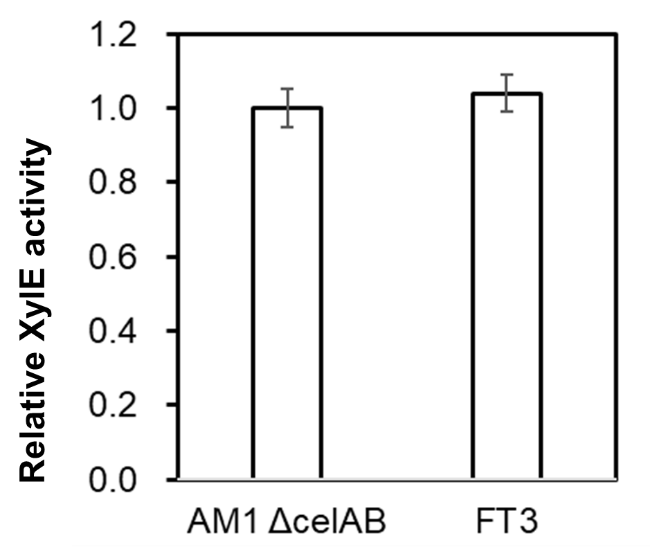


**Figure S12**. Comparing the promoter strength of *fdh2CBAD* operon derived from *M. extorquens* AM1 and the FT3 strain harboring a G_136_ to T_136_ mutation.





**Figure S13**. Growth curves of the FT3 strain cultivated in Hypho medium containing 120 mM methanol, employing flasks with two different sealing methods.


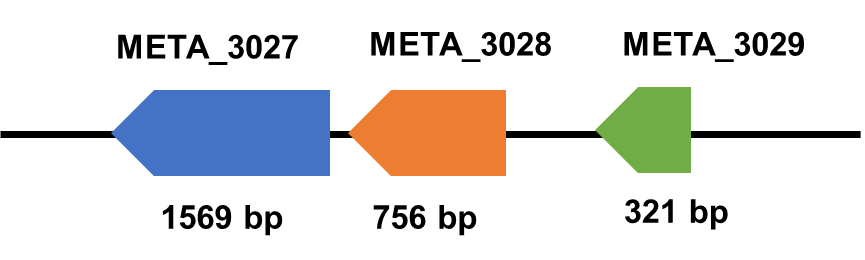


**Figure S14**. The gene organization of *META1_3029*, *META1_3028* and *META1_3027* on the chromosome of *M. extorquens* AM1.


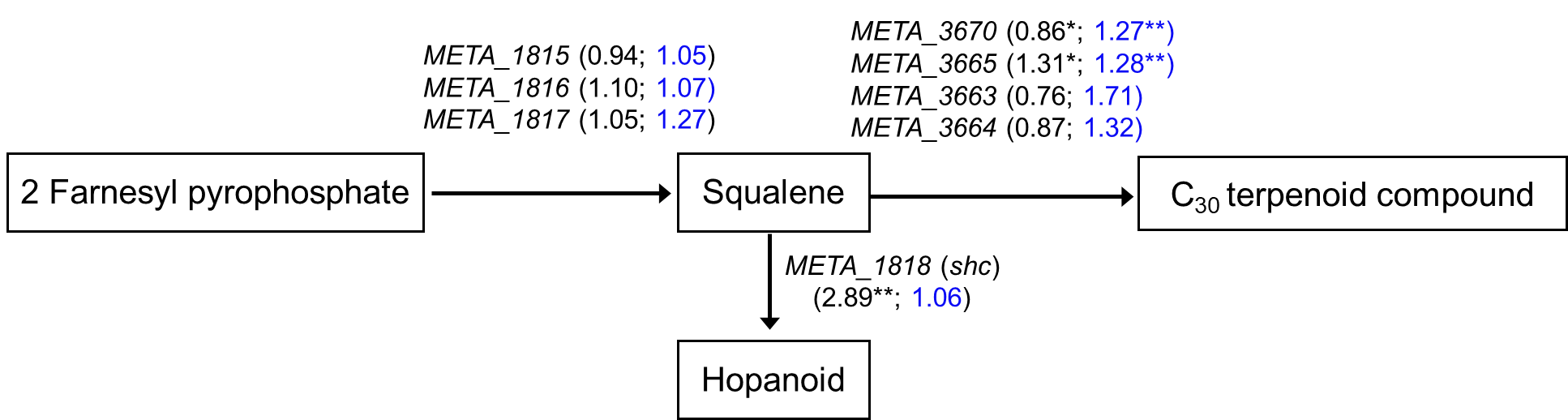


**Figure S15**. Comparison of the transcriptomic levels of genes involved in the synthesis of C_30_ terpenoid and hopanoid. The fold change of gene transcription in the FT3 strain compared to the parental *M. extorquens* AM1 *ΔcelAB* strain. The data shown in black indicate the fold change of the strains cultivated with 150 mM methanol. The data shown in blue indicate the fold change of the strains cultivated with 120 mM methanol and 30 mM formate. The data were presented as the average of three replicates, with standard deviations indicated as error bars. Significant differentially expressed genes were defined as having a (FDR) *p** < 0.05 and *p*** < 0.01.

The list of plasmids constructed in this study

| **Plasmids** | **Description** |
| --- | --- |
| pCM80-Apr | Vector used for gene expression in *M. extorquens* FT3; promoter, *P_mxaF_*; antibiotics, Apr^R^ |
| pCM80-Apr-mcr | pCM80-Apr containing the operon *P_mxaF_*::*mcr*-*P_meta1_*__3616_::*mcr*_550–1219_ from pYM07 for the synthesis of 3-HP |
| pCM130-*P_mxaF*_* | Plasmid carrying the mutated promoter *P_mxaF*_* to drive *xylE* |
| pCM130-*P_mxaF_* | Plasmid carrying the promoter *P_mxaF_* to drive *xylE* |
| pCM130-*P_fdh2*_* | Plasmid carrying the mutated promoter *P_fdh2*_* to drive *xylE* |
| pCM130-*P_fdh2_* | Plasmid carrying the promoter *P_fdh2_* to drive *xylE* |
| pCM80-0287 | Plasmid overexpressing the gene *META1_0287* |
| pCM80-0287* | Plasmid overexpressing the mutated gene *META1_0287** |
| pCM80-2965 | Plasmid overexpressing the gene *META1_2965* |
| pCM80-3029 | Plasmid overexpressing the gene *META1_3029* |
| pCM80-1261 | Plasmid overexpressing the gene *META1_1261* |
| pCM80-1418 | Plasmid overexpressing the gene *META1_1418* |
| pCM80-3027 | Plasmid overexpressing the gene *META1_3027* |
| pCM80-3028 | Plasmid overexpressing the gene *META1_3028* |
| pCM80-1261-1260 | Plasmid overexpressing the genes *META1_1261* and *META1_1260* |
| pCM80-3028-3027 | Plasmid overexpressing the genes *META1_3028* and *META1_3027* |
| pCM80-3028-3027-3029 | Plasmid overexpressing the genes *META1_3028*, *META1_3027* and *META1_3029* |
| pCM433-MxaF* | Plasmid for homologous exchange of *P_mxaF_* promoter of MxaF with *P_mxaF_** |
| pCM433-*P_mxaF_-P_3028-3027_* | Plasmid for homologous exchange of native promoter of the operon *META1_*3028-*META1_*3027 with *P_mxaF_* promoter |

**The sequences of plasmids constructed in this study**

**pCM80-Apr, the tetracycline resistance gene *tetA* is replaced by apramycin resistance gene, which is highlighted in red.**

5´-

gaccctttccgacgctcaccgggctggttgccctcgccgctgggctggcggccgtctatggccctgcaaacgcgccagaaacgccgtcgaagccgtgtgcgagacaccgcggccgccggcgttgtggatacctcgcggaaaacttggccctcactgacagatgaggggcggacgttgacacttgaggggccgactcacccggcgcggcgttgacagatgaggggcaggctcgatttcggccggcgacgtggagctggccagcctcgcaaatcggcgaaaacgcctgattttacgcgagtttcccacagatgatgtggacaagcctggggataagtgccctgcggtattgacacttgaggggcgcgactactgacagatgaggggcgcgatccttgacacttgaggggcagagtgctgacagatgaggggcgcacctattgacatttgaggggctgtccacaggcagaaaatccagcatttgcaagggtttccgcccgtttttcggccaccgctaacctgtcttttaacctgcttttaaaccaatatttataaaccttgtttttaaccagggctgcgccctgtgcgcgtgaccgcgcacgccgaaggggggtgcccccccttctcgaaccctcccggcccgctaacgcgggcctcccatccccccaggggctgcgcccctcggccgcgaacggcctcaccccaaaaatggcagccaagctgaccacttctgcgctcggcccttccggctggctggtttattgctgataaatctggagccggtgagcgtgggtctcgcggtatcattgcagcactggggccagatggtaagccctcccgtatcgtagttatctacacgacggggagtcaggcaactatggatgaacgaaatagacagatcgctgagataggtgcctcactgattaagcattggtaactgtcagaccaagtttactcatatatactttagattgatttaaaacttcatttttaatttaaaaggatctaggtgaagatcctttttgataatctcatgaccaaaatcccttaacgtgagttttcgttccactgagcgtcagaccccgtagaaaagatcaaaggatcttcttgagatcctttttttctgcgcgtaatctgctgcttgcaaacaaaaaaaccaccgctaccagcggtggtttgtttgccggatcaagagctaccaactctttttccgaaggtaactggcttcagcagagcgcagataccaaatactgtccttctagtgtagccgtagttaggccaccacttcaagaactctgtagcaccgcctacatacctcgctctgctaatcctgttaccagtggctgctgccagtggcgataagtcgtgtcttaccgggttggactcaagacgatagttaccggataaggcgcagcggtcgggctgaacggggggttcgtgcacacagcccagcttggagcgaacgacctacaccgaactgagatacctacagcgtgagctatgagaaagcgccacgcttcccgaagggagaaaggcggacaggtatccggtaagcggcagggtcggaacaggagagcgcacgagggagcttccagggggaaacgcctggtatctttatagtcctgtcgggtttcgccacctctgacttgagcgtcgatttttgtgatgctcgtcaggggggcggagcctatggaaaaacgccagcaacgcggcctttttacggttcctggccttttgctggccttttgctcacatgttctttcctgcgttatcccctgattctgtggataaccgtattaccgcctttgagtgagctgataccgctcgccgcagccgaacgaccgagcgcagcgagtcagtgagcgaggaagcggaagagcgcccaatacgcaaaccgcctctccccgcgcgttggccgattcattaatgcagctggcacgacaggtttcccgactggaaagcgggcagtgagcgcaacgcaattaatgtgagttagctcactcattaggcaccccaggctttacactttatgcttccggctcgtatgttgtgtggaattgtgagcggataacaatttcacacaggaaacagctatgaccatgattacgccaagctagcttcccgcttggtcgggccgcttcgcgagggcccgttgacgacaacggtgcgatgggtcccggccccggtcaagacgatgccaatacgttgcgacactacgccttggcacttttagaattgccttatcgtcctgataagaaatgtccgaccagctaaagacatcgcgtccaatcaaagcctagaaaatataggcgaagggacgctaataagtctttcataagaccgcgcaaatctaaaaatatccttagattcacgatgcggcacttcggatgacttccgagcgagcctggaacctcagaaaaacgtctgagagataccgcggatctcacacaggaaacagctatgaccatgattacgccaagcttctcgagctcgagggatcctctagaatggcagatctccatcaccatcatcaccatcacggtaccgagctcgaattcactggccgtcgttttacaacgtcgtgactgggaaaaccctggcgttacccaacttaatcgccttgcagcacatccccctttcgccagctggcgtaatagcgaagaggcccgcaccgatcgcccttcccaacagttgcgcagcctgaatggcgaatggcgcctgatgcggtattttctccttacgcatctgtgcggtatttcacaccgcatatggtgcactctcagtacaatctgctctgatgccgcatagttaagccagccccgacacccgccaacacccgctgacgcgccctgacgggcttgtctgctcccggcatccgcttacagacaagctgtgaccgtctccgggagctgcatgtgtcagaggttttcaccgtcatcaccgaaacgcgcgagacgaaagggcctcgtgatacgcctatttttataggttaatgtcatgataataatggtttcttagcaccctttctcggtccttcaacgttcctgacaacgagcctccttttcgccaatccatcgacaatcaccgcgagtccctgctcgaacgctgcgtccggaccggcttcgtcgaaggcgtctatcgcggcccgcaacagcggcgagagcggagcctgttcaacggtgccgccgcgctcgccggcatcgctgtcgccggcctgctcctcaagcacggccccaacagtgaagtagctgattgtcatcagcgcattgacggcgtccccggccgaaaaacccgcctcgcagaggaagcgaagctgcgcgtcggccgtttccatctgcggtgcgcccggtcgcgtgccggcatggatgcgcgcgccatcgcggtaggcgagcagcgcctgcctgaagctgcgggcattcccgatcagaaatgagcgccagtcgtcgtcggctctcggcaccgaatgcgtatgattctccgccagcatggcttcggccagtgcgtcgagcagcgcccgcttgttcctgaagtgccagtaaagcgccggctgctgaacccccaaccgttccgccagtttgcgtgtcgtcagaccgtctacgccgacctcgttcaacaggtccagggcggcacggatcactgtattcggctgcaactttgtcatgattgacactttatcactgataaacataatatgtccaccaacttatcagtgataaagaatccgcgcgttcaatcggaccagcggaggctggtccggaggccagacATGTCATCAGCGGTGGAGTGCAATGTCGTGCAATACGAATGGCGAAAAGCCGAGCTCATCGGTCAGCTTCTCAACCTTGGGGTTACCCCCGGCGGTGTGCTGCTGGTCCACAGCTCCTTCCGTAGCGTCCGGCCCCTCGAAGATGGGCCACTTGGACTGATCGAGGCCCTGCGTGCTGCGCTGGGTCCGGGAGGGACGCTCGTCATGCCCTCGTGGTCAGGTCTGGACGACGAGCCGTTCGATCCTGCCACGTCGCCCGTTACACCGGACCTTGGAGTTGTCTCTGACACATTCTGGCGCCTGCCAAATGTAAAGCGCAGCGCCCATCCATTTGCCTTTGCGGCAGCGGGGCCACAGGCAGAGCAGATCATCTCTGATCCATTGCCCCTGCCACCTCACTCGCCTGCAAGCCCGGTCGCCCGTGTCCATGAACTCGATGGGCAGGTACTTCTCCTCGGCGTGGGACACGATGCCAACACGACGCTGCATCTTGCCGAGTTGATGGCAAAGGTTCCCTATGGGGTGCCGAGACACTGCACCATTCTTCAGGATGGCAAGTTGGTACGCGTCGATTATCTCGAGAATGACCACTGCTGTGAGCGCTTTGCCTTGGCGGACAGGTGGCTCAAGGAGAAGAGCCTTCAGAAGGAAGGTCCAGTCGGTCATGCCTTTGCTCGGTTGATCCGCTCCCGCGACATTGTGGCGACAGCCCTGGGTCAACTGGGCCGAGATCCGTTGATCTTCCTGCATCCGCCAGAGGCGGGATGCGAAGAATGCGATGCCGCTCGCCAGTCGATTGGCTGAtcgtggaaacgataggcctatgccatgcgggtcaaggcgacttccggcaagctatacgcgccctagaattgtcaattttaatcctctgtttatcggcagttcgtagagcgcgccgtgcgtcccgagcgatactgagcgaagcaagtgcgtcgagcagtgcccgcttgttcctgaaatgccagtaaagcgctggctgctgaacccccagccggaactgaccccacaaggccctagcgtttgcaatgcaccaggtcatcattgacccaggcgtgttccaccaggccgctgcctcgcaactcttcgcaggcttcgccgacctgctcgcgccacttcttcacgcgggtggaatccgatccgcacatgaggcggaaggtttccagcttgagcgggtacggctcccggtgcgagctgaaatagtcgaacatccgtcgggccgtcggcgacagcttgcggtacttctcccatatgaatttcgtgtagtggtcgccagcaaacagcacgacgatttcctcgtcgatcaggacctggcaacgggacgttttcttgccacggtccaggacgcggaagcggtgcagcagcgacaccgattccaggtgcccaacgcggtcggacgtgaagcccatcgccgtcgcctgtaggcgcgacaggcattcctcggccttcgtgtaataccggccattgatcgaccagcccaggtcctggcaaagctcgtagaacgtgaaggtgatcggctcgccgataggggtgcgcttcgcgtactccaacacctgctgccacaccagttcgtcatcgtcggcccgcagctcgacgccggtgtaggtgatcttcacgtccttgttgacgtggaaaatgaccttgttttgcagcgcctcgcgcgggattttcttgttgcgcgtggtgaacagggcagagcgggccgtgtcgtttggcatcgctcgcatcgtgtccggccacggcgcaatatcgaacaaggaaagctgcatttccttgatctgctgcttcgtgtgtttcagcaacgcggcctgcttggcctcgctgacctgttttgccaggtcctcgccggcggtttttcgcttcttggtcgtcatagttcctcgcgtgtcgatggtcatcgacttcgccaaacctgccgcctcctgttcgagacgacgcgaacgctccacggcggccgatggcgcgggcagggcagggggagccagttgcacgctgtcgcgctcgatcttggccgtagcttgctggaccatcgagccgacggactggaaggtttcgcggggcgcacgcatgacggtgcggcttgcgatggtttcggcatcctcggcggaaaaccccgcgtcgatcagttcttgcctgtatgccttccggtcaaacgtccgattcattcaccctccttgcgggattgccccgactcacgccggggcaatgtgcccttattcctgatttgacccgcctggtgccttggtgtccagataatccaccttatcggcaatgaagtcggtcccgtagaccgtctggccgtccttctcgtacttggtattccgaatcttgccctgcacgaataccagctccgcgaagtcgctcttcttgatggagcgcatggggacgtgcttggcaatcacgcgcaccccccggccgttttagcggctaaaaaagtcatggctctgccctcgggcggaccacgcccatcatgaccttgccaagctcgtcctgcttctcttcgatcttcgccagcagggcgaggatcgtggcatcaccgaaccgcgccgtgcgcgggtcgtcggtgagccagagtttcagcaggccgcccaggcggcccaggtcgccattgatgcgggccagctcgcggacgtgctcatagtccacgacgcccgtgattttgtagccctggccgacggccagcaggtaggcctacaggctcatgccggccgccgccgccttttcctcaatcgctcttcgttcgtctggaaggcagtacaccttgataggtgggctgcccttcctggttggcttggtttcatcagccatccgcttgccctcatctgttacgccggcggtagccggccagcctcgcagagcaggattcccgttgagcaccgccaggtgcgaataagggacagtgaagaaggaacacccgctcgcgggtgggcctacttcacctatcctgcccggctgacgccgttggatacaccaaggaaagtctacacgaaccctttggcaaaatcctgtatatcgtgcgaaaaaggatggatataccgaaaaaatcgctataatgaccccgaagcagggttatgcagcggaaaagatccgtc-3´

**pCM80-Apr-mcr, the operon *mcr*-*P_meta1_*__3616_::*mcr*_550–1219_ is highlighted in red**

5´-

gaccctttccgacgctcaccgggctggttgccctcgccgctgggctggcggccgtctatggccctgcaaacgcgccagaaacgccgtcgaagccgtgtgcgagacaccgcggccgccggcgttgtggatacctcgcggaaaacttggccctcactgacagatgaggggcggacgttgacacttgaggggccgactcacccggcgcggcgttgacagatgaggggcaggctcgatttcggccggcgacgtggagctggccagcctcgcaaatcggcgaaaacgcctgattttacgcgagtttcccacagatgatgtggacaagcctggggataagtgccctgcggtattgacacttgaggggcgcgactactgacagatgaggggcgcgatccttgacacttgaggggcagagtgctgacagatgaggggcgcacctattgacatttgaggggctgtccacaggcagaaaatccagcatttgcaagggtttccgcccgtttttcggccaccgctaacctgtcttttaacctgcttttaaaccaatatttataaaccttgtttttaaccagggctgcgccctgtgcgcgtgaccgcgcacgccgaaggggggtgcccccccttctcgaaccctcccggcccgctaacgcgggcctcccatccccccaggggctgcgcccctcggccgcgaacggcctcaccccaaaaatggcagccaagctgaccacttctgcgctcggcccttccggctggctggtttattgctgataaatctggagccggtgagcgtgggtctcgcggtatcattgcagcactggggccagatggtaagccctcccgtatcgtagttatctacacgacggggagtcaggcaactatggatgaacgaaatagacagatcgctgagataggtgcctcactgattaagcattggtaactgtcagaccaagtttactcatatatactttagattgatttaaaacttcatttttaatttaaaaggatctaggtgaagatcctttttgataatctcatgaccaaaatcccttaacgtgagttttcgttccactgagcgtcagaccccgtagaaaagatcaaaggatcttcttgagatcctttttttctgcgcgtaatctgctgcttgcaaacaaaaaaaccaccgctaccagcggtggtttgtttgccggatcaagagctaccaactctttttccgaaggtaactggcttcagcagagcgcagataccaaatactgtccttctagtgtagccgtagttaggccaccacttcaagaactctgtagcaccgcctacatacctcgctctgctaatcctgttaccagtggctgctgccagtggcgataagtcgtgtcttaccgggttggactcaagacgatagttaccggataaggcgcagcggtcgggctgaacggggggttcgtgcacacagcccagcttggagcgaacgacctacaccgaactgagatacctacagcgtgagctatgagaaagcgccacgcttcccgaagggagaaaggcggacaggtatccggtaagcggcagggtcggaacaggagagcgcacgagggagcttccagggggaaacgcctggtatctttatagtcctgtcgggtttcgccacctctgacttgagcgtcgatttttgtgatgctcgtcaggggggcggagcctatggaaaaacgccagcaacgcggcctttttacggttcctggccttttgctggccttttgctcacatgttctttcctgcgttatcccctgattctgtggataaccgtattaccgcctttgagtgagctgataccgctcgccgcagccgaacgaccgagcgcagcgagtcagtgagcgaggaagcggaagagcgcccaatacgcaaaccgcctctccccgcgcgttggccgattcattaatgcagctggcacgacaggtttcccgactggaaagcgggcagtgagcgcaacgcaattaatgtgagttagctcactcattaggcaccccaggctttacactttatgcttccggctcgtatgttgtgtggaattgtgagcggataacaatttcacacaggaaacagctatgaccatgattacgccaagctagcttcccgcttggtcgggccgcttcgcgagggcccgttgacgacaacggtgcgatgggtcccggccccggtcaagacgatgccaatacgttgcgacactacgccttggcacttttagaattgccttatcgtcctgataagaaatgtccgaccagctaaagacatcgcgtccaatcaaagcctagaaaatataggcgaagggacgctaataagtctttcataagaccgcgcaaatctaaaaatatccttagattcacgatgcggcacttcggatgacttccgagcgagcctggaacctcagaaaaacgtctgagagataccgcggatctcacacaggaaacagctatgaccatgattacgccaagcttctcgagATGAGCGGAACAGGACGACTGGCAGGAAAGATTGCGTTAATTACCGGTGGCGCCGGCAATATCGGCAGTGAATTGACACGTCGCTTTCTCGCAGAGGGAGCGACGGTCATTATTAGTGGACGGAATCGGGCGAAGTTGACCGCACTGGCCGAACGGATGCAGGCAGAGGCAGGAGTGCCGGCAAAGCGCATCGATCTCGAAGTCATGGATGGGAGTGATCCGGTCGCGGTACGTGCCGGTATCGAAGCGATTGTGGCCCGTCACGGCCAGATCGACATTCTGGTCAACAATGCAGGAAGTGCCGGTGCCCAGCGTCGTCTGGCCGAGATTCCACTCACTGAAGCTGAATTAGGCCCTGGCGCCGAAGAGACGCTTCATGCCAGCATCGCCAATTTACTTGGTATGGGATGGCATCTGATGCGTATTGCGGCACCTCATATGCCGGTAGGAAGTGCGGTCATCAATGTCTCGACCATCTTTTCACGGGCTGAGTACTACGGGCGGATTCCGTATGTCACCCCTAAAGCTGCTCTTAATGCTCTATCTCAACTTGCTGCGCGTGAGTTAGGTGCACGTGGCATCCGCGTTAATACGATCTTTCCCGGCCCGATTGAAAGTGATCGCATCCGTACAGTGTTCCAGCGTATGGATCAGCTCAAGGGGCGGCCCGAAGGCGACACAGCGCACCATTTTTTGAACACCATGCGATTGTGTCGTGCCAACGACCAGGGCGCGCTTGAACGTCGGTTCCCCTCCGTCGGTGATGTGGCAGACGCCGCTGTCTTTCTGGCCAGTGCCGAATCCGCCGCTCTCTCCGGTGAGACGATTGAGGTTACGCACGGAATGGAGTTGCCGGCCTGCAGTGAGACCAGCCTGCTGGCCCGTACTGATCTGCGCACGATTGATGCCAGTGGCCGCACGACGCTCATCTGCGCCGGCGACCAGATTGAAGAGGTGATGGCGCTCACCGGTATGTTGCGTACCTGTGGGAGTGAAGTGATCATCGGCTTCCGTTCGGCTGCGGCGCTGGCCCAGTTCGAGCAGGCAGTCAATGAGAGTCGGCGGCTGGCCGGCGCAGACTTTACGCCTCCCATTGCCTTGCCACTCGATCCACGCGATCCGGCAACAATTGACGCTGTCTTCGATTGGGCCGGCGAGAATACCGGCGGGATTCATGCAGCGGTGATTCTGCCTGCTACCAGTCACGAACCGGCACCGTGCGTGATTGAGGTTGATGATGAGCGGGTGCTGAATTTTCTGGCCGATGAAATCACCGGGACAATTGTGATTGCCAGTCGCCTGGCCCGTTACTGGCAGTCGCAACGGCTTACCCCCGGCGCACGTGCGCGTGGGCCGCGTGTCATTTTTCTCTCGAACGGTGCCGATCAAAATGGGAATGTTTACGGACGCATTCAAAGTGCCGCTATCGGTCAGCTCATTCGTGTGTGGCGTCACGAGGCTGAACTTGACTATCAGCGTGCCAGCGCCGCCGGTGATCATGTGCTGCCGCCGGTATGGGCCAATCAGATTGTGCGCTTCGCTAACCGCAGCCTTGAAGGGTTAGAATTTGCCTGTGCCTGGACAGCTCAATTGCTCCATAGTCAACGCCATATCAATGAGATTACCCTCAACATCCCTGCCAACATTAGCGCCACCACCGGCGCACGCAGTGCATCGGTCGGATGGGCGGAAAGCCTGATCGGGTTGCATTTGGGGAAAGTTGCCTTGATTACCGGTGGCAGCGCCGGTATTGGTGGGCAGATCGGGCGCCTCCTGGCTTTGAGTGGCGCGCGCGTGATGCTGGCAGCCCGTGATCGGCATAAGCTCGAACAGATGCAGGCGATGATCCAATCTGAGCTGGCTGAGGTGGGGTATACCGATGTCGAAGATCGCGTCCACATTGCACCGGGCTGCGATGTGAGTAGCGAAGCGCAGCTTGCGGATCTTGTTGAACGTACCCTGTCAGCTTTTGGCACCGTCGATTATCTGATCAACAACGCCGGGATCGCCGGTGTCGAAGAGATGGTTATCGATATGCCAGTTGAGGGATGGCGCCATACCCTCTTCGCCAATCTGATCAGCAACTACTCGTTGATGCGCAAACTGGCGCCGTTGATGAAAAAACAGGGTAGCGGTTACATCCTTAACGTCTCATCATACTTTGGCGGTGAAAAAGATGCGGCCATTCCCTACCCCAACCGTGCCGATTACGCCGTCTCGAAGGCTGGTCAGCGGGCAATGGCCGAAGTCTTTGCGCGCTTCCTTGGCCCGGAGATACAGATCAATGCCATTGCGCCGGGTCCGGTCGAAGGTGATCGCTTGCGCGGTACCGGTGAACGTCCCGGCCTCTTTGCCCGTCGGGCGCGGCTGATTTTGGAGAACAAGCGGCTGAATGAGCTTCACGCTGCTCTTATCGCGGCTGCGCGCACCGATGAGCGATCTATGCACGAACTGGTTGAACTGCTCTTACCCAATGATGTGGCCGCACTAGAGCAGAATCCCGCAGCACCTACCGCGTTGCGTGAACTGGCACGACGTTTTCGCAGCGAAGGCGATCCGGCGGCATCATCAAGCAGTGCGCTGCTGAACCGTTCAATTGCCGCTAAATTGCTGGCTCGTTTGCATAATGGTGGCTATGTGTTGCCTGCCGACATCTTTGCAAACCTGCCAAACCCGCCCGATCCCTTCTTCACCCGAGCCCAGATTGATCGCGAGGCTCGCAAGGTTCGTGACGGCATCATGGGGATGCTCTACCTGCAACGGATGCCGACTGAGTTTGATGTCGCAATGGCCACCGTCTATTACCTTGCCGACCGCAATGTCAGTGGTGAGACATTCCACCCATCAGGTGGTTTGCGTTACGAACGCACCCCTACCGGTGGCGAACTCTTCGGCTTGCCCTCACCGGAACGGCTGGCGGAGCTGGTCGGAAGCACGGTCTATCTGATAGGTGAACATCTGACTGAACACCTTAACCTGCTTGCCCGTGCGTACCTCGAACGTTACGGGGCACGTCAGGTAGTGATGATTGTTGAGACAGAAACCGGGGCAGAGACAATGCGTCGCTTGCTCCACGATCACGTCGAGGCTGGTCGGCTGATGACTATTGTGGCCGGTGATCAGATCGAAGCCGCTATCGACCAGGCTATCACTCGCTACGGTCGCCCAGGGCCGGTCGTCTGTACCCCCTTCCGGCCACTGCCGACGGTACCACTGGTCGGGCGTAAAGACAGTGACTGGAGCACAGTGTTGAGTGAGGCTGAATTTGCCGAGTTGTGCGAACACCAGCTCACCCACCATTTCCGGGTAGCGCGCAAGATTGCCCTGAGTGATGGTGCCAGTCTCGCGCTGGTCACTCCCGAAACTACGGCTACCTCAACTACCGAGCAATTTGCTCTGGCTAACTTCATCAAAACGACCCTTCACGCTTTTACGGCTACGATTGGTGTCGAGAGCGAAAGAACTGCTCAGCGCATTCTGATCAATCAAGTCGATCTGACCCGGCGTGCGCGTGCCGAAGAGCCGCGTGATCCGCACGAGCGTCAACAAGAACTGGAACGTTTTATCGAGGCAGTCTTGCTGGTCACTGCACCACTCCCGCCTGAAGCCGATACCCGTTACGCCGGGCGGATTCATCGCGGACGGGCGATTACCGTGTAACTCGAGggatccCTCAAGGCACCGGCATCGCTTCTGCAGGCATGTCGGACCGCAGCCTGGACGCTCGGCGAGCCCCCCAGGATCGACCCGACGAAAGCGGTTTCCTCAGCCAAACCGGCCGCAAATCGAAATCTCAGGTAAACTTTCTGCCGTCAAACTTGCCTTAGGTTACGATAGCGGAATGCAACCGCCCTCGTAAAGCGCTCAACCGGCACGACGGCGAAAACTCAACCTCAAGTCAACAGAGTGCGGGTCAGGGCCGGCATCGGCCTTAACGGGCGGTTAACTATGTTAGGTGAAATCCGGTGTCTGGGCGGCCCCGGGATCACAGGACCGGCAGCCCGACGAAACCGAGAGCGGTGGGGTGGCCGGCTCTAGAATGGCAGATCTCCATCACCATCATCACCATCACAGCGCCACCACCGGCGCACGCAGTGCATCGGTCGGATGGGCGGAAAGCCTGATCGGGTTGCATTTGGGGAAAGTTGCCTTGATTACCGGTGGCAGCGCCGGTATTGGTGGGCAGATCGGGCGCCTCCTGGCTTTGAGTGGCGCGCGCGTGATGCTGGCAGCCCGTGATCGGCATAAGCTCGAACAGATGCAGGCGATGATCCAATCTGAGCTGGCTGAGGTGGGGTATACCGATGTCGAAGATCGCGTCCACATTGCACCGGGCTGCGATGTGAGTAGCGAAGCGCAGCTTGCGGATCTTGTTGAACGTACCCTGTCAGCTTTTGGCACCGTCGATTATCTGATCAACAACGCCGGGATCGCCGGTGTCGAAGAGATGGTTATCGATATGCCAGTTGAGGGATGGCGCCATACCCTCTTCGCCAATCTGATCAGCAACTACTCGTTGATGCGCAAACTGGCGCCGTTGATGAAAAAACAGGGTAGCGGTTACATCCTTAACGTCTCATCATACTTTGGCGGTGAAAAAGATGCGGCCATTCCCTACCCCAACCGTGCCGATTACGCCGTCTCGAAGGCTGGTCAGCGGGCAATGGCCGAAGTCTTTGCGCGCTTCCTTGGCCCGGAGATACAGATCAATGCCATTGCGCCGGGTCCGGTCGAAGGTGATCGCTTGCGCGGTACCGGTGAACGTCCCGGCCTCTTTGCCCGTCGGGCGCGGCTGATTTTGGAGAACAAGCGGCTGAATGAGCTTCACGCTGCTCTTATCGCGGCTGCGCGCACCGATGAGCGATCTATGCACGAACTGGTTGAACTGCTCTTACCCAATGATGTGGCCGCACTAGAGCAGAATCCCGCAGCACCTACCGCGTTGCGTGAACTGGCACGACGTTTTCGCAGCGAAGGCGATCCGGCGGCATCATCAAGCAGTGCGCTGCTGAACCGTTCAATTGCCGCTAAATTGCTGGCTCGTTTGCATAATGGTGGCTATGTGTTGCCTGCCGACATCTTTGCAAACCTGCCAAACCCGCCCGATCCCTTCTTCACCCGAGCCCAGATTGATCGCGAGGCTCGCAAGGTTCGTGACGGCATCATGGGGATGCTCTACCTGCAACGGATGCCGACTGAGTTTGATGTCGCAATGGCCACCGTCTATTACCTTGCCGACCGCAATGTCAGTGGTGAGACATTCCACCCATCAGGTGGTTTGCGTTACGAACGCACCCCTACCGGTGGCGAACTCTTCGGCTTGCCCTCACCGGAACGGCTGGCGGAGCTGGTCGGAAGCACGGTCTATCTGATAGGTGAACATCTGACTGAACACCTTAACCTGCTTGCCCGTGCGTACCTCGAACGTTACGGGGCACGTCAGGTAGTGATGATTGTTGAGACAGAAACCGGGGCAGAGACAATGCGTCGCTTGCTCCACGATCACGTCGAGGCTGGTCGGCTGATGACTATTGTGGCCGGTGATCAGATCGAAGCCGCTATCGACCAGGCTATCACTCGCTACGGTCGCCCAGGGCCGGTCGTCTGTACCCCCTTCCGGCCACTGCCGACGGTACCACTGGTCGGGCGTAAAGACAGTGACTGGAGCACAGTGTTGAGTGAGGCTGAATTTGCCGAGTTGTGCGAACACCAGCTCACCCACCATTTCCGGGTAGCGCGCAAGATTGCCCTGAGTGATGGTGCCAGTCTCGCGCTGGTCACTCCCGAAACTACGGCTACCTCAACTACCGAGCAATTTGCTCTGGCTAACTTCATCAAAACGACCCTTCACGCTTTTACGGCTACGATTGGTGTCGAGAGCGAAAGAACTGCTCAGCGCATTCTGATCAATCAAGTCGATCTGACCCGGCGTGCGCGTGCCGAAGAGCCGCGTGATCCGCACGAGCGTCAACAAGAACTGGAACGTTTTATCGAGGCAGTCTTGCTGGTCACTGCACCACTCCCGCCTGAAGCCGATACCCGTTACGCCGGGCGGATTCATCGCGGACGGGCGATTACCGTGTAAggtaccgagctcgaattcactggccgtcgttttacaacgtcgtgactgggaaaaccctggcgttacccaacttaatcgccttgcagcacatccccctttcgccagctggcgtaatagcgaagaggcccgcaccgatcgcccttcccaacagttgcgcagcctgaatggcgaatggcgcctgatgcggtattttctccttacgcatctgtgcggtatttcacaccgcatatggtgcactctcagtacaatctgctctgatgccgcatagttaagccagccccgacacccgccaacacccgctgacgcgccctgacgggcttgtctgctcccggcatccgcttacagacaagctgtgaccgtctccgggagctgcatgtgtcagaggttttcaccgtcatcaccgaaacgcgcgagacgaaagggcctcgtgatacgcctatttttataggttaatgtcatgataataatggtttcttagcaccctttctcggtccttcaacgttcctgacaacgagcctccttttcgccaatccatcgacaatcaccgcgagtccctgctcgaacgctgcgtccggaccggcttcgtcgaaggcgtctatcgcggcccgcaacagcggcgagagcggagcctgttcaacggtgccgccgcgctcgccggcatcgctgtcgccggcctgctcctcaagcacggccccaacagtgaagtagctgattgtcatcagcgcattgacggcgtccccggccgaaaaacccgcctcgcagaggaagcgaagctgcgcgtcggccgtttccatctgcggtgcgcccggtcgcgtgccggcatggatgcgcgcgccatcgcggtaggcgagcagcgcctgcctgaagctgcgggcattcccgatcagaaatgagcgccagtcgtcgtcggctctcggcaccgaatgcgtatgattctccgccagcatggcttcggccagtgcgtcgagcagcgcccgcttgttcctgaagtgccagtaaagcgccggctgctgaacccccaaccgttccgccagtttgcgtgtcgtcagaccgtctacgccgacctcgttcaacaggtccagggcggcacggatcactgtattcggctgcaactttgtcatgattgacactttatcactgataaacataatatgtccaccaacttatcagtgataaagaatccgcgcgttcaatcggaccagcggaggctggtccggaggccagacatgtcatcagcggtggagtgcaatgtcgtgcaatacgaatggcgaaaagccgagctcatcggtcagcttctcaaccttggggttacccccggcggtgtgctgctggtccacagctccttccgtagcgtccggcccctcgaagatgggccacttggactgatcgaggccctgcgtgctgcgctgggtccgggagggacgctcgtcatgccctcgtggtcaggtctggacgacgagccgttcgatcctgccacgtcgcccgttacaccggaccttggagttgtctctgacacattctggcgcctgccaaatgtaaagcgcagcgcccatccatttgcctttgcggcagcggggccacaggcagagcagatcatctctgatccattgcccctgccacctcactcgcctgcaagcccggtcgcccgtgtccatgaactcgatgggcaggtacttctcctcggcgtgggacacgatgccaacacgacgctgcatcttgccgagttgatggcaaaggttccctatggggtgccgagacactgcaccattcttcaggatggcaagttggtacgcgtcgattatctcgagaatgaccactgctgtgagcgctttgccttggcggacaggtggctcaaggagaagagccttcagaaggaaggtccagtcggtcatgcctttgctcggttgatccgctcccgcgacattgtggcgacagccctgggtcaactgggccgagatccgttgatcttcctgcatccgccagaggcgggatgcgaagaatgcgatgccgctcgccagtcgattggctgatcgtggaaacgataggcctatgccatgcgggtcaaggcgacttccggcaagctatacgcgccctagaattgtcaattttaatcctctgtttatcggcagttcgtagagcgcgccgtgcgtcccgagcgatactgagcgaagcaagtgcgtcgagcagtgcccgcttgttcctgaaatgccagtaaagcgctggctgctgaacccccagccggaactgaccccacaaggccctagcgtttgcaatgcaccaggtcatcattgacccaggcgtgttccaccaggccgctgcctcgcaactcttcgcaggcttcgccgacctgctcgcgccacttcttcacgcgggtggaatccgatccgcacatgaggcggaaggtttccagcttgagcgggtacggctcccggtgcgagctgaaatagtcgaacatccgtcgggccgtcggcgacagcttgcggtacttctcccatatgaatttcgtgtagtggtcgccagcaaacagcacgacgatttcctcgtcgatcaggacctggcaacgggacgttttcttgccacggtccaggacgcggaagcggtgcagcagcgacaccgattccaggtgcccaacgcggtcggacgtgaagcccatcgccgtcgcctgtaggcgcgacaggcattcctcggccttcgtgtaataccggccattgatcgaccagcccaggtcctggcaaagctcgtagaacgtgaaggtgatcggctcgccgataggggtgcgcttcgcgtactccaacacctgctgccacaccagttcgtcatcgtcggcccgcagctcgacgccggtgtaggtgatcttcacgtccttgttgacgtggaaaatgaccttgttttgcagcgcctcgcgcgggattttcttgttgcgcgtggtgaacagggcagagcgggccgtgtcgtttggcatcgctcgcatcgtgtccggccacggcgcaatatcgaacaaggaaagctgcatttccttgatctgctgcttcgtgtgtttcagcaacgcggcctgcttggcctcgctgacctgttttgccaggtcctcgccggcggtttttcgcttcttggtcgtcatagttcctcgcgtgtcgatggtcatcgacttcgccaaacctgccgcctcctgttcgagacgacgcgaacgctccacggcggccgatggcgcgggcagggcagggggagccagttgcacgctgtcgcgctcgatcttggccgtagcttgctggaccatcgagccgacggactggaaggtttcgcggggcgcacgcatgacggtgcggcttgcgatggtttcggcatcctcggcggaaaaccccgcgtcgatcagttcttgcctgtatgccttccggtcaaacgtccgattcattcaccctccttgcgggattgccccgactcacgccggggcaatgtgcccttattcctgatttgacccgcctggtgccttggtgtccagataatccaccttatcggcaatgaagtcggtcccgtagaccgtctggccgtccttctcgtacttggtattccgaatcttgccctgcacgaataccagctccgcgaagtcgctcttcttgatggagcgcatggggacgtgcttggcaatcacgcgcaccccccggccgttttagcggctaaaaaagtcatggctctgccctcgggcggaccacgcccatcatgaccttgccaagctcgtcctgcttctcttcgatcttcgccagcagggcgaggatcgtggcatcaccgaaccgcgccgtgcgcgggtcgtcggtgagccagagtttcagcaggccgcccaggcggcccaggtcgccattgatgcgggccagctcgcggacgtgctcatagtccacgacgcccgtgattttgtagccctggccgacggccagcaggtaggcctacaggctcatgccggccgccgccgccttttcctcaatcgctcttcgttcgtctggaaggcagtacaccttgataggtgggctgcccttcctggttggcttggtttcatcagccatccgcttgccctcatctgttacgccggcggtagccggccagcctcgcagagcaggattcccgttgagcaccgccaggtgcgaataagggacagtgaagaaggaacacccgctcgcgggtgggcctacttcacctatcctgcccggctgacgccgttggatacaccaaggaaagtctacacgaaccctttggcaaaatcctgtatatcgtgcgaaaaaggatggatataccgaaaaaatcgctataatgaccccgaagcagggttatgcagcggaaaagatccgtc-3´

**pCM130-*P_mxaF_** , the sequences in red represent promoter *P_mxaF_****

5´-

gaccctttccgacgctcaccgggctggttgccctcgccgctgggctggcggccgtctatggccctgcaaacgcgccagaaacgccgtcgaagccgtgtgcgagacaccgcggccgccggcgttgtggatacctcgcggaaaacttggccctcactgacagatgaggggcggacgttgacacttgaggggccgactcacccggcgcggcgttgacagatgaggggcaggctcgatttcggccggcgacgtggagctggccagcctcgcaaatcggcgaaaacgcctgattttacgcgagtttcccacagatgatgtggacaagcctggggataagtgccctgcggtattgacacttgaggggcgcgactactgacagatgaggggcgcgatccttgacacttgaggggcagagtgctgacagatgaggggcgcacctattgacatttgaggggctgtccacaggcagaaaatccagcatttgcaagggtttccgcccgtttttcggccaccgctaacctgtcttttaacctgcttttaaaccaatatttataaaccttgtttttaaccagggctgcgccctgtgcgcgtgaccgcgcacgccgaaggggggtgcccccccttctcgaaccctcccggcccgctaacgcgggcctcccatccccccaggggctgcgcccctcggccgcgaacggcctcaccccaaaaatggcagccaagctgaccacttctgcgctcggcccttccggctggctggtttattgctgataaatctggagccggtgagcgtgggtctcgcggtatcattgcagcactggggccagatggtaagccctcccgtatcgtagttatctacacgacggggagtcaggcaactatggatgaacgaaatagacagatcgctgagataggtgcctcactgattaagcattggtaactgtcagaccaagtttactcatatatactttagattgatttaaaacttcatttttaatttaaaaggatctaggtgaagatcctttttgataatctcatgaccaaaatcccttaacgtgagttttcgttccactgagcgtcagaccccgtagaaaagatcaaaggatcttcttgagatcctttttttctgcgcgtaatctgctgcttgcaaacaaaaaaaccaccgctaccagcggtggtttgtttgccggatcaagagctaccaactctttttccgaaggtaactggcttcagcagagcgcagataccaaatactgtccttctagtgtagccgtagttaggccaccacttcaagaactctgtagcaccgcctacatacctcgctctgctaatcctgttaccagtggctgctgccagtggcgataagtcgtgtcttaccgggttggactcaagacgatagttaccggataaggcgcagcggtcgggctgaacggggggttcgtgcacacagcccagcttggagcgaacgacctacaccgaactgagatacctacagcgtgagctatgagaaagcgccacgcttcccgaagggagaaaggcggacaggtatccggtaagcggcagggtcggaacaggagagcgcacgagggagcttccagggggaaacgcctggtatctttatagtcctgtcgggtttcgccacctctgacttgagcgtcgatttttgtgatgctcgtcaggggggcggagcctatggaaaaacgccagcaacgcggcctttttacggttcctggccttttgctggccttttgctcacatggtacccgggagctccatcaggtgagcacggtcatgaatcgttcgttgagaatgcggtcgtggtaaaagatcgccttgcccagctggtcggtggtccaggtcaccggtttgtggtccgggtagttgtaatctcccccgcagaacacttcgttgcggttaccggacgggtcgaagaagtagatggtcttgccgtgagtgaggccgtggcgggttgggccgatatcgatagatgtgtcggtcatggagatcaggtcggcggcgcgaagcaagtcttcccaggtttcgaggtggaaggacacatgatggaggcggcctttttccggatggtgaatgaaggccacgtcgtgggccttggtcgacagactgagaaactgggcgacgcgcgtgccattttcgtccagcacctgttcggccagatagaaaccgagcaccttggtgaacaggtcataggtcgccggcaattcgtcgccatacatgagggcgtggtcgaaacgcacagccgccatacctttcagatcgcgcggccatgcctcgggattgacgtcattcaaaccccactttccagtatattccttgtctgcatacaactcgaagtgatgcccggagggggcctggaagcgcacgcgccggccacaactgttcagttcacctgcgggtagctgctcaacggcacagccatatgccatcagatcccgctccagttgccggagagcatcctcatccacaaccttgaaacccataaaatccatgcccggctcgtcagcctcgcgtagcaccagggaaaacttatccacttcggtccaagccttcagatagacacggccctggtcgtcacggtccatctcgatcaggcccagcaactcgacgtagtgttccagggccttgctcatgtccagtacacgcagctgcacatggcccggtcgcattacacctttgttcatgacgtcacctcctgaagcttGCGGTATCTCTCAGACGTTTTTCTGAGGTTCCAGGCTCGCTCGGAAGTCATCCGAAGTGCCGCATCGTGAATCTAAGGATATTCTTAGATTTGCGCGGTCTTATGAAAGACTTATTAGCGTCCCTTCGCCTATATTTTCTAGGCTTTGATTGGACGCGATGTCTTTAGCTGGTCGGACATTTCTTATCAGGACGATAAGGCAATTCTAAAAGTGCCAAGGCGTAGTGTCGCAACGTATTGGCATCGTCTTGACCGGGGCCGGGACCCATCGCACCGTTGTCGTCAACGGGCCCTCGCGAAGCGGCCCGACCAAGCGGGggatccccgggtaccgagctcgaatctagaattccctgctttcctgatgcaaaaacgaggctagtttaccgtatctgtggggggatggcttgtagatatgacgacaggaagagtttgtagaaacgcaaaaaggccatccgtcaggatggccttctgcttaatttgatgcctggcagtttatggcgggcgtcctgcccgccaccctccgggccgttgcttcgcaacgttcaaatccgctcccggcggatttgtcctactcaggagagcgttcaccgacaaacaacagataaaacgaaaggcccagtctttcgactgagcctttcgttttatttgatgcctggcagttccctactctcgcatggggagaccccacactaccatcggcgctacggcgtttcacttctgagttcggcatggggtcaggtgggaccaccgcgctactgccgccaggcaaattctgttttatcagaccgcttctgcgttctgatttaatctgtatcaggctgaaaaattcactggccgtcgttttacaacgtcgtgactgggaaaaccctggcgttacccaacttaatcgccttgcagcacatccccctttcgccagctggcgtaatagcgaagaggcccgcaccgatcgcccttcccaacagttgcgcagcctgaatggcgaatggcgcctgatgcggtattttctccttacgcatctgtgcggtatttcacaccgcatatggtgcactctcagtacaatctgctctgatgccgcatagttaagccagccccgacacccgccaacacccgctgacgcgccctgacgggcttgtctgctcccggcatccgcttacagacaagctgtgaccgtctccgggagctgcatgtgtcagaggttttcaccgtcatcaccgaaacgcgcgagacgaaagggcctcgtgatacgcctatttttataggttaatgtcatgataataatggtttcttagcaccctttctcggtccttcaacgttcctgacaacgagcctccttttcgccaatccatcgacaatcaccgcgagtccctgctcgaacgctgcgtccggaccggcttcgtcgaaggcgtctatcgcggcccgcaacagcggcgagagcggagcctgttcaacggtgccgccgcgctcgccggcatcgctgtcgccggcctgctcctcaagcacggccccaacagtgaagtagctgattgtcatcagcgcattgacggcgtccccggccgaaaaacccgcctcgcagaggaagcgaagctgcgcgtcggccgtttccatctgcggtgcgcccggtcgcgtgccggcatggatgcgcgcgccatcgcggtaggcgagcagcgcctgcctgaagctgcgggcattcccgatcagaaatgagcgccagtcgtcgtcggctctcggcaccgaatgcgtatgattctccgccagcatggcttcggccagtgcgtcgagcagcgcccgcttgttcctgaagtgccagtaaagcgccggctgctgaacccccaaccgttccgccagtttgcgtgtcgtcagaccgtctacgccgacctcgttcaacaggtccagggcggcacggatcactgtattcggctgcaactttgtcatgattgacactttatcactgataaacataatatgtccaccaacttatcagtgataaagaatccgcgcgttcaatcggaccagcggaggctggtccggaggccagacgtgaaacccaacatacccctgatcgtaattctgagcactgtcgcgctcgacgctgtcggcatcggcctgattatgccggtgctgccgggcctcctgcgcgatctggttcactcgaacgacgtcaccgcccactatggcattctgctggcgctgtatgcgttggtgcaatttgcctgcgcacctgtgctgggcgcgctgtcggatcgtttcgggcggcggccaatcttgctcgtctcgctggccggcgccactgtcgactacgccatcatggcgacagcgcctttcctttgggttctctatatcgggcggatcgtggccggcatcaccggggcgactggggcggtagccggcgcttatattgccgatatcactgatggcgatgagcgcgcgcggcacttcggcttcatgagcgcctgtttcgggttcgggatggtcgcgggacctgtgctcggtgggctgatgggcggtttctccccccacgctccgttcttcgccgcggcagccttgaacggcctcaatttcctgacgggctgtttccttttgccggagtcgcacaaaggcgaacgccggccgttacgccgggaggctctcaacccgctcgcttcgttccggtgggcccggggcatgaccgtcgtcgccgccctgatggcggtcttcttcatcatgcaacttgtcggacaggtgccggccgcgctttgggtcattttcggcgaggatcgctttcactgggacgcgaccacgatcggcatttcgcttgccgcatttggcattctgcattcactcgcccaggcaatgatcaccggccctgtagccgcccggctcggcgaaaggcgggcactcatgctcggaatgattgccgacggcacaggctacatcctgcttgccttcgcgacacggggatggatggcgttcccgatcatggtcctgcttgcttcgggtggcatcggaatgccggcgctgcaagcaatgttgtccaggcaggtggatgaggaacgtcaggggcagctgcaaggctcactggcggcgctcaccagcctgacctcgatcgtcggacccctcctcttcacggcgatctatgcggcttctataacaacgtggaacgggtgggcatggattgcaggcgctgccctctacttgctctgcctgccggcgctgcgtcgcgggctttggagcggcgcagggcaacgagccgatcgctgatcgtggaaacgataggcctatgccatgcgggtcaaggcgacttccggcaagctatacgcgccctagaattgtcaattttaatcctctgtttatcggcagttcgtagagcgcgccgtgcgtcccgagcgatactgagcgaagcaagtgcgtcgagcagtgcccgcttgttcctgaaatgccagtaaagcgctggctgctgaacccccagccggaactgaccccacaaggccctagcgtttgcaatgcaccaggtcatcattgacccaggcgtgttccaccaggccgctgcctcgcaactcttcgcaggcttcgccgacctgctcgcgccacttcttcacgcgggtggaatccgatccgcacatgaggcggaaggtttccagcttgagcgggtacggctcccggtgcgagctgaaatagtcgaacatccgtcgggccgtcggcgacagcttgcggtacttctcccatatgaatttcgtgtagtggtcgccagcaaacagcacgacgatttcctcgtcgatcaggacctggcaacgggacgttttcttgccacggtccaggacgcggaagcggtgcagcagcgacaccgattccaggtgcccaacgcggtcggacgtgaagcccatcgccgtcgcctgtaggcgcgacaggcattcctcggccttcgtgtaataccggccattgatcgaccagcccaggtcctggcaaagctcgtagaacgtgaaggtgatcggctcgccgataggggtgcgcttcgcgtactccaacacctgctgccacaccagttcgtcatcgtcggcccgcagctcgacgccggtgtaggtgatcttcacgtccttgttgacgtggaaaatgaccttgttttgcagcgcctcgcgcgggattttcttgttgcgcgtggtgaacagggcagagcgggccgtgtcgtttggcatcgctcgcatcgtgtccggccacggcgcaatatcgaacaaggaaagctgcatttccttgatctgctgcttcgtgtgtttcagcaacgcggcctgcttggcctcgctgacctgttttgccaggtcctcgccggcggtttttcgcttcttggtcgtcatagttcctcgcgtgtcgatggtcatcgacttcgccaaacctgccgcctcctgttcgagacgacgcgaacgctccacggcggccgatggcgcgggcagggcagggggagccagttgcacgctgtcgcgctcgatcttggccgtagcttgctggaccatcgagccgacggactggaaggtttcgcggggcgcacgcatgacggtgcggcttgcgatggtttcggcatcctcggcggaaaaccccgcgtcgatcagttcttgcctgtatgccttccggtcaaacgtccgattcattcaccctccttgcgggattgccccgactcacgccggggcaatgtgcccttattcctgatttgacccgcctggtgccttggtgtccagataatccaccttatcggcaatgaagtcggtcccgtagaccgtctggccgtccttctcgtacttggtattccgaatcttgccctgcacgaataccagctccgcgaagtcgctcttcttgatggagcgcatggggacgtgcttggcaatcacgcgcaccccccggccgttttagcggctaaaaaagtcatggctctgccctcgggcggaccacgcccatcatgaccttgccaagctcgtcctgcttctcttcgatcttcgccagcagggcgaggatcgtggcatcaccgaaccgcgccgtgcgcgggtcgtcggtgagccagagtttcagcaggccgcccaggcggcccaggtcgccattgatgcgggccagctcgcggacgtgctcatagtccacgacgcccgtgattttgtagccctggccgacggccagcaggtaggcctacaggctcatgccggccgccgccgccttttcctcaatcgctcttcgttcgtctggaaggcagtacaccttgataggtgggctgcccttcctggttggcttggtttcatcagccatccgcttgccctcatctgttacgccggcggtagccggccagcctcgcagagcaggattcccgttgagcaccgccaggtgcgaataagggacagtgaagaaggaacacccgctcgcgggtgggcctacttcacctatcctgcccggctgacgccgttggatacaccaaggaaagtctacacgaaccctttggcaaaatcctgtatatcgtgcgaaaaaggatggatataccgaaaaaatcgctataatgaccccgaagcagggttatgcagcggaaaagatccgtc-3´

**pCM130-*P_mxaF_*, the sequences in red represent promoter *P_mxaF_***

5´-

gaccctttccgacgctcaccgggctggttgccctcgccgctgggctggcggccgtctatggccctgcaaacgcgccagaaacgccgtcgaagccgtgtgcgagacaccgcggccgccggcgttgtggatacctcgcggaaaacttggccctcactgacagatgaggggcggacgttgacacttgaggggccgactcacccggcgcggcgttgacagatgaggggcaggctcgatttcggccggcgacgtggagctggccagcctcgcaaatcggcgaaaacgcctgattttacgcgagtttcccacagatgatgtggacaagcctggggataagtgccctgcggtattgacacttgaggggcgcgactactgacagatgaggggcgcgatccttgacacttgaggggcagagtgctgacagatgaggggcgcacctattgacatttgaggggctgtccacaggcagaaaatccagcatttgcaagggtttccgcccgtttttcggccaccgctaacctgtcttttaacctgcttttaaaccaatatttataaaccttgtttttaaccagggctgcgccctgtgcgcgtgaccgcgcacgccgaaggggggtgcccccccttctcgaaccctcccggcccgctaacgcgggcctcccatccccccaggggctgcgcccctcggccgcgaacggcctcaccccaaaaatggcagccaagctgaccacttctgcgctcggcccttccggctggctggtttattgctgataaatctggagccggtgagcgtgggtctcgcggtatcattgcagcactggggccagatggtaagccctcccgtatcgtagttatctacacgacggggagtcaggcaactatggatgaacgaaatagacagatcgctgagataggtgcctcactgattaagcattggtaactgtcagaccaagtttactcatatatactttagattgatttaaaacttcatttttaatttaaaaggatctaggtgaagatcctttttgataatctcatgaccaaaatcccttaacgtgagttttcgttccactgagcgtcagaccccgtagaaaagatcaaaggatcttcttgagatcctttttttctgcgcgtaatctgctgcttgcaaacaaaaaaaccaccgctaccagcggtggtttgtttgccggatcaagagctaccaactctttttccgaaggtaactggcttcagcagagcgcagataccaaatactgtccttctagtgtagccgtagttaggccaccacttcaagaactctgtagcaccgcctacatacctcgctctgctaatcctgttaccagtggctgctgccagtggcgataagtcgtgtcttaccgggttggactcaagacgatagttaccggataaggcgcagcggtcgggctgaacggggggttcgtgcacacagcccagcttggagcgaacgacctacaccgaactgagatacctacagcgtgagctatgagaaagcgccacgcttcccgaagggagaaaggcggacaggtatccggtaagcggcagggtcggaacaggagagcgcacgagggagcttccagggggaaacgcctggtatctttatagtcctgtcgggtttcgccacctctgacttgagcgtcgatttttgtgatgctcgtcaggggggcggagcctatggaaaaacgccagcaacgcggcctttttacggttcctggccttttgctggccttttgctcacatggtacccgggagctccatcaggtgagcacggtcatgaatcgttcgttgagaatgcggtcgtggtaaaagatcgccttgcccagctggtcggtggtccaggtcaccggtttgtggtccgggtagttgtaatctcccccgcagaacacttcgttgcggttaccggacgggtcgaagaagtagatggtcttgccgtgagtgaggccgtggcgggttgggccgatatcgatagatgtgtcggtcatggagatcaggtcggcggcgcgaagcaagtcttcccaggtttcgaggtggaaggacacatgatggaggcggcctttttccggatggtgaatgaaggccacgtcgtgggccttggtcgacagactgagaaactgggcgacgcgcgtgccattttcgtccagcacctgttcggccagatagaaaccgagcaccttggtgaacaggtcataggtcgccggcaattcgtcgccatacatgagggcgtggtcgaaacgcacagccgccatacctttcagatcgcgcggccatgcctcgggattgacgtcattcaaaccccactttccagtatattccttgtctgcatacaactcgaagtgatgcccggagggggcctggaagcgcacgcgccggccacaactgttcagttcacctgcgggtagctgctcaacggcacagccatatgccatcagatcccgctccagttgccggagagcatcctcatccacaaccttgaaacccataaaatccatgcccggctcgtcagcctcgcgtagcaccagggaaaacttatccacttcggtccaagccttcagatagacacggccctggtcgtcacggtccatctcgatcaggcccagcaactcgacgtagtgttccagggccttgctcatgtccagtacacgcagctgcacatggcccggtcgcattacacctttgttcatgacgtcacctcctgaagcttGCGGTATCTCTCAGACGTTTTTCTGAGGTTCCAGGCTCGCTCGGAAGTCATCCGAAGTGCCGCATCGTGAATCTAAGGATATTTTTAGATTTGCGCGGTCTTATGAAAGACTTATTAGCGTCCCTTCGCCTATATTTTCTAGGCTTTGATTGGACGCGATGTCTTTAGCTGGTCGGACATTTCTTATCAGGACGATAAGGCAATTCTAAAAGTGCCAAGGCGTAGTGTCGCAACGTATTGGCATCGTCTTGACCGGGGCCGGGACCCATCGCACCGTTGTCGTCAACGGGCCCTCGCGAAGCGGCCCGACCAAGCGGGggatccccgggtaccgagctcgaatctagaattccctgctttcctgatgcaaaaacgaggctagtttaccgtatctgtggggggatggcttgtagatatgacgacaggaagagtttgtagaaacgcaaaaaggccatccgtcaggatggccttctgcttaatttgatgcctggcagtttatggcgggcgtcctgcccgccaccctccgggccgttgcttcgcaacgttcaaatccgctcccggcggatttgtcctactcaggagagcgttcaccgacaaacaacagataaaacgaaaggcccagtctttcgactgagcctttcgttttatttgatgcctggcagttccctactctcgcatggggagaccccacactaccatcggcgctacggcgtttcacttctgagttcggcatggggtcaggtgggaccaccgcgctactgccgccaggcaaattctgttttatcagaccgcttctgcgttctgatttaatctgtatcaggctgaaaaattcactggccgtcgttttacaacgtcgtgactgggaaaaccctggcgttacccaacttaatcgccttgcagcacatccccctttcgccagctggcgtaatagcgaagaggcccgcaccgatcgcccttcccaacagttgcgcagcctgaatggcgaatggcgcctgatgcggtattttctccttacgcatctgtgcggtatttcacaccgcatatggtgcactctcagtacaatctgctctgatgccgcatagttaagccagccccgacacccgccaacacccgctgacgcgccctgacgggcttgtctgctcccggcatccgcttacagacaagctgtgaccgtctccgggagctgcatgtgtcagaggttttcaccgtcatcaccgaaacgcgcgagacgaaagggcctcgtgatacgcctatttttataggttaatgtcatgataataatggtttcttagcaccctttctcggtccttcaacgttcctgacaacgagcctccttttcgccaatccatcgacaatcaccgcgagtccctgctcgaacgctgcgtccggaccggcttcgtcgaaggcgtctatcgcggcccgcaacagcggcgagagcggagcctgttcaacggtgccgccgcgctcgccggcatcgctgtcgccggcctgctcctcaagcacggccccaacagtgaagtagctgattgtcatcagcgcattgacggcgtccccggccgaaaaacccgcctcgcagaggaagcgaagctgcgcgtcggccgtttccatctgcggtgcgcccggtcgcgtgccggcatggatgcgcgcgccatcgcggtaggcgagcagcgcctgcctgaagctgcgggcattcccgatcagaaatgagcgccagtcgtcgtcggctctcggcaccgaatgcgtatgattctccgccagcatggcttcggccagtgcgtcgagcagcgcccgcttgttcctgaagtgccagtaaagcgccggctgctgaacccccaaccgttccgccagtttgcgtgtcgtcagaccgtctacgccgacctcgttcaacaggtccagggcggcacggatcactgtattcggctgcaactttgtcatgattgacactttatcactgataaacataatatgtccaccaacttatcagtgataaagaatccgcgcgttcaatcggaccagcggaggctggtccggaggccagacgtgaaacccaacatacccctgatcgtaattctgagcactgtcgcgctcgacgctgtcggcatcggcctgattatgccggtgctgccgggcctcctgcgcgatctggttcactcgaacgacgtcaccgcccactatggcattctgctggcgctgtatgcgttggtgcaatttgcctgcgcacctgtgctgggcgcgctgtcggatcgtttcgggcggcggccaatcttgctcgtctcgctggccggcgccactgtcgactacgccatcatggcgacagcgcctttcctttgggttctctatatcgggcggatcgtggccggcatcaccggggcgactggggcggtagccggcgcttatattgccgatatcactgatggcgatgagcgcgcgcggcacttcggcttcatgagcgcctgtttcgggttcgggatggtcgcgggacctgtgctcggtgggctgatgggcggtttctccccccacgctccgttcttcgccgcggcagccttgaacggcctcaatttcctgacgggctgtttccttttgccggagtcgcacaaaggcgaacgccggccgttacgccgggaggctctcaacccgctcgcttcgttccggtgggcccggggcatgaccgtcgtcgccgccctgatggcggtcttcttcatcatgcaacttgtcggacaggtgccggccgcgctttgggtcattttcggcgaggatcgctttcactgggacgcgaccacgatcggcatttcgcttgccgcatttggcattctgcattcactcgcccaggcaatgatcaccggccctgtagccgcccggctcggcgaaaggcgggcactcatgctcggaatgattgccgacggcacaggctacatcctgcttgccttcgcgacacggggatggatggcgttcccgatcatggtcctgcttgcttcgggtggcatcggaatgccggcgctgcaagcaatgttgtccaggcaggtggatgaggaacgtcaggggcagctgcaaggctcactggcggcgctcaccagcctgacctcgatcgtcggacccctcctcttcacggcgatctatgcggcttctataacaacgtggaacgggtgggcatggattgcaggcgctgccctctacttgctctgcctgccggcgctgcgtcgcgggctttggagcggcgcagggcaacgagccgatcgctgatcgtggaaacgataggcctatgccatgcgggtcaaggcgacttccggcaagctatacgcgccctagaattgtcaattttaatcctctgtttatcggcagttcgtagagcgcgccgtgcgtcccgagcgatactgagcgaagcaagtgcgtcgagcagtgcccgcttgttcctgaaatgccagtaaagcgctggctgctgaacccccagccggaactgaccccacaaggccctagcgtttgcaatgcaccaggtcatcattgacccaggcgtgttccaccaggccgctgcctcgcaactcttcgcaggcttcgccgacctgctcgcgccacttcttcacgcgggtggaatccgatccgcacatgaggcggaaggtttccagcttgagcgggtacggctcccggtgcgagctgaaatagtcgaacatccgtcgggccgtcggcgacagcttgcggtacttctcccatatgaatttcgtgtagtggtcgccagcaaacagcacgacgatttcctcgtcgatcaggacctggcaacgggacgttttcttgccacggtccaggacgcggaagcggtgcagcagcgacaccgattccaggtgcccaacgcggtcggacgtgaagcccatcgccgtcgcctgtaggcgcgacaggcattcctcggccttcgtgtaataccggccattgatcgaccagcccaggtcctggcaaagctcgtagaacgtgaaggtgatcggctcgccgataggggtgcgcttcgcgtactccaacacctgctgccacaccagttcgtcatcgtcggcccgcagctcgacgccggtgtaggtgatcttcacgtccttgttgacgtggaaaatgaccttgttttgcagcgcctcgcgcgggattttcttgttgcgcgtggtgaacagggcagagcgggccgtgtcgtttggcatcgctcgcatcgtgtccggccacggcgcaatatcgaacaaggaaagctgcatttccttgatctgctgcttcgtgtgtttcagcaacgcggcctgcttggcctcgctgacctgttttgccaggtcctcgccggcggtttttcgcttcttggtcgtcatagttcctcgcgtgtcgatggtcatcgacttcgccaaacctgccgcctcctgttcgagacgacgcgaacgctccacggcggccgatggcgcgggcagggcagggggagccagttgcacgctgtcgcgctcgatcttggccgtagcttgctggaccatcgagccgacggactggaaggtttcgcggggcgcacgcatgacggtgcggcttgcgatggtttcggcatcctcggcggaaaaccccgcgtcgatcagttcttgcctgtatgccttccggtcaaacgtccgattcattcaccctccttgcgggattgccccgactcacgccggggcaatgtgcccttattcctgatttgacccgcctggtgccttggtgtccagataatccaccttatcggcaatgaagtcggtcccgtagaccgtctggccgtccttctcgtacttggtattccgaatcttgccctgcacgaataccagctccgcgaagtcgctcttcttgatggagcgcatggggacgtgcttggcaatcacgcgcaccccccggccgttttagcggctaaaaaagtcatggctctgccctcgggcggaccacgcccatcatgaccttgccaagctcgtcctgcttctcttcgatcttcgccagcagggcgaggatcgtggcatcaccgaaccgcgccgtgcgcgggtcgtcggtgagccagagtttcagcaggccgcccaggcggcccaggtcgccattgatgcgggccagctcgcggacgtgctcatagtccacgacgcccgtgattttgtagccctggccgacggccagcaggtaggcctacaggctcatgccggccgccgccgccttttcctcaatcgctcttcgttcgtctggaaggcagtacaccttgataggtgggctgcccttcctggttggcttggtttcatcagccatccgcttgccctcatctgttacgccggcggtagccggccagcctcgcagagcaggattcccgttgagcaccgccaggtgcgaataagggacagtgaagaaggaacacccgctcgcgggtgggcctacttcacctatcctgcccggctgacgccgttggatacaccaaggaaagtctacacgaaccctttggcaaaatcctgtatatcgtgcgaaaaaggatggatataccgaaaaaatcgctataatgaccccgaagcagggttatgcagcggaaaagatccgtc-3´

**pCM130-*P_fdh2*_*, the sequences in red represent the promoter *P_fdh2*_***

5´-

gaccctttccgacgctcaccgggctggttgccctcgccgctgggctggcggccgtctatggccctgcaaacgcgccagaaacgccgtcgaagccgtgtgcgagacaccgcggccgccggcgttgtggatacctcgcggaaaacttggccctcactgacagatgaggggcggacgttgacacttgaggggccgactcacccggcgcggcgttgacagatgaggggcaggctcgatttcggccggcgacgtggagctggccagcctcgcaaatcggcgaaaacgcctgattttacgcgagtttcccacagatgatgtggacaagcctggggataagtgccctgcggtattgacacttgaggggcgcgactactgacagatgaggggcgcgatccttgacacttgaggggcagagtgctgacagatgaggggcgcacctattgacatttgaggggctgtccacaggcagaaaatccagcatttgcaagggtttccgcccgtttttcggccaccgctaacctgtcttttaacctgcttttaaaccaatatttataaaccttgtttttaaccagggctgcgccctgtgcgcgtgaccgcgcacgccgaaggggggtgcccccccttctcgaaccctcccggcccgctaacgcgggcctcccatccccccaggggctgcgcccctcggccgcgaacggcctcaccccaaaaatggcagccaagctgaccacttctgcgctcggcccttccggctggctggtttattgctgataaatctggagccggtgagcgtgggtctcgcggtatcattgcagcactggggccagatggtaagccctcccgtatcgtagttatctacacgacggggagtcaggcaactatggatgaacgaaatagacagatcgctgagataggtgcctcactgattaagcattggtaactgtcagaccaagtttactcatatatactttagattgatttaaaacttcatttttaatttaaaaggatctaggtgaagatcctttttgataatctcatgaccaaaatcccttaacgtgagttttcgttccactgagcgtcagaccccgtagaaaagatcaaaggatcttcttgagatcctttttttctgcgcgtaatctgctgcttgcaaacaaaaaaaccaccgctaccagcggtggtttgtttgccggatcaagagctaccaactctttttccgaaggtaactggcttcagcagagcgcagataccaaatactgtccttctagtgtagccgtagttaggccaccacttcaagaactctgtagcaccgcctacatacctcgctctgctaatcctgttaccagtggctgctgccagtggcgataagtcgtgtcttaccgggttggactcaagacgatagttaccggataaggcgcagcggtcgggctgaacggggggttcgtgcacacagcccagcttggagcgaacgacctacaccgaactgagatacctacagcgtgagctatgagaaagcgccacgcttcccgaagggagaaaggcggacaggtatccggtaagcggcagggtcggaacaggagagcgcacgagggagcttccagggggaaacgcctggtatctttatagtcctgtcgggtttcgccacctctgacttgagcgtcgatttttgtgatgctcgtcaggggggcggagcctatggaaaaacgccagcaacgcggcctttttacggttcctggccttttgctggccttttgctcacatggtacccgggagctccatcaggtgagcacggtcatgaatcgttcgttgagaatgcggtcgtggtaaaagatcgccttgcccagctggtcggtggtccaggtcaccggtttgtggtccgggtagttgtaatctcccccgcagaacacttcgttgcggttaccggacgggtcgaagaagtagatggtcttgccgtgagtgaggccgtggcgggttgggccgatatcgatagatgtgtcggtcatggagatcaggtcggcggcgcgaagcaagtcttcccaggtttcgaggtggaaggacacatgatggaggcggcctttttccggatggtgaatgaaggccacgtcgtgggccttggtcgacagactgagaaactgggcgacgcgcgtgccattttcgtccagcacctgttcggccagatagaaaccgagcaccttggtgaacaggtcataggtcgccggcaattcgtcgccatacatgagggcgtggtcgaaacgcacagccgccatacctttcagatcgcgcggccatgcctcgggattgacgtcattcaaaccccactttccagtatattccttgtctgcatacaactcgaagtgatgcccggagggggcctggaagcgcacgcgccggccacaactgttcagttcacctgcgggtagctgctcaacggcacagccatatgccatcagatcccgctccagttgccggagagcatcctcatccacaaccttgaaacccataaaatccatgcccggctcgtcagcctcgcgtagcaccagggaaaacttatccacttcggtccaagccttcagatagacacggccctggtcgtcacggtccatctcgatcaggcccagcaactcgacgtagtgttccagggccttgctcatgtccagtacacgcagctgcacatggcccggtcgcattacacctttgttcatgacgtcacctcctgaagcttCGTTTCCTGCCCAACTTGGTTTTGTTCTAAGGCCGGATCCAAACAGGGCCGGCACTAAAGTTCAATCAAGCAGTCTCGTGGTTTGACAAAAATCTTCGATCAAAACGAGTCAGTTGCAGTGCATCATCCGCTCAAAGGCGGTTCAAACTTTTTTTGAAGCGGCGAAAAATTTTCTTCGAGGCCATCGGGggatccccgggtaccgagctcgaatctagaattccctgctttcctgatgcaaaaacgaggctagtttaccgtatctgtggggggatggcttgtagatatgacgacaggaagagtttgtagaaacgcaaaaaggccatccgtcaggatggccttctgcttaatttgatgcctggcagtttatggcgggcgtcctgcccgccaccctccgggccgttgcttcgcaacgttcaaatccgctcccggcggatttgtcctactcaggagagcgttcaccgacaaacaacagataaaacgaaaggcccagtctttcgactgagcctttcgttttatttgatgcctggcagttccctactctcgcatggggagaccccacactaccatcggcgctacggcgtttcacttctgagttcggcatggggtcaggtgggaccaccgcgctactgccgccaggcaaattctgttttatcagaccgcttctgcgttctgatttaatctgtatcaggctgaaaaattcactggccgtcgttttacaacgtcgtgactgggaaaaccctggcgttacccaacttaatcgccttgcagcacatccccctttcgccagctggcgtaatagcgaagaggcccgcaccgatcgcccttcccaacagttgcgcagcctgaatggcgaatggcgcctgatgcggtattttctccttacgcatctgtgcggtatttcacaccgcatatggtgcactctcagtacaatctgctctgatgccgcatagttaagccagccccgacacccgccaacacccgctgacgcgccctgacgggcttgtctgctcccggcatccgcttacagacaagctgtgaccgtctccgggagctgcatgtgtcagaggttttcaccgtcatcaccgaaacgcgcgagacgaaagggcctcgtgatacgcctatttttataggttaatgtcatgataataatggtttcttagcaccctttctcggtccttcaacgttcctgacaacgagcctccttttcgccaatccatcgacaatcaccgcgagtccctgctcgaacgctgcgtccggaccggcttcgtcgaaggcgtctatcgcggcccgcaacagcggcgagagcggagcctgttcaacggtgccgccgcgctcgccggcatcgctgtcgccggcctgctcctcaagcacggccccaacagtgaagtagctgattgtcatcagcgcattgacggcgtccccggccgaaaaacccgcctcgcagaggaagcgaagctgcgcgtcggccgtttccatctgcggtgcgcccggtcgcgtgccggcatggatgcgcgcgccatcgcggtaggcgagcagcgcctgcctgaagctgcgggcattcccgatcagaaatgagcgccagtcgtcgtcggctctcggcaccgaatgcgtatgattctccgccagcatggcttcggccagtgcgtcgagcagcgcccgcttgttcctgaagtgccagtaaagcgccggctgctgaacccccaaccgttccgccagtttgcgtgtcgtcagaccgtctacgccgacctcgttcaacaggtccagggcggcacggatcactgtattcggctgcaactttgtcatgattgacactttatcactgataaacataatatgtccaccaacttatcagtgataaagaatccgcgcgttcaatcggaccagcggaggctggtccggaggccagacgtgaaacccaacatacccctgatcgtaattctgagcactgtcgcgctcgacgctgtcggcatcggcctgattatgccggtgctgccgggcctcctgcgcgatctggttcactcgaacgacgtcaccgcccactatggcattctgctggcgctgtatgcgttggtgcaatttgcctgcgcacctgtgctgggcgcgctgtcggatcgtttcgggcggcggccaatcttgctcgtctcgctggccggcgccactgtcgactacgccatcatggcgacagcgcctttcctttgggttctctatatcgggcggatcgtggccggcatcaccggggcgactggggcggtagccggcgcttatattgccgatatcactgatggcgatgagcgcgcgcggcacttcggcttcatgagcgcctgtttcgggttcgggatggtcgcgggacctgtgctcggtgggctgatgggcggtttctccccccacgctccgttcttcgccgcggcagccttgaacggcctcaatttcctgacgggctgtttccttttgccggagtcgcacaaaggcgaacgccggccgttacgccgggaggctctcaacccgctcgcttcgttccggtgggcccggggcatgaccgtcgtcgccgccctgatggcggtcttcttcatcatgcaacttgtcggacaggtgccggccgcgctttgggtcattttcggcgaggatcgctttcactgggacgcgaccacgatcggcatttcgcttgccgcatttggcattctgcattcactcgcccaggcaatgatcaccggccctgtagccgcccggctcggcgaaaggcgggcactcatgctcggaatgattgccgacggcacaggctacatcctgcttgccttcgcgacacggggatggatggcgttcccgatcatggtcctgcttgcttcgggtggcatcggaatgccggcgctgcaagcaatgttgtccaggcaggtggatgaggaacgtcaggggcagctgcaaggctcactggcggcgctcaccagcctgacctcgatcgtcggacccctcctcttcacggcgatctatgcggcttctataacaacgtggaacgggtgggcatggattgcaggcgctgccctctacttgctctgcctgccggcgctgcgtcgcgggctttggagcggcgcagggcaacgagccgatcgctgatcgtggaaacgataggcctatgccatgcgggtcaaggcgacttccggcaagctatacgcgccctagaattgtcaattttaatcctctgtttatcggcagttcgtagagcgcgccgtgcgtcccgagcgatactgagcgaagcaagtgcgtcgagcagtgcccgcttgttcctgaaatgccagtaaagcgctggctgctgaacccccagccggaactgaccccacaaggccctagcgtttgcaatgcaccaggtcatcattgacccaggcgtgttccaccaggccgctgcctcgcaactcttcgcaggcttcgccgacctgctcgcgccacttcttcacgcgggtggaatccgatccgcacatgaggcggaaggtttccagcttgagcgggtacggctcccggtgcgagctgaaatagtcgaacatccgtcgggccgtcggcgacagcttgcggtacttctcccatatgaatttcgtgtagtggtcgccagcaaacagcacgacgatttcctcgtcgatcaggacctggcaacgggacgttttcttgccacggtccaggacgcggaagcggtgcagcagcgacaccgattccaggtgcccaacgcggtcggacgtgaagcccatcgccgtcgcctgtaggcgcgacaggcattcctcggccttcgtgtaataccggccattgatcgaccagcccaggtcctggcaaagctcgtagaacgtgaaggtgatcggctcgccgataggggtgcgcttcgcgtactccaacacctgctgccacaccagttcgtcatcgtcggcccgcagctcgacgccggtgtaggtgatcttcacgtccttgttgacgtggaaaatgaccttgttttgcagcgcctcgcgcgggattttcttgttgcgcgtggtgaacagggcagagcgggccgtgtcgtttggcatcgctcgcatcgtgtccggccacggcgcaatatcgaacaaggaaagctgcatttccttgatctgctgcttcgtgtgtttcagcaacgcggcctgcttggcctcgctgacctgttttgccaggtcctcgccggcggtttttcgcttcttggtcgtcatagttcctcgcgtgtcgatggtcatcgacttcgccaaacctgccgcctcctgttcgagacgacgcgaacgctccacggcggccgatggcgcgggcagggcagggggagccagttgcacgctgtcgcgctcgatcttggccgtagcttgctggaccatcgagccgacggactggaaggtttcgcggggcgcacgcatgacggtgcggcttgcgatggtttcggcatcctcggcggaaaaccccgcgtcgatcagttcttgcctgtatgccttccggtcaaacgtccgattcattcaccctccttgcgggattgccccgactcacgccggggcaatgtgcccttattcctgatttgacccgcctggtgccttggtgtccagataatccaccttatcggcaatgaagtcggtcccgtagaccgtctggccgtccttctcgtacttggtattccgaatcttgccctgcacgaataccagctccgcgaagtcgctcttcttgatggagcgcatggggacgtgcttggcaatcacgcgcaccccccggccgttttagcggctaaaaaagtcatggctctgccctcgggcggaccacgcccatcatgaccttgccaagctcgtcctgcttctcttcgatcttcgccagcagggcgaggatcgtggcatcaccgaaccgcgccgtgcgcgggtcgtcggtgagccagagtttcagcaggccgcccaggcggcccaggtcgccattgatgcgggccagctcgcggacgtgctcatagtccacgacgcccgtgattttgtagccctggccgacggccagcaggtaggcctacaggctcatgccggccgccgccgccttttcctcaatcgctcttcgttcgtctggaaggcagtacaccttgataggtgggctgcccttcctggttggcttggtttcatcagccatccgcttgccctcatctgttacgccggcggtagccggccagcctcgcagagcaggattcccgttgagcaccgccaggtgcgaataagggacagtgaagaaggaacacccgctcgcgggtgggcctacttcacctatcctgcccggctgacgccgttggatacaccaaggaaagtctacacgaaccctttggcaaaatcctgtatatcgtgcgaaaaaggatggatataccgaaaaaatcgctataatgaccccgaagcagggttatgcagcggaaaagatccgtc-3´

**pCM130-*P_fdh2_*, the sequences in red represent the promoter *P_fdh2_***

5´-

gaccctttccgacgctcaccgggctggttgccctcgccgctgggctggcggccgtctatggccctgcaaacgcgccagaaacgccgtcgaagccgtgtgcgagacaccgcggccgccggcgttgtggatacctcgcggaaaacttggccctcactgacagatgaggggcggacgttgacacttgaggggccgactcacccggcgcggcgttgacagatgaggggcaggctcgatttcggccggcgacgtggagctggccagcctcgcaaatcggcgaaaacgcctgattttacgcgagtttcccacagatgatgtggacaagcctggggataagtgccctgcggtattgacacttgaggggcgcgactactgacagatgaggggcgcgatccttgacacttgaggggcagagtgctgacagatgaggggcgcacctattgacatttgaggggctgtccacaggcagaaaatccagcatttgcaagggtttccgcccgtttttcggccaccgctaacctgtcttttaacctgcttttaaaccaatatttataaaccttgtttttaaccagggctgcgccctgtgcgcgtgaccgcgcacgccgaaggggggtgcccccccttctcgaaccctcccggcccgctaacgcgggcctcccatccccccaggggctgcgcccctcggccgcgaacggcctcaccccaaaaatggcagccaagctgaccacttctgcgctcggcccttccggctggctggtttattgctgataaatctggagccggtgagcgtgggtctcgcggtatcattgcagcactggggccagatggtaagccctcccgtatcgtagttatctacacgacggggagtcaggcaactatggatgaacgaaatagacagatcgctgagataggtgcctcactgattaagcattggtaactgtcagaccaagtttactcatatatactttagattgatttaaaacttcatttttaatttaaaaggatctaggtgaagatcctttttgataatctcatgaccaaaatcccttaacgtgagttttcgttccactgagcgtcagaccccgtagaaaagatcaaaggatcttcttgagatcctttttttctgcgcgtaatctgctgcttgcaaacaaaaaaaccaccgctaccagcggtggtttgtttgccggatcaagagctaccaactctttttccgaaggtaactggcttcagcagagcgcagataccaaatactgtccttctagtgtagccgtagttaggccaccacttcaagaactctgtagcaccgcctacatacctcgctctgctaatcctgttaccagtggctgctgccagtggcgataagtcgtgtcttaccgggttggactcaagacgatagttaccggataaggcgcagcggtcgggctgaacggggggttcgtgcacacagcccagcttggagcgaacgacctacaccgaactgagatacctacagcgtgagctatgagaaagcgccacgcttcccgaagggagaaaggcggacaggtatccggtaagcggcagggtcggaacaggagagcgcacgagggagcttccagggggaaacgcctggtatctttatagtcctgtcgggtttcgccacctctgacttgagcgtcgatttttgtgatgctcgtcaggggggcggagcctatggaaaaacgccagcaacgcggcctttttacggttcctggccttttgctggccttttgctcacatggtacccgggagctccatcaggtgagcacggtcatgaatcgttcgttgagaatgcggtcgtggtaaaagatcgccttgcccagctggtcggtggtccaggtcaccggtttgtggtccgggtagttgtaatctcccccgcagaacacttcgttgcggttaccggacgggtcgaagaagtagatggtcttgccgtgagtgaggccgtggcgggttgggccgatatcgatagatgtgtcggtcatggagatcaggtcggcggcgcgaagcaagtcttcccaggtttcgaggtggaaggacacatgatggaggcggcctttttccggatggtgaatgaaggccacgtcgtgggccttggtcgacagactgagaaactgggcgacgcgcgtgccattttcgtccagcacctgttcggccagatagaaaccgagcaccttggtgaacaggtcataggtcgccggcaattcgtcgccatacatgagggcgtggtcgaaacgcacagccgccatacctttcagatcgcgcggccatgcctcgggattgacgtcattcaaaccccactttccagtatattccttgtctgcatacaactcgaagtgatgcccggagggggcctggaagcgcacgcgccggccacaactgttcagttcacctgcgggtagctgctcaacggcacagccatatgccatcagatcccgctccagttgccggagagcatcctcatccacaaccttgaaacccataaaatccatgcccggctcgtcagcctcgcgtagcaccagggaaaacttatccacttcggtccaagccttcagatagacacggccctggtcgtcacggtccatctcgatcaggcccagcaactcgacgtagtgttccagggccttgctcatgtccagtacacgcagctgcacatggcccggtcgcattacacctttgttcatgacgtcacctcctgaagcttCGTTTCCTGCCCAACTTGGTTTTGTTCTAAGGCCGGATCCAAACAGGGCCGGCACTAAAGTTCAATCAAGCAGTCTCGTGGTTTGACAAAAATCTTCGATCAAAACGAGTCAGTTGCAGTGCATCATCCGCTCAACGGCGGTTCAAACTTTTTTTGAAGCGGCGAAAAATTTTCTTCGAGGCCATCGGGggatccccgggtaccgagctcgaatctagaattccctgctttcctgatgcaaaaacgaggctagtttaccgtatctgtggggggatggcttgtagatatgacgacaggaagagtttgtagaaacgcaaaaaggccatccgtcaggatggccttctgcttaatttgatgcctggcagtttatggcgggcgtcctgcccgccaccctccgggccgttgcttcgcaacgttcaaatccgctcccggcggatttgtcctactcaggagagcgttcaccgacaaacaacagataaaacgaaaggcccagtctttcgactgagcctttcgttttatttgatgcctggcagttccctactctcgcatggggagaccccacactaccatcggcgctacggcgtttcacttctgagttcggcatggggtcaggtgggaccaccgcgctactgccgccaggcaaattctgttttatcagaccgcttctgcgttctgatttaatctgtatcaggctgaaaaattcactggccgtcgttttacaacgtcgtgactgggaaaaccctggcgttacccaacttaatcgccttgcagcacatccccctttcgccagctggcgtaatagcgaagaggcccgcaccgatcgcccttcccaacagttgcgcagcctgaatggcgaatggcgcctgatgcggtattttctccttacgcatctgtgcggtatttcacaccgcatatggtgcactctcagtacaatctgctctgatgccgcatagttaagccagccccgacacccgccaacacccgctgacgcgccctgacgggcttgtctgctcccggcatccgcttacagacaagctgtgaccgtctccgggagctgcatgtgtcagaggttttcaccgtcatcaccgaaacgcgcgagacgaaagggcctcgtgatacgcctatttttataggttaatgtcatgataataatggtttcttagcaccctttctcggtccttcaacgttcctgacaacgagcctccttttcgccaatccatcgacaatcaccgcgagtccctgctcgaacgctgcgtccggaccggcttcgtcgaaggcgtctatcgcggcccgcaacagcggcgagagcggagcctgttcaacggtgccgccgcgctcgccggcatcgctgtcgccggcctgctcctcaagcacggccccaacagtgaagtagctgattgtcatcagcgcattgacggcgtccccggccgaaaaacccgcctcgcagaggaagcgaagctgcgcgtcggccgtttccatctgcggtgcgcccggtcgcgtgccggcatggatgcgcgcgccatcgcggtaggcgagcagcgcctgcctgaagctgcgggcattcccgatcagaaatgagcgccagtcgtcgtcggctctcggcaccgaatgcgtatgattctccgccagcatggcttcggccagtgcgtcgagcagcgcccgcttgttcctgaagtgccagtaaagcgccggctgctgaacccccaaccgttccgccagtttgcgtgtcgtcagaccgtctacgccgacctcgttcaacaggtccagggcggcacggatcactgtattcggctgcaactttgtcatgattgacactttatcactgataaacataatatgtccaccaacttatcagtgataaagaatccgcgcgttcaatcggaccagcggaggctggtccggaggccagacgtgaaacccaacatacccctgatcgtaattctgagcactgtcgcgctcgacgctgtcggcatcggcctgattatgccggtgctgccgggcctcctgcgcgatctggttcactcgaacgacgtcaccgcccactatggcattctgctggcgctgtatgcgttggtgcaatttgcctgcgcacctgtgctgggcgcgctgtcggatcgtttcgggcggcggccaatcttgctcgtctcgctggccggcgccactgtcgactacgccatcatggcgacagcgcctttcctttgggttctctatatcgggcggatcgtggccggcatcaccggggcgactggggcggtagccggcgcttatattgccgatatcactgatggcgatgagcgcgcgcggcacttcggcttcatgagcgcctgtttcgggttcgggatggtcgcgggacctgtgctcggtgggctgatgggcggtttctccccccacgctccgttcttcgccgcggcagccttgaacggcctcaatttcctgacgggctgtttccttttgccggagtcgcacaaaggcgaacgccggccgttacgccgggaggctctcaacccgctcgcttcgttccggtgggcccggggcatgaccgtcgtcgccgccctgatggcggtcttcttcatcatgcaacttgtcggacaggtgccggccgcgctttgggtcattttcggcgaggatcgctttcactgggacgcgaccacgatcggcatttcgcttgccgcatttggcattctgcattcactcgcccaggcaatgatcaccggccctgtagccgcccggctcggcgaaaggcgggcactcatgctcggaatgattgccgacggcacaggctacatcctgcttgccttcgcgacacggggatggatggcgttcccgatcatggtcctgcttgcttcgggtggcatcggaatgccggcgctgcaagcaatgttgtccaggcaggtggatgaggaacgtcaggggcagctgcaaggctcactggcggcgctcaccagcctgacctcgatcgtcggacccctcctcttcacggcgatctatgcggcttctataacaacgtggaacgggtgggcatggattgcaggcgctgccctctacttgctctgcctgccggcgctgcgtcgcgggctttggagcggcgcagggcaacgagccgatcgctgatcgtggaaacgataggcctatgccatgcgggtcaaggcgacttccggcaagctatacgcgccctagaattgtcaattttaatcctctgtttatcggcagttcgtagagcgcgccgtgcgtcccgagcgatactgagcgaagcaagtgcgtcgagcagtgcccgcttgttcctgaaatgccagtaaagcgctggctgctgaacccccagccggaactgaccccacaaggccctagcgtttgcaatgcaccaggtcatcattgacccaggcgtgttccaccaggccgctgcctcgcaactcttcgcaggcttcgccgacctgctcgcgccacttcttcacgcgggtggaatccgatccgcacatgaggcggaaggtttccagcttgagcgggtacggctcccggtgcgagctgaaatagtcgaacatccgtcgggccgtcggcgacagcttgcggtacttctcccatatgaatttcgtgtagtggtcgccagcaaacagcacgacgatttcctcgtcgatcaggacctggcaacgggacgttttcttgccacggtccaggacgcggaagcggtgcagcagcgacaccgattccaggtgcccaacgcggtcggacgtgaagcccatcgccgtcgcctgtaggcgcgacaggcattcctcggccttcgtgtaataccggccattgatcgaccagcccaggtcctggcaaagctcgtagaacgtgaaggtgatcggctcgccgataggggtgcgcttcgcgtactccaacacctgctgccacaccagttcgtcatcgtcggcccgcagctcgacgccggtgtaggtgatcttcacgtccttgttgacgtggaaaatgaccttgttttgcagcgcctcgcgcgggattttcttgttgcgcgtggtgaacagggcagagcgggccgtgtcgtttggcatcgctcgcatcgtgtccggccacggcgcaatatcgaacaaggaaagctgcatttccttgatctgctgcttcgtgtgtttcagcaacgcggcctgcttggcctcgctgacctgttttgccaggtcctcgccggcggtttttcgcttcttggtcgtcatagttcctcgcgtgtcgatggtcatcgacttcgccaaacctgccgcctcctgttcgagacgacgcgaacgctccacggcggccgatggcgcgggcagggcagggggagccagttgcacgctgtcgcgctcgatcttggccgtagcttgctggaccatcgagccgacggactggaaggtttcgcggggcgcacgcatgacggtgcggcttgcgatggtttcggcatcctcggcggaaaaccccgcgtcgatcagttcttgcctgtatgccttccggtcaaacgtccgattcattcaccctccttgcgggattgccccgactcacgccggggcaatgtgcccttattcctgatttgacccgcctggtgccttggtgtccagataatccaccttatcggcaatgaagtcggtcccgtagaccgtctggccgtccttctcgtacttggtattccgaatcttgccctgcacgaataccagctccgcgaagtcgctcttcttgatggagcgcatggggacgtgcttggcaatcacgcgcaccccccggccgttttagcggctaaaaaagtcatggctctgccctcgggcggaccacgcccatcatgaccttgccaagctcgtcctgcttctcttcgatcttcgccagcagggcgaggatcgtggcatcaccgaaccgcgccgtgcgcgggtcgtcggtgagccagagtttcagcaggccgcccaggcggcccaggtcgccattgatgcgggccagctcgcggacgtgctcatagtccacgacgcccgtgattttgtagccctggccgacggccagcaggtaggcctacaggctcatgccggccgccgccgccttttcctcaatcgctcttcgttcgtctggaaggcagtacaccttgataggtgggctgcccttcctggttggcttggtttcatcagccatccgcttgccctcatctgttacgccggcggtagccggccagcctcgcagagcaggattcccgttgagcaccgccaggtgcgaataagggacagtgaagaaggaacacccgctcgcgggtgggcctacttcacctatcctgcccggctgacgccgttggatacaccaaggaaagtctacacgaaccctttggcaaaatcctgtatatcgtgcgaaaaaggatggatataccgaaaaaatcgctataatgaccccgaagcagggttatgcagcggaaaagatccgtc-3´

**pCM80-0287, the sequences in red encode the gene *META1_0287***

5´-

gaccctttccgacgctcaccgggctggttgccctcgccgctgggctggcggccgtctatggccctgcaaacgcgccagaaacgccgtcgaagccgtgtgcgagacaccgcggccgccggcgttgtggatacctcgcggaaaacttggccctcactgacagatgaggggcggacgttgacacttgaggggccgactcacccggcgcggcgttgacagatgaggggcaggctcgatttcggccggcgacgtggagctggccagcctcgcaaatcggcgaaaacgcctgattttacgcgagtttcccacagatgatgtggacaagcctggggataagtgccctgcggtattgacacttgaggggcgcgactactgacagatgaggggcgcgatccttgacacttgaggggcagagtgctgacagatgaggggcgcacctattgacatttgaggggctgtccacaggcagaaaatccagcatttgcaagggtttccgcccgtttttcggccaccgctaacctgtcttttaacctgcttttaaaccaatatttataaaccttgtttttaaccagggctgcgccctgtgcgcgtgaccgcgcacgccgaaggggggtgcccccccttctcgaaccctcccggcccgctaacgcgggcctcccatccccccaggggctgcgcccctcggccgcgaacggcctcaccccaaaaatggcagccaagctgaccacttctgcgctcggcccttccggctggctggtttattgctgataaatctggagccggtgagcgtgggtctcgcggtatcattgcagcactggggccagatggtaagccctcccgtatcgtagttatctacacgacggggagtcaggcaactatggatgaacgaaatagacagatcgctgagataggtgcctcactgattaagcattggtaactgtcagaccaagtttactcatatatactttagattgatttaaaacttcatttttaatttaaaaggatctaggtgaagatcctttttgataatctcatgaccaaaatcccttaacgtgagttttcgttccactgagcgtcagaccccgtagaaaagatcaaaggatcttcttgagatcctttttttctgcgcgtaatctgctgcttgcaaacaaaaaaaccaccgctaccagcggtggtttgtttgccggatcaagagctaccaactctttttccgaaggtaactggcttcagcagagcgcagataccaaatactgtccttctagtgtagccgtagttaggccaccacttcaagaactctgtagcaccgcctacatacctcgctctgctaatcctgttaccagtggctgctgccagtggcgataagtcgtgtcttaccgggttggactcaagacgatagttaccggataaggcgcagcggtcgggctgaacggggggttcgtgcacacagcccagcttggagcgaacgacctacaccgaactgagatacctacagcgtgagctatgagaaagcgccacgcttcccgaagggagaaaggcggacaggtatccggtaagcggcagggtcggaacaggagagcgcacgagggagcttccagggggaaacgcctggtatctttatagtcctgtcgggtttcgccacctctgacttgagcgtcgatttttgtgatgctcgtcaggggggcggagcctatggaaaaacgccagcaacgcggcctttttacggttcctggccttttgctggccttttgctcacatgttctttcctgcgttatcccctgattctgtggataaccgtattaccgcctttgagtgagctgataccgctcgccgcagccgaacgaccgagcgcagcgagtcagtgagcgaggaagcggaagagcgcccaatacgcaaaccgcctctccccgcgcgttggccgattcattaatgcagctggcacgacaggtttcccgactggaaagcgggcagtgagcgcaacgcaattaatgtgagttagctcactcattaggcaccccaggctttacactttatgcttccggctcgtatgttgtgtggaattgtgagcggataacaatttcacacaggaaacagctatgaccatgattacgccaagctagcttcccgcttggtcgggccgcttcgcgagggcccgttgacgacaacggtgcgatgggtcccggccccggtcaagacgatgccaatacgttgcgacactacgccttggcacttttagaattgccttatcgtcctgataagaaatgtccgaccagctaaagacatcgcgtccaatcaaagcctagaaaatataggcgaagggacgctaataagtctttcataagaccgcgcaaatctaaaaatatccttagattcacgatgcggcacttcggatgacttccgagcgagcctggaacctcagaaaaacgtctgagagataccgcggatctcacacaggaaacagctatgaccatgattacgccaagcttTTGGGCTGCGACGAGACGACCGCCGAGGGCCGCATGTCCCAGGAATCCCGTACGCACGGAGACCCGGAAGACGAGGGTGAAGCGAAGACCCACGGCGCCCATACCCGCGAGAAGGACAACGCCCAGGTCGAGAAGGCCCACCGCCCGGACGCGCTGATCCTGCACGAGATCATCCGGCGGGAAGGCGAGGAGGAGATGCGCCGCACTTGGCTGGCCCTCTCGCTCTCGGGGTTCGCCGCCGGGCTCACCATGGGCTTCTCGCTGATCGTGCCGGGCGTGCTCAAGGGGCATCTGCCGCACGCGCCCTGGGCCGAACTCGTCACCAGCGCGGGCTATTCGATCGGCTTCCTCATCGTTGTGCTCGGGCGCCAGCAACTCTTCACCGAGAACACGGTCACGCCGATCCTCCCGCTCCTCACCGAGAGAACCTTCGGTGCGTTGCTGCGGGTCGTGCGGCTCTGGGGCATCGTGCTCGTCGCCAACATCCTGGCGACGATCGCGATCGCCTCGGTGCTGGCTCATACCGATGCGTTCAAGCCTGAGGTGCGGGAGGCTTTTGCCGAGATCAGCCGCCACACCATCGAGGACCCGTTCTGGACCACGGTGATCAAGGCGGTCTTCGCAGGCTGGCTGATCGCCCTGATGGTGTGGATCCTGCCGGCCTCGGGCTCGGCGGCGCCCTTCATCATCATCCTGATGACGTGGCTGGTCTCGATGTGCGGGCTCGCCCACATCGTCGCCGGCTCAGTCGATGCCTATTACCTCGTCGCCATCGGCGAGATCGATTTTTCGAAGTACCTCACCGGGTTCTTCGTCCCCACGCTGCTCGGCAACATCGTCGGCGGCGTGACGCTCGTCTCGGTGCTCAATTTCGGGCAGGTGGCGCCCGAGATCGAGGATCACAATCGCGTCGGCGCCTGAggatccccgggtaccgagctcgaattcactggccgtcgttttacaacgtcgtgactgggaaaaccctggcgttacccaacttaatcgccttgcagcacatccccctttcgccagctggcgtaatagcgaagaggcccgcaccgatcgcccttcccaacagttgcgcagcctgaatggcgaatggcgcctgatgcggtattttctccttacgcatctgtgcggtatttcacaccgcatatggtgcactctcagtacaatctgctctgatgccgcatagttaagccagccccgacacccgccaacacccgctgacgcgccctgacgggcttgtctgctcccggcatccgcttacagacaagctgtgaccgtctccgggagctgcatgtgtcagaggttttcaccgtcatcaccgaaacgcgcgagacgaaagggcctcgtgatacgcctatttttataggttaatgtcatgataataatggtttcttagcaccctttctcggtccttcaacgttcctgacaacgagcctccttttcgccaatccatcgacaatcaccgcgagtccctgctcgaacgctgcgtccggaccggcttcgtcgaaggcgtctatcgcggcccgcaacagcggcgagagcggagcctgttcaacggtgccgccgcgctcgccggcatcgctgtcgccggcctgctcctcaagcacggccccaacagtgaagtagctgattgtcatcagcgcattgacggcgtccccggccgaaaaacccgcctcgcagaggaagcgaagctgcgcgtcggccgtttccatctgcggtgcgcccggtcgcgtgccggcatggatgcgcgcgccatcgcggtaggcgagcagcgcctgcctgaagctgcgggcattcccgatcagaaatgagcgccagtcgtcgtcggctctcggcaccgaatgcgtatgattctccgccagcatggcttcggccagtgcgtcgagcagcgcccgcttgttcctgaagtgccagtaaagcgccggctgctgaacccccaaccgttccgccagtttgcgtgtcgtcagaccgtctacgccgacctcgttcaacaggtccagggcggcacggatcactgtattcggctgcaactttgtcatgattgacactttatcactgataaacataatatgtccaccaacttatcagtgataaagaatccgcgcgttcaatcggaccagcggaggctggtccggaggccagacgtgaaacccaacatacccctgatcgtaattctgagcactgtcgcgctcgacgctgtcggcatcggcctgattatgccggtgctgccgggcctcctgcgcgatctggttcactcgaacgacgtcaccgcccactatggcattctgctggcgctgtatgcgttggtgcaatttgcctgcgcacctgtgctgggcgcgctgtcggatcgtttcgggcggcggccaatcttgctcgtctcgctggccggcgccactgtcgactacgccatcatggcgacagcgcctttcctttgggttctctatatcgggcggatcgtggccggcatcaccggggcgactggggcggtagccggcgcttatattgccgatatcactgatggcgatgagcgcgcgcggcacttcggcttcatgagcgcctgtttcgggttcgggatggtcgcgggacctgtgctcggtgggctgatgggcggtttctccccccacgctccgttcttcgccgcggcagccttgaacggcctcaatttcctgacgggctgtttccttttgccggagtcgcacaaaggcgaacgccggccgttacgccgggaggctctcaacccgctcgcttcgttccggtgggcccggggcatgaccgtcgtcgccgccctgatggcggtcttcttcatcatgcaacttgtcggacaggtgccggccgcgctttgggtcattttcggcgaggatcgctttcactgggacgcgaccacgatcggcatttcgcttgccgcatttggcattctgcattcactcgcccaggcaatgatcaccggccctgtagccgcccggctcggcgaaaggcgggcactcatgctcggaatgattgccgacggcacaggctacatcctgcttgccttcgcgacacggggatggatggcgttcccgatcatggtcctgcttgcttcgggtggcatcggaatgccggcgctgcaagcaatgttgtccaggcaggtggatgaggaacgtcaggggcagctgcaaggctcactggcggcgctcaccagcctgacctcgatcgtcggacccctcctcttcacggcgatctatgcggcttctataacaacgtggaacgggtgggcatggattgcaggcgctgccctctacttgctctgcctgccggcgctgcgtcgcgggctttggagcggcgcagggcaacgagccgatcgctgatcgtggaaacgataggcctatgccatgcgggtcaaggcgacttccggcaagctatacgcgccctagaattgtcaattttaatcctctgtttatcggcagttcgtagagcgcgccgtgcgtcccgagcgatactgagcgaagcaagtgcgtcgagcagtgcccgcttgttcctgaaatgccagtaaagcgctggctgctgaacccccagccggaactgaccccacaaggccctagcgtttgcaatgcaccaggtcatcattgacccaggcgtgttccaccaggccgctgcctcgcaactcttcgcaggcttcgccgacctgctcgcgccacttcttcacgcgggtggaatccgatccgcacatgaggcggaaggtttccagcttgagcgggtacggctcccggtgcgagctgaaatagtcgaacatccgtcgggccgtcggcgacagcttgcggtacttctcccatatgaatttcgtgtagtggtcgccagcaaacagcacgacgatttcctcgtcgatcaggacctggcaacgggacgttttcttgccacggtccaggacgcggaagcggtgcagcagcgacaccgattccaggtgcccaacgcggtcggacgtgaagcccatcgccgtcgcctgtaggcgcgacaggcattcctcggccttcgtgtaataccggccattgatcgaccagcccaggtcctggcaaagctcgtagaacgtgaaggtgatcggctcgccgataggggtgcgcttcgcgtactccaacacctgctgccacaccagttcgtcatcgtcggcccgcagctcgacgccggtgtaggtgatcttcacgtccttgttgacgtggaaaatgaccttgttttgcagcgcctcgcgcgggattttcttgttgcgcgtggtgaacagggcagagcgggccgtgtcgtttggcatcgctcgcatcgtgtccggccacggcgcaatatcgaacaaggaaagctgcatttccttgatctgctgcttcgtgtgtttcagcaacgcggcctgcttggcctcgctgacctgttttgccaggtcctcgccggcggtttttcgcttcttggtcgtcatagttcctcgcgtgtcgatggtcatcgacttcgccaaacctgccgcctcctgttcgagacgacgcgaacgctccacggcggccgatggcgcgggcagggcagggggagccagttgcacgctgtcgcgctcgatcttggccgtagcttgctggaccatcgagccgacggactggaaggtttcgcggggcgcacgcatgacggtgcggcttgcgatggtttcggcatcctcggcggaaaaccccgcgtcgatcagttcttgcctgtatgccttccggtcaaacgtccgattcattcaccctccttgcgggattgccccgactcacgccggggcaatgtgcccttattcctgatttgacccgcctggtgccttggtgtccagataatccaccttatcggcaatgaagtcggtcccgtagaccgtctggccgtccttctcgtacttggtattccgaatcttgccctgcacgaataccagctccgcgaagtcgctcttcttgatggagcgcatggggacgtgcttggcaatcacgcgcaccccccggccgttttagcggctaaaaaagtcatggctctgccctcgggcggaccacgcccatcatgaccttgccaagctcgtcctgcttctcttcgatcttcgccagcagggcgaggatcgtggcatcaccgaaccgcgccgtgcgcgggtcgtcggtgagccagagtttcagcaggccgcccaggcggcccaggtcgccattgatgcgggccagctcgcggacgtgctcatagtccacgacgcccgtgattttgtagccctggccgacggccagcaggtaggcctacaggctcatgccggccgccgccgccttttcctcaatcgctcttcgttcgtctggaaggcagtacaccttgataggtgggctgcccttcctggttggcttggtttcatcagccatccgcttgccctcatctgttacgccggcggtagccggccagcctcgcagagcaggattcccgttgagcaccgccaggtgcgaataagggacagtgaagaaggaacacccgctcgcgggtgggcctacttcacctatcctgcccggctgacgccgttggatacaccaaggaaagtctacacgaaccctttggcaaaatcctgtatatcgtgcgaaaaaggatggatataccgaaaaaatcgctataatgaccccgaagcagggttatgcagcggaaaagatccgtc-3´

**pCM80-0287*, the sequences in red encode the gene *META1_0287****

5´-

gaccctttccgacgctcaccgggctggttgccctcgccgctgggctggcggccgtctatggccctgcaaacgcgccagaaacgccgtcgaagccgtgtgcgagacaccgcggccgccggcgttgtggatacctcgcggaaaacttggccctcactgacagatgaggggcggacgttgacacttgaggggccgactcacccggcgcggcgttgacagatgaggggcaggctcgatttcggccggcgacgtggagctggccagcctcgcaaatcggcgaaaacgcctgattttacgcgagtttcccacagatgatgtggacaagcctggggataagtgccctgcggtattgacacttgaggggcgcgactactgacagatgaggggcgcgatccttgacacttgaggggcagagtgctgacagatgaggggcgcacctattgacatttgaggggctgtccacaggcagaaaatccagcatttgcaagggtttccgcccgtttttcggccaccgctaacctgtcttttaacctgcttttaaaccaatatttataaaccttgtttttaaccagggctgcgccctgtgcgcgtgaccgcgcacgccgaaggggggtgcccccccttctcgaaccctcccggcccgctaacgcgggcctcccatccccccaggggctgcgcccctcggccgcgaacggcctcaccccaaaaatggcagccaagctgaccacttctgcgctcggcccttccggctggctggtttattgctgataaatctggagccggtgagcgtgggtctcgcggtatcattgcagcactggggccagatggtaagccctcccgtatcgtagttatctacacgacggggagtcaggcaactatggatgaacgaaatagacagatcgctgagataggtgcctcactgattaagcattggtaactgtcagaccaagtttactcatatatactttagattgatttaaaacttcatttttaatttaaaaggatctaggtgaagatcctttttgataatctcatgaccaaaatcccttaacgtgagttttcgttccactgagcgtcagaccccgtagaaaagatcaaaggatcttcttgagatcctttttttctgcgcgtaatctgctgcttgcaaacaaaaaaaccaccgctaccagcggtggtttgtttgccggatcaagagctaccaactctttttccgaaggtaactggcttcagcagagcgcagataccaaatactgtccttctagtgtagccgtagttaggccaccacttcaagaactctgtagcaccgcctacatacctcgctctgctaatcctgttaccagtggctgctgccagtggcgataagtcgtgtcttaccgggttggactcaagacgatagttaccggataaggcgcagcggtcgggctgaacggggggttcgtgcacacagcccagcttggagcgaacgacctacaccgaactgagatacctacagcgtgagctatgagaaagcgccacgcttcccgaagggagaaaggcggacaggtatccggtaagcggcagggtcggaacaggagagcgcacgagggagcttccagggggaaacgcctggtatctttatagtcctgtcgggtttcgccacctctgacttgagcgtcgatttttgtgatgctcgtcaggggggcggagcctatggaaaaacgccagcaacgcggcctttttacggttcctggccttttgctggccttttgctcacatgttctttcctgcgttatcccctgattctgtggataaccgtattaccgcctttgagtgagctgataccgctcgccgcagccgaacgaccgagcgcagcgagtcagtgagcgaggaagcggaagagcgcccaatacgcaaaccgcctctccccgcgcgttggccgattcattaatgcagctggcacgacaggtttcccgactggaaagcgggcagtgagcgcaacgcaattaatgtgagttagctcactcattaggcaccccaggctttacactttatgcttccggctcgtatgttgtgtggaattgtgagcggataacaatttcacacaggaaacagctatgaccatgattacgccaagctagcttcccgcttggtcgggccgcttcgcgagggcccgttgacgacaacggtgcgatgggtcccggccccggtcaagacgatgccaatacgttgcgacactacgccttggcacttttagaattgccttatcgtcctgataagaaatgtccgaccagctaaagacatcgcgtccaatcaaagcctagaaaatataggcgaagggacgctaataagtctttcataagaccgcgcaaatctaaaaatatccttagattcacgatgcggcacttcggatgacttccgagcgagcctggaacctcagaaaaacgtctgagagataccgcggatctcacacaggaaacagctatgaccatgattacgccaagcttTTGGGCTGCGACGAGACGACCGCCGAGGGCCGCATGTCCCAGcAATCCCGTACGCACGGAGACCCGGAAGACGAGGGTGAAGCGAAGACCCACGGCGCCCATACCCGCGAGAAGGACAACGCCCAGGTCGAGAAGGCCCACCGCCCGGACGCGCTGATCCTGCACGAGATCATCCGGCGGGAAGGCGAGGAGGAGATGCGCCGCACTTGGCTGGCCCTCTCGCTCTCGGGGTTCGCCGCCGGGCTCACCATGGGCTTCTCGCTGATCGTGCCGGGCGTGCTCAAGGGGCATCTGCCGCACGCGCCCTGGGCCGAACTCGTCACCAGCGCGGGCTATTCGATCGGCTTCCTCATCGTTGTGCTCGGGCGCCAGCAACTCTTCACCGAGAACACGGTCACGCCGATCCTCCCGCTCCTCACCGAGAGAACCTTCGGTGCGTTGCTGCGGGTCGTGCGGCTCTGGGGCATCGTGCTCGTCGCCAACATCCTGGCGACGATCGCGATCGCCTCGGTGCTGGCTCATACCGATGCGTTCAAGCCTGAGGTGCGGGAGGCTTTTGCCGAGATCAGCCGCCACACCATCGAGGACCCGTTCTGGACCACGGTGATCAAGGCGGTCTTCGCAGGCTGGCTGATCGCCCTGATGGTGTGGATCCTGCCGGCCTCGGGCTCGGCGGCGCCCTTCATCATCATCCTGATGACGTGGCTGGTCTCGATGTGCGGGCTCGCCCACATCGTCGCCGGCTCAGTCGATGCCTATTACCTCGTCGCCATCGGCGAGATCGATTTTTCGAAGTACCTCACCGGGTTCTTCGTCCCCACGCTGCTCGGCAACATCGTCGGCGGCGTGACGCTCGTCTCGGTGCTCAATTTCGGGCAGGTGGCGCCCGAGATCGAGGATCACAATCGCGTCGGCGCCTGAggatccccgggtaccgagctcgaattcactggccgtcgttttacaacgtcgtgactgggaaaaccctggcgttacccaacttaatcgccttgcagcacatccccctttcgccagctggcgtaatagcgaagaggcccgcaccgatcgcccttcccaacagttgcgcagcctgaatggcgaatggcgcctgatgcggtattttctccttacgcatctgtgcggtatttcacaccgcatatggtgcactctcagtacaatctgctctgatgccgcatagttaagccagccccgacacccgccaacacccgctgacgcgccctgacgggcttgtctgctcccggcatccgcttacagacaagctgtgaccgtctccgggagctgcatgtgtcagaggttttcaccgtcatcaccgaaacgcgcgagacgaaagggcctcgtgatacgcctatttttataggttaatgtcatgataataatggtttcttagcaccctttctcggtccttcaacgttcctgacaacgagcctccttttcgccaatccatcgacaatcaccgcgagtccctgctcgaacgctgcgtccggaccggcttcgtcgaaggcgtctatcgcggcccgcaacagcggcgagagcggagcctgttcaacggtgccgccgcgctcgccggcatcgctgtcgccggcctgctcctcaagcacggccccaacagtgaagtagctgattgtcatcagcgcattgacggcgtccccggccgaaaaacccgcctcgcagaggaagcgaagctgcgcgtcggccgtttccatctgcggtgcgcccggtcgcgtgccggcatggatgcgcgcgccatcgcggtaggcgagcagcgcctgcctgaagctgcgggcattcccgatcagaaatgagcgccagtcgtcgtcggctctcggcaccgaatgcgtatgattctccgccagcatggcttcggccagtgcgtcgagcagcgcccgcttgttcctgaagtgccagtaaagcgccggctgctgaacccccaaccgttccgccagtttgcgtgtcgtcagaccgtctacgccgacctcgttcaacaggtccagggcggcacggatcactgtattcggctgcaactttgtcatgattgacactttatcactgataaacataatatgtccaccaacttatcagtgataaagaatccgcgcgttcaatcggaccagcggaggctggtccggaggccagacgtgaaacccaacatacccctgatcgtaattctgagcactgtcgcgctcgacgctgtcggcatcggcctgattatgccggtgctgccgggcctcctgcgcgatctggttcactcgaacgacgtcaccgcccactatggcattctgctggcgctgtatgcgttggtgcaatttgcctgcgcacctgtgctgggcgcgctgtcggatcgtttcgggcggcggccaatcttgctcgtctcgctggccggcgccactgtcgactacgccatcatggcgacagcgcctttcctttgggttctctatatcgggcggatcgtggccggcatcaccggggcgactggggcggtagccggcgcttatattgccgatatcactgatggcgatgagcgcgcgcggcacttcggcttcatgagcgcctgtttcgggttcgggatggtcgcgggacctgtgctcggtgggctgatgggcggtttctccccccacgctccgttcttcgccgcggcagccttgaacggcctcaatttcctgacgggctgtttccttttgccggagtcgcacaaaggcgaacgccggccgttacgccgggaggctctcaacccgctcgcttcgttccggtgggcccggggcatgaccgtcgtcgccgccctgatggcggtcttcttcatcatgcaacttgtcggacaggtgccggccgcgctttgggtcattttcggcgaggatcgctttcactgggacgcgaccacgatcggcatttcgcttgccgcatttggcattctgcattcactcgcccaggcaatgatcaccggccctgtagccgcccggctcggcgaaaggcgggcactcatgctcggaatgattgccgacggcacaggctacatcctgcttgccttcgcgacacggggatggatggcgttcccgatcatggtcctgcttgcttcgggtggcatcggaatgccggcgctgcaagcaatgttgtccaggcaggtggatgaggaacgtcaggggcagctgcaaggctcactggcggcgctcaccagcctgacctcgatcgtcggacccctcctcttcacggcgatctatgcggcttctataacaacgtggaacgggtgggcatggattgcaggcgctgccctctacttgctctgcctgccggcgctgcgtcgcgggctttggagcggcgcagggcaacgagccgatcgctgatcgtggaaacgataggcctatgccatgcgggtcaaggcgacttccggcaagctatacgcgccctagaattgtcaattttaatcctctgtttatcggcagttcgtagagcgcgccgtgcgtcccgagcgatactgagcgaagcaagtgcgtcgagcagtgcccgcttgttcctgaaatgccagtaaagcgctggctgctgaacccccagccggaactgaccccacaaggccctagcgtttgcaatgcaccaggtcatcattgacccaggcgtgttccaccaggccgctgcctcgcaactcttcgcaggcttcgccgacctgctcgcgccacttcttcacgcgggtggaatccgatccgcacatgaggcggaaggtttccagcttgagcgggtacggctcccggtgcgagctgaaatagtcgaacatccgtcgggccgtcggcgacagcttgcggtacttctcccatatgaatttcgtgtagtggtcgccagcaaacagcacgacgatttcctcgtcgatcaggacctggcaacgggacgttttcttgccacggtccaggacgcggaagcggtgcagcagcgacaccgattccaggtgcccaacgcggtcggacgtgaagcccatcgccgtcgcctgtaggcgcgacaggcattcctcggccttcgtgtaataccggccattgatcgaccagcccaggtcctggcaaagctcgtagaacgtgaaggtgatcggctcgccgataggggtgcgcttcgcgtactccaacacctgctgccacaccagttcgtcatcgtcggcccgcagctcgacgccggtgtaggtgatcttcacgtccttgttgacgtggaaaatgaccttgttttgcagcgcctcgcgcgggattttcttgttgcgcgtggtgaacagggcagagcgggccgtgtcgtttggcatcgctcgcatcgtgtccggccacggcgcaatatcgaacaaggaaagctgcatttccttgatctgctgcttcgtgtgtttcagcaacgcggcctgcttggcctcgctgacctgttttgccaggtcctcgccggcggtttttcgcttcttggtcgtcatagttcctcgcgtgtcgatggtcatcgacttcgccaaacctgccgcctcctgttcgagacgacgcgaacgctccacggcggccgatggcgcgggcagggcagggggagccagttgcacgctgtcgcgctcgatcttggccgtagcttgctggaccatcgagccgacggactggaaggtttcgcggggcgcacgcatgacggtgcggcttgcgatggtttcggcatcctcggcggaaaaccccgcgtcgatcagttcttgcctgtatgccttccggtcaaacgtccgattcattcaccctccttgcgggattgccccgactcacgccggggcaatgtgcccttattcctgatttgacccgcctggtgccttggtgtccagataatccaccttatcggcaatgaagtcggtcccgtagaccgtctggccgtccttctcgtacttggtattccgaatcttgccctgcacgaataccagctccgcgaagtcgctcttcttgatggagcgcatggggacgtgcttggcaatcacgcgcaccccccggccgttttagcggctaaaaaagtcatggctctgccctcgggcggaccacgcccatcatgaccttgccaagctcgtcctgcttctcttcgatcttcgccagcagggcgaggatcgtggcatcaccgaaccgcgccgtgcgcgggtcgtcggtgagccagagtttcagcaggccgcccaggcggcccaggtcgccattgatgcgggccagctcgcggacgtgctcatagtccacgacgcccgtgattttgtagccctggccgacggccagcaggtaggcctacaggctcatgccggccgccgccgccttttcctcaatcgctcttcgttcgtctggaaggcagtacaccttgataggtgggctgcccttcctggttggcttggtttcatcagccatccgcttgccctcatctgttacgccggcggtagccggccagcctcgcagagcaggattcccgttgagcaccgccaggtgcgaataagggacagtgaagaaggaacacccgctcgcgggtgggcctacttcacctatcctgcccggctgacgccgttggatacaccaaggaaagtctacacgaaccctttggcaaaatcctgtatatcgtgcgaaaaaggatggatataccgaaaaaatcgctataatgaccccgaagcagggttatgcagcggaaaagatccgtc-3´

**pCM80-2965, the sequences in red encode the gene *META1_2965***

5´-

gaccctttccgacgctcaccgggctggttgccctcgccgctgggctggcggccgtctatggccctgcaaacgcgccagaaacgccgtcgaagccgtgtgcgagacaccgcggccgccggcgttgtggatacctcgcggaaaacttggccctcactgacagatgaggggcggacgttgacacttgaggggccgactcacccggcgcggcgttgacagatgaggggcaggctcgatttcggccggcgacgtggagctggccagcctcgcaaatcggcgaaaacgcctgattttacgcgagtttcccacagatgatgtggacaagcctggggataagtgccctgcggtattgacacttgaggggcgcgactactgacagatgaggggcgcgatccttgacacttgaggggcagagtgctgacagatgaggggcgcacctattgacatttgaggggctgtccacaggcagaaaatccagcatttgcaagggtttccgcccgtttttcggccaccgctaacctgtcttttaacctgcttttaaaccaatatttataaaccttgtttttaaccagggctgcgccctgtgcgcgtgaccgcgcacgccgaaggggggtgcccccccttctcgaaccctcccggcccgctaacgcgggcctcccatccccccaggggctgcgcccctcggccgcgaacggcctcaccccaaaaatggcagccaagctgaccacttctgcgctcggcccttccggctggctggtttattgctgataaatctggagccggtgagcgtgggtctcgcggtatcattgcagcactggggccagatggtaagccctcccgtatcgtagttatctacacgacggggagtcaggcaactatggatgaacgaaatagacagatcgctgagataggtgcctcactgattaagcattggtaactgtcagaccaagtttactcatatatactttagattgatttaaaacttcatttttaatttaaaaggatctaggtgaagatcctttttgataatctcatgaccaaaatcccttaacgtgagttttcgttccactgagcgtcagaccccgtagaaaagatcaaaggatcttcttgagatcctttttttctgcgcgtaatctgctgcttgcaaacaaaaaaaccaccgctaccagcggtggtttgtttgccggatcaagagctaccaactctttttccgaaggtaactggcttcagcagagcgcagataccaaatactgtccttctagtgtagccgtagttaggccaccacttcaagaactctgtagcaccgcctacatacctcgctctgctaatcctgttaccagtggctgctgccagtggcgataagtcgtgtcttaccgggttggactcaagacgatagttaccggataaggcgcagcggtcgggctgaacggggggttcgtgcacacagcccagcttggagcgaacgacctacaccgaactgagatacctacagcgtgagctatgagaaagcgccacgcttcccgaagggagaaaggcggacaggtatccggtaagcggcagggtcggaacaggagagcgcacgagggagcttccagggggaaacgcctggtatctttatagtcctgtcgggtttcgccacctctgacttgagcgtcgatttttgtgatgctcgtcaggggggcggagcctatggaaaaacgccagcaacgcggcctttttacggttcctggccttttgctggccttttgctcacatgttctttcctgcgttatcccctgattctgtggataaccgtattaccgcctttgagtgagctgataccgctcgccgcagccgaacgaccgagcgcagcgagtcagtgagcgaggaagcggaagagcgcccaatacgcaaaccgcctctccccgcgcgttggccgattcattaatgcagctggcacgacaggtttcccgactggaaagcgggcagtgagcgcaacgcaattaatgtgagttagctcactcattaggcaccccaggctttacactttatgcttccggctcgtatgttgtgtggaattgtgagcggataacaatttcacacaggaaacagctatgaccatgattacgccaagctagcttcccgcttggtcgggccgcttcgcgagggcccgttgacgacaacggtgcgatgggtcccggccccggtcaagacgatgccaatacgttgcgacactacgccttggcacttttagaattgccttatcgtcctgataagaaatgtccgaccagctaaagacatcgcgtccaatcaaagcctagaaaatataggcgaagggacgctaataagtctttcataagaccgcgcaaatctaaaaatatccttagattcacgatgcggcacttcggatgacttccgagcgagcctggaacctcagaaaaacgtctgagagataccgcggatctcacacaggaaacagctatgaccatgattacgccaagcttGTGACTGGAACGACATCTTTGAGAATCGCACTCGTCGGCGTGATCTACCTGCTCGCGCACACGGCCGCGAATGCGATGAATCATCCGGCGCAGACCGGGACGGGCGGCGGCCCGGAGACGACCATCACGGCTCCGCATGTCGCGCCGACAGGAAGAGTCATCCCTCGCCCGCAGGTCTTGGGCTCGCCGACAAACGGCGAGGCTGCGCAGAAGCCTGAACCTCAGTTCAAGGACAGCTTCCTCATGAAGGACATTTGTGTCGGCTGCTGAggatccccgggtaccgagctcgaattcactggccgtcgttttacaacgtcgtgactgggaaaaccctggcgttacccaacttaatcgccttgcagcacatccccctttcgccagctggcgtaatagcgaagaggcccgcaccgatcgcccttcccaacagttgcgcagcctgaatggcgaatggcgcctgatgcggtattttctccttacgcatctgtgcggtatttcacaccgcatatggtgcactctcagtacaatctgctctgatgccgcatagttaagccagccccgacacccgccaacacccgctgacgcgccctgacgggcttgtctgctcccggcatccgcttacagacaagctgtgaccgtctccgggagctgcatgtgtcagaggttttcaccgtcatcaccgaaacgcgcgagacgaaagggcctcgtgatacgcctatttttataggttaatgtcatgataataatggtttcttagcaccctttctcggtccttcaacgttcctgacaacgagcctccttttcgccaatccatcgacaatcaccgcgagtccctgctcgaacgctgcgtccggaccggcttcgtcgaaggcgtctatcgcggcccgcaacagcggcgagagcggagcctgttcaacggtgccgccgcgctcgccggcatcgctgtcgccggcctgctcctcaagcacggccccaacagtgaagtagctgattgtcatcagcgcattgacggcgtccccggccgaaaaacccgcctcgcagaggaagcgaagctgcgcgtcggccgtttccatctgcggtgcgcccggtcgcgtgccggcatggatgcgcgcgccatcgcggtaggcgagcagcgcctgcctgaagctgcgggcattcccgatcagaaatgagcgccagtcgtcgtcggctctcggcaccgaatgcgtatgattctccgccagcatggcttcggccagtgcgtcgagcagcgcccgcttgttcctgaagtgccagtaaagcgccggctgctgaacccccaaccgttccgccagtttgcgtgtcgtcagaccgtctacgccgacctcgttcaacaggtccagggcggcacggatcactgtattcggctgcaactttgtcatgattgacactttatcactgataaacataatatgtccaccaacttatcagtgataaagaatccgcgcgttcaatcggaccagcggaggctggtccggaggccagacgtgaaacccaacatacccctgatcgtaattctgagcactgtcgcgctcgacgctgtcggcatcggcctgattatgccggtgctgccgggcctcctgcgcgatctggttcactcgaacgacgtcaccgcccactatggcattctgctggcgctgtatgcgttggtgcaatttgcctgcgcacctgtgctgggcgcgctgtcggatcgtttcgggcggcggccaatcttgctcgtctcgctggccggcgccactgtcgactacgccatcatggcgacagcgcctttcctttgggttctctatatcgggcggatcgtggccggcatcaccggggcgactggggcggtagccggcgcttatattgccgatatcactgatggcgatgagcgcgcgcggcacttcggcttcatgagcgcctgtttcgggttcgggatggtcgcgggacctgtgctcggtgggctgatgggcggtttctccccccacgctccgttcttcgccgcggcagccttgaacggcctcaatttcctgacgggctgtttccttttgccggagtcgcacaaaggcgaacgccggccgttacgccgggaggctctcaacccgctcgcttcgttccggtgggcccggggcatgaccgtcgtcgccgccctgatggcggtcttcttcatcatgcaacttgtcggacaggtgccggccgcgctttgggtcattttcggcgaggatcgctttcactgggacgcgaccacgatcggcatttcgcttgccgcatttggcattctgcattcactcgcccaggcaatgatcaccggccctgtagccgcccggctcggcgaaaggcgggcactcatgctcggaatgattgccgacggcacaggctacatcctgcttgccttcgcgacacggggatggatggcgttcccgatcatggtcctgcttgcttcgggtggcatcggaatgccggcgctgcaagcaatgttgtccaggcaggtggatgaggaacgtcaggggcagctgcaaggctcactggcggcgctcaccagcctgacctcgatcgtcggacccctcctcttcacggcgatctatgcggcttctataacaacgtggaacgggtgggcatggattgcaggcgctgccctctacttgctctgcctgccggcgctgcgtcgcgggctttggagcggcgcagggcaacgagccgatcgctgatcgtggaaacgataggcctatgccatgcgggtcaaggcgacttccggcaagctatacgcgccctagaattgtcaattttaatcctctgtttatcggcagttcgtagagcgcgccgtgcgtcccgagcgatactgagcgaagcaagtgcgtcgagcagtgcccgcttgttcctgaaatgccagtaaagcgctggctgctgaacccccagccggaactgaccccacaaggccctagcgtttgcaatgcaccaggtcatcattgacccaggcgtgttccaccaggccgctgcctcgcaactcttcgcaggcttcgccgacctgctcgcgccacttcttcacgcgggtggaatccgatccgcacatgaggcggaaggtttccagcttgagcgggtacggctcccggtgcgagctgaaatagtcgaacatccgtcgggccgtcggcgacagcttgcggtacttctcccatatgaatttcgtgtagtggtcgccagcaaacagcacgacgatttcctcgtcgatcaggacctggcaacgggacgttttcttgccacggtccaggacgcggaagcggtgcagcagcgacaccgattccaggtgcccaacgcggtcggacgtgaagcccatcgccgtcgcctgtaggcgcgacaggcattcctcggccttcgtgtaataccggccattgatcgaccagcccaggtcctggcaaagctcgtagaacgtgaaggtgatcggctcgccgataggggtgcgcttcgcgtactccaacacctgctgccacaccagttcgtcatcgtcggcccgcagctcgacgccggtgtaggtgatcttcacgtccttgttgacgtggaaaatgaccttgttttgcagcgcctcgcgcgggattttcttgttgcgcgtggtgaacagggcagagcgggccgtgtcgtttggcatcgctcgcatcgtgtccggccacggcgcaatatcgaacaaggaaagctgcatttccttgatctgctgcttcgtgtgtttcagcaacgcggcctgcttggcctcgctgacctgttttgccaggtcctcgccggcggtttttcgcttcttggtcgtcatagttcctcgcgtgtcgatggtcatcgacttcgccaaacctgccgcctcctgttcgagacgacgcgaacgctccacggcggccgatggcgcgggcagggcagggggagccagttgcacgctgtcgcgctcgatcttggccgtagcttgctggaccatcgagccgacggactggaaggtttcgcggggcgcacgcatgacggtgcggcttgcgatggtttcggcatcctcggcggaaaaccccgcgtcgatcagttcttgcctgtatgccttccggtcaaacgtccgattcattcaccctccttgcgggattgccccgactcacgccggggcaatgtgcccttattcctgatttgacccgcctggtgccttggtgtccagataatccaccttatcggcaatgaagtcggtcccgtagaccgtctggccgtccttctcgtacttggtattccgaatcttgccctgcacgaataccagctccgcgaagtcgctcttcttgatggagcgcatggggacgtgcttggcaatcacgcgcaccccccggccgttttagcggctaaaaaagtcatggctctgccctcgggcggaccacgcccatcatgaccttgccaagctcgtcctgcttctcttcgatcttcgccagcagggcgaggatcgtggcatcaccgaaccgcgccgtgcgcgggtcgtcggtgagccagagtttcagcaggccgcccaggcggcccaggtcgccattgatgcgggccagctcgcggacgtgctcatagtccacgacgcccgtgattttgtagccctggccgacggccagcaggtaggcctacaggctcatgccggccgccgccgccttttcctcaatcgctcttcgttcgtctggaaggcagtacaccttgataggtgggctgcccttcctggttggcttggtttcatcagccatccgcttgccctcatctgttacgccggcggtagccggccagcctcgcagagcaggattcccgttgagcaccgccaggtgcgaataagggacagtgaagaaggaacacccgctcgcgggtgggcctacttcacctatcctgcccggctgacgccgttggatacaccaaggaaagtctacacgaaccctttggcaaaatcctgtatatcgtgcgaaaaaggatggatataccgaaaaaatcgctataatgaccccgaagcagggttatgcagcggaaaagatccgtc-3´

**pCM80-3029, the sequences in red encode the gene META1_3029**

5´-

gaccctttccgacgctcaccgggctggttgccctcgccgctgggctggcggccgtctatggccctgcaaacgcgccagaaacgccgtcgaagccgtgtgcgagacaccgcggccgccggcgttgtggatacctcgcggaaaacttggccctcactgacagatgaggggcggacgttgacacttgaggggccgactcacccggcgcggcgttgacagatgaggggcaggctcgatttcggccggcgacgtggagctggccagcctcgcaaatcggcgaaaacgcctgattttacgcgagtttcccacagatgatgtggacaagcctggggataagtgccctgcggtattgacacttgaggggcgcgactactgacagatgaggggcgcgatccttgacacttgaggggcagagtgctgacagatgaggggcgcacctattgacatttgaggggctgtccacaggcagaaaatccagcatttgcaagggtttccgcccgtttttcggccaccgctaacctgtcttttaacctgcttttaaaccaatatttataaaccttgtttttaaccagggctgcgccctgtgcgcgtgaccgcgcacgccgaaggggggtgcccccccttctcgaaccctcccggcccgctaacgcgggcctcccatccccccaggggctgcgcccctcggccgcgaacggcctcaccccaaaaatggcagccaagctgaccacttctgcgctcggcccttccggctggctggtttattgctgataaatctggagccggtgagcgtgggtctcgcggtatcattgcagcactggggccagatggtaagccctcccgtatcgtagttatctacacgacggggagtcaggcaactatggatgaacgaaatagacagatcgctgagataggtgcctcactgattaagcattggtaactgtcagaccaagtttactcatatatactttagattgatttaaaacttcatttttaatttaaaaggatctaggtgaagatcctttttgataatctcatgaccaaaatcccttaacgtgagttttcgttccactgagcgtcagaccccgtagaaaagatcaaaggatcttcttgagatcctttttttctgcgcgtaatctgctgcttgcaaacaaaaaaaccaccgctaccagcggtggtttgtttgccggatcaagagctaccaactctttttccgaaggtaactggcttcagcagagcgcagataccaaatactgtccttctagtgtagccgtagttaggccaccacttcaagaactctgtagcaccgcctacatacctcgctctgctaatcctgttaccagtggctgctgccagtggcgataagtcgtgtcttaccgggttggactcaagacgatagttaccggataaggcgcagcggtcgggctgaacggggggttcgtgcacacagcccagcttggagcgaacgacctacaccgaactgagatacctacagcgtgagctatgagaaagcgccacgcttcccgaagggagaaaggcggacaggtatccggtaagcggcagggtcggaacaggagagcgcacgagggagcttccagggggaaacgcctggtatctttatagtcctgtcgggtttcgccacctctgacttgagcgtcgatttttgtgatgctcgtcaggggggcggagcctatggaaaaacgccagcaacgcggcctttttacggttcctggccttttgctggccttttgctcacatgttctttcctgcgttatcccctgattctgtggataaccgtattaccgcctttgagtgagctgataccgctcgccgcagccgaacgaccgagcgcagcgagtcagtgagcgaggaagcggaagagcgcccaatacgcaaaccgcctctccccgcgcgttggccgattcattaatgcagctggcacgacaggtttcccgactggaaagcgggcagtgagcgcaacgcaattaatgtgagttagctcactcattaggcaccccaggctttacactttatgcttccggctcgtatgttgtgtggaattgtgagcggataacaatttcacacaggaaacagctatgaccatgattacgccaagctagcttcccgcttggtcgggccgcttcgcgagggcccgttgacgacaacggtgcgatgggtcccggccccggtcaagacgatgccaatacgttgcgacactacgccttggcacttttagaattgccttatcgtcctgataagaaatgtccgaccagctaaagacatcgcgtccaatcaaagcctagaaaatataggcgaagggacgctaataagtctttcataagaccgcgcaaatctaaaaatatccttagattcacgatgcggcacttcggatgacttccgagcgagcctggaacctcagaaaaacgtctgagagataccgcggatctcacacaggaaacagctatgaccatgattacgccaagcttATGTTCGATATGAAGCGGATTGTGACACTCGGTCTCCTGGCGGCTGCGGCCGTTGCTTTGCCGCTCACGGCGCAGGCGCAGGACGGTGGAAACGGCAGCCGCCAGCAGGCCGGCGGCGGTTTCATGAGCAGCTACGTGGACGATCCCTACCACGACCCCCGTTCGCTCCACTCGCAGCGTGCGGGCTCGGGCCAGATCCTCGGCGCGCCGATGCTGAGCGAGAACGCCGGCGCCAAGGGTGGCGACATTCGCCCCGCCGCCCGTCTCGGCGCCGTGCAGACGCATTGGTCCGCCGGCACCGCGCCGCGCCGCGCGCGCTAAggatccccgggtaccgagctcgaattcactggccgtcgttttacaacgtcgtgactgggaaaaccctggcgttacccaacttaatcgccttgcagcacatccccctttcgccagctggcgtaatagcgaagaggcccgcaccgatcgcccttcccaacagttgcgcagcctgaatggcgaatggcgcctgatgcggtattttctccttacgcatctgtgcggtatttcacaccgcatatggtgcactctcagtacaatctgctctgatgccgcatagttaagccagccccgacacccgccaacacccgctgacgcgccctgacgggcttgtctgctcccggcatccgcttacagacaagctgtgaccgtctccgggagctgcatgtgtcagaggttttcaccgtcatcaccgaaacgcgcgagacgaaagggcctcgtgatacgcctatttttataggttaatgtcatgataataatggtttcttagcaccctttctcggtccttcaacgttcctgacaacgagcctccttttcgccaatccatcgacaatcaccgcgagtccctgctcgaacgctgcgtccggaccggcttcgtcgaaggcgtctatcgcggcccgcaacagcggcgagagcggagcctgttcaacggtgccgccgcgctcgccggcatcgctgtcgccggcctgctcctcaagcacggccccaacagtgaagtagctgattgtcatcagcgcattgacggcgtccccggccgaaaaacccgcctcgcagaggaagcgaagctgcgcgtcggccgtttccatctgcggtgcgcccggtcgcgtgccggcatggatgcgcgcgccatcgcggtaggcgagcagcgcctgcctgaagctgcgggcattcccgatcagaaatgagcgccagtcgtcgtcggctctcggcaccgaatgcgtatgattctccgccagcatggcttcggccagtgcgtcgagcagcgcccgcttgttcctgaagtgccagtaaagcgccggctgctgaacccccaaccgttccgccagtttgcgtgtcgtcagaccgtctacgccgacctcgttcaacaggtccagggcggcacggatcactgtattcggctgcaactttgtcatgattgacactttatcactgataaacataatatgtccaccaacttatcagtgataaagaatccgcgcgttcaatcggaccagcggaggctggtccggaggccagacgtgaaacccaacatacccctgatcgtaattctgagcactgtcgcgctcgacgctgtcggcatcggcctgattatgccggtgctgccgggcctcctgcgcgatctggttcactcgaacgacgtcaccgcccactatggcattctgctggcgctgtatgcgttggtgcaatttgcctgcgcacctgtgctgggcgcgctgtcggatcgtttcgggcggcggccaatcttgctcgtctcgctggccggcgccactgtcgactacgccatcatggcgacagcgcctttcctttgggttctctatatcgggcggatcgtggccggcatcaccggggcgactggggcggtagccggcgcttatattgccgatatcactgatggcgatgagcgcgcgcggcacttcggcttcatgagcgcctgtttcgggttcgggatggtcgcgggacctgtgctcggtgggctgatgggcggtttctccccccacgctccgttcttcgccgcggcagccttgaacggcctcaatttcctgacgggctgtttccttttgccggagtcgcacaaaggcgaacgccggccgttacgccgggaggctctcaacccgctcgcttcgttccggtgggcccggggcatgaccgtcgtcgccgccctgatggcggtcttcttcatcatgcaacttgtcggacaggtgccggccgcgctttgggtcattttcggcgaggatcgctttcactgggacgcgaccacgatcggcatttcgcttgccgcatttggcattctgcattcactcgcccaggcaatgatcaccggccctgtagccgcccggctcggcgaaaggcgggcactcatgctcggaatgattgccgacggcacaggctacatcctgcttgccttcgcgacacggggatggatggcgttcccgatcatggtcctgcttgcttcgggtggcatcggaatgccggcgctgcaagcaatgttgtccaggcaggtggatgaggaacgtcaggggcagctgcaaggctcactggcggcgctcaccagcctgacctcgatcgtcggacccctcctcttcacggcgatctatgcggcttctataacaacgtggaacgggtgggcatggattgcaggcgctgccctctacttgctctgcctgccggcgctgcgtcgcgggctttggagcggcgcagggcaacgagccgatcgctgatcgtggaaacgataggcctatgccatgcgggtcaaggcgacttccggcaagctatacgcgccctagaattgtcaattttaatcctctgtttatcggcagttcgtagagcgcgccgtgcgtcccgagcgatactgagcgaagcaagtgcgtcgagcagtgcccgcttgttcctgaaatgccagtaaagcgctggctgctgaacccccagccggaactgaccccacaaggccctagcgtttgcaatgcaccaggtcatcattgacccaggcgtgttccaccaggccgctgcctcgcaactcttcgcaggcttcgccgacctgctcgcgccacttcttcacgcgggtggaatccgatccgcacatgaggcggaaggtttccagcttgagcgggtacggctcccggtgcgagctgaaatagtcgaacatccgtcgggccgtcggcgacagcttgcggtacttctcccatatgaatttcgtgtagtggtcgccagcaaacagcacgacgatttcctcgtcgatcaggacctggcaacgggacgttttcttgccacggtccaggacgcggaagcggtgcagcagcgacaccgattccaggtgcccaacgcggtcggacgtgaagcccatcgccgtcgcctgtaggcgcgacaggcattcctcggccttcgtgtaataccggccattgatcgaccagcccaggtcctggcaaagctcgtagaacgtgaaggtgatcggctcgccgataggggtgcgcttcgcgtactccaacacctgctgccacaccagttcgtcatcgtcggcccgcagctcgacgccggtgtaggtgatcttcacgtccttgttgacgtggaaaatgaccttgttttgcagcgcctcgcgcgggattttcttgttgcgcgtggtgaacagggcagagcgggccgtgtcgtttggcatcgctcgcatcgtgtccggccacggcgcaatatcgaacaaggaaagctgcatttccttgatctgctgcttcgtgtgtttcagcaacgcggcctgcttggcctcgctgacctgttttgccaggtcctcgccggcggtttttcgcttcttggtcgtcatagttcctcgcgtgtcgatggtcatcgacttcgccaaacctgccgcctcctgttcgagacgacgcgaacgctccacggcggccgatggcgcgggcagggcagggggagccagttgcacgctgtcgcgctcgatcttggccgtagcttgctggaccatcgagccgacggactggaaggtttcgcggggcgcacgcatgacggtgcggcttgcgatggtttcggcatcctcggcggaaaaccccgcgtcgatcagttcttgcctgtatgccttccggtcaaacgtccgattcattcaccctccttgcgggattgccccgactcacgccggggcaatgtgcccttattcctgatttgacccgcctggtgccttggtgtccagataatccaccttatcggcaatgaagtcggtcccgtagaccgtctggccgtccttctcgtacttggtattccgaatcttgccctgcacgaataccagctccgcgaagtcgctcttcttgatggagcgcatggggacgtgcttggcaatcacgcgcaccccccggccgttttagcggctaaaaaagtcatggctctgccctcgggcggaccacgcccatcatgaccttgccaagctcgtcctgcttctcttcgatcttcgccagcagggcgaggatcgtggcatcaccgaaccgcgccgtgcgcgggtcgtcggtgagccagagtttcagcaggccgcccaggcggcccaggtcgccattgatgcgggccagctcgcggacgtgctcatagtccacgacgcccgtgattttgtagccctggccgacggccagcaggtaggcctacaggctcatgccggccgccgccgccttttcctcaatcgctcttcgttcgtctggaaggcagtacaccttgataggtgggctgcccttcctggttggcttggtttcatcagccatccgcttgccctcatctgttacgccggcggtagccggccagcctcgcagagcaggattcccgttgagcaccgccaggtgcgaataagggacagtgaagaaggaacacccgctcgcgggtgggcctacttcacctatcctgcccggctgacgccgttggatacaccaaggaaagtctacacgaaccctttggcaaaatcctgtatatcgtgcgaaaaaggatggatataccgaaaaaatcgctataatgaccccgaagcagggttatgcagcggaaaagatccgtc-3´

**pCM80-1261, the sequences in red encode the gene *META1_1261***

5´-

gaccctttccgacgctcaccgggctggttgccctcgccgctgggctggcggccgtctatggccctgcaaacgcgccagaaacgccgtcgaagccgtgtgcgagacaccgcggccgccggcgttgtggatacctcgcggaaaacttggccctcactgacagatgaggggcggacgttgacacttgaggggccgactcacccggcgcggcgttgacagatgaggggcaggctcgatttcggccggcgacgtggagctggccagcctcgcaaatcggcgaaaacgcctgattttacgcgagtttcccacagatgatgtggacaagcctggggataagtgccctgcggtattgacacttgaggggcgcgactactgacagatgaggggcgcgatccttgacacttgaggggcagagtgctgacagatgaggggcgcacctattgacatttgaggggctgtccacaggcagaaaatccagcatttgcaagggtttccgcccgtttttcggccaccgctaacctgtcttttaacctgcttttaaaccaatatttataaaccttgtttttaaccagggctgcgccctgtgcgcgtgaccgcgcacgccgaaggggggtgcccccccttctcgaaccctcccggcccgctaacgcgggcctcccatccccccaggggctgcgcccctcggccgcgaacggcctcaccccaaaaatggcagccaagctgaccacttctgcgctcggcccttccggctggctggtttattgctgataaatctggagccggtgagcgtgggtctcgcggtatcattgcagcactggggccagatggtaagccctcccgtatcgtagttatctacacgacggggagtcaggcaactatggatgaacgaaatagacagatcgctgagataggtgcctcactgattaagcattggtaactgtcagaccaagtttactcatatatactttagattgatttaaaacttcatttttaatttaaaaggatctaggtgaagatcctttttgataatctcatgaccaaaatcccttaacgtgagttttcgttccactgagcgtcagaccccgtagaaaagatcaaaggatcttcttgagatcctttttttctgcgcgtaatctgctgcttgcaaacaaaaaaaccaccgctaccagcggtggtttgtttgccggatcaagagctaccaactctttttccgaaggtaactggcttcagcagagcgcagataccaaatactgtccttctagtgtagccgtagttaggccaccacttcaagaactctgtagcaccgcctacatacctcgctctgctaatcctgttaccagtggctgctgccagtggcgataagtcgtgtcttaccgggttggactcaagacgatagttaccggataaggcgcagcggtcgggctgaacggggggttcgtgcacacagcccagcttggagcgaacgacctacaccgaactgagatacctacagcgtgagctatgagaaagcgccacgcttcccgaagggagaaaggcggacaggtatccggtaagcggcagggtcggaacaggagagcgcacgagggagcttccagggggaaacgcctggtatctttatagtcctgtcgggtttcgccacctctgacttgagcgtcgatttttgtgatgctcgtcaggggggcggagcctatggaaaaacgccagcaacgcggcctttttacggttcctggccttttgctggccttttgctcacatgttctttcctgcgttatcccctgattctgtggataaccgtattaccgcctttgagtgagctgataccgctcgccgcagccgaacgaccgagcgcagcgagtcagtgagcgaggaagcggaagagcgcccaatacgcaaaccgcctctccccgcgcgttggccgattcattaatgcagctggcacgacaggtttcccgactggaaagcgggcagtgagcgcaacgcaattaatgtgagttagctcactcattaggcaccccaggctttacactttatgcttccggctcgtatgttgtgtggaattgtgagcggataacaatttcacacaggaaacagctatgaccatgattacgccaagctagcttcccgcttggtcgggccgcttcgcgagggcccgttgacgacaacggtgcgatgggtcccggccccggtcaagacgatgccaatacgttgcgacactacgccttggcacttttagaattgccttatcgtcctgataagaaatgtccgaccagctaaagacatcgcgtccaatcaaagcctagaaaatataggcgaagggacgctaataagtctttcataagaccgcgcaaatctaaaaatatccttagattcacgatgcggcacttcggatgacttccgagcgagcctggaacctcagaaaaacgtctgagagataccgcggatctcacacaggaaacagctatgaccatgattacgccaagcttATGCGCTCGAAACGCTCCGAAATCGTCGACCTGCTGGTGCCCTTGCGGCGCTACGCCCGGTCGCTCACGCGGGATTCGCTGAAAGCGGATGACCTCGTGCACGATACCATGGTTCGCGCCCTCGAATCGCGCGCGAACCTGAGGCCGGACACCAACCTGCGCACCTGGATGATGACGGTGCTGCACAACGTCTTCATCGATGAGCAGCGCCGCAAACGCGTCGAGGCACGCCACGCCGATGTGCTGGTGCAGCTCTCGGACGACGTGGCACCGCCGGCCCAAGAGGGGCAGGTGCGGCTCATGCAGATCCGCAAGGCGTTCGAAGGACTGCCCGAGGAGCAGCGCGCCGCGCTCCACCTCGTGACGCTCGAAGGCATGGCTTATGCCGACGCGGCGGCCGTGCTCGGTATCCCGATCGGAACCCTGATGTCGCGGCTCGGCCGCGGCCGTGCGGCCCTACGCGCCTTCGAGGAAGGCGGCCGCAAGGGCAAGGCCACCGCCGGATCCTCCGACGATGGCCCCGACCGTGGCCGTGCACATCTGCGCGTCGTCGGTGGGAATTGAggatccccgggtaccgagctcgaattcactggccgtcgttttacaacgtcgtgactgggaaaaccctggcgttacccaacttaatcgccttgcagcacatccccctttcgccagctggcgtaatagcgaagaggcccgcaccgatcgcccttcccaacagttgcgcagcctgaatggcgaatggcgcctgatgcggtattttctccttacgcatctgtgcggtatttcacaccgcatatggtgcactctcagtacaatctgctctgatgccgcatagttaagccagccccgacacccgccaacacccgctgacgcgccctgacgggcttgtctgctcccggcatccgcttacagacaagctgtgaccgtctccgggagctgcatgtgtcagaggttttcaccgtcatcaccgaaacgcgcgagacgaaagggcctcgtgatacgcctatttttataggttaatgtcatgataataatggtttcttagcaccctttctcggtccttcaacgttcctgacaacgagcctccttttcgccaatccatcgacaatcaccgcgagtccctgctcgaacgctgcgtccggaccggcttcgtcgaaggcgtctatcgcggcccgcaacagcggcgagagcggagcctgttcaacggtgccgccgcgctcgccggcatcgctgtcgccggcctgctcctcaagcacggccccaacagtgaagtagctgattgtcatcagcgcattgacggcgtccccggccgaaaaacccgcctcgcagaggaagcgaagctgcgcgtcggccgtttccatctgcggtgcgcccggtcgcgtgccggcatggatgcgcgcgccatcgcggtaggcgagcagcgcctgcctgaagctgcgggcattcccgatcagaaatgagcgccagtcgtcgtcggctctcggcaccgaatgcgtatgattctccgccagcatggcttcggccagtgcgtcgagcagcgcccgcttgttcctgaagtgccagtaaagcgccggctgctgaacccccaaccgttccgccagtttgcgtgtcgtcagaccgtctacgccgacctcgttcaacaggtccagggcggcacggatcactgtattcggctgcaactttgtcatgattgacactttatcactgataaacataatatgtccaccaacttatcagtgataaagaatccgcgcgttcaatcggaccagcggaggctggtccggaggccagacgtgaaacccaacatacccctgatcgtaattctgagcactgtcgcgctcgacgctgtcggcatcggcctgattatgccggtgctgccgggcctcctgcgcgatctggttcactcgaacgacgtcaccgcccactatggcattctgctggcgctgtatgcgttggtgcaatttgcctgcgcacctgtgctgggcgcgctgtcggatcgtttcgggcggcggccaatcttgctcgtctcgctggccggcgccactgtcgactacgccatcatggcgacagcgcctttcctttgggttctctatatcgggcggatcgtggccggcatcaccggggcgactggggcggtagccggcgcttatattgccgatatcactgatggcgatgagcgcgcgcggcacttcggcttcatgagcgcctgtttcgggttcgggatggtcgcgggacctgtgctcggtgggctgatgggcggtttctccccccacgctccgttcttcgccgcggcagccttgaacggcctcaatttcctgacgggctgtttccttttgccggagtcgcacaaaggcgaacgccggccgttacgccgggaggctctcaacccgctcgcttcgttccggtgggcccggggcatgaccgtcgtcgccgccctgatggcggtcttcttcatcatgcaacttgtcggacaggtgccggccgcgctttgggtcattttcggcgaggatcgctttcactgggacgcgaccacgatcggcatttcgcttgccgcatttggcattctgcattcactcgcccaggcaatgatcaccggccctgtagccgcccggctcggcgaaaggcgggcactcatgctcggaatgattgccgacggcacaggctacatcctgcttgccttcgcgacacggggatggatggcgttcccgatcatggtcctgcttgcttcgggtggcatcggaatgccggcgctgcaagcaatgttgtccaggcaggtggatgaggaacgtcaggggcagctgcaaggctcactggcggcgctcaccagcctgacctcgatcgtcggacccctcctcttcacggcgatctatgcggcttctataacaacgtggaacgggtgggcatggattgcaggcgctgccctctacttgctctgcctgccggcgctgcgtcgcgggctttggagcggcgcagggcaacgagccgatcgctgatcgtggaaacgataggcctatgccatgcgggtcaaggcgacttccggcaagctatacgcgccctagaattgtcaattttaatcctctgtttatcggcagttcgtagagcgcgccgtgcgtcccgagcgatactgagcgaagcaagtgcgtcgagcagtgcccgcttgttcctgaaatgccagtaaagcgctggctgctgaacccccagccggaactgaccccacaaggccctagcgtttgcaatgcaccaggtcatcattgacccaggcgtgttccaccaggccgctgcctcgcaactcttcgcaggcttcgccgacctgctcgcgccacttcttcacgcgggtggaatccgatccgcacatgaggcggaaggtttccagcttgagcgggtacggctcccggtgcgagctgaaatagtcgaacatccgtcgggccgtcggcgacagcttgcggtacttctcccatatgaatttcgtgtagtggtcgccagcaaacagcacgacgatttcctcgtcgatcaggacctggcaacgggacgttttcttgccacggtccaggacgcggaagcggtgcagcagcgacaccgattccaggtgcccaacgcggtcggacgtgaagcccatcgccgtcgcctgtaggcgcgacaggcattcctcggccttcgtgtaataccggccattgatcgaccagcccaggtcctggcaaagctcgtagaacgtgaaggtgatcggctcgccgataggggtgcgcttcgcgtactccaacacctgctgccacaccagttcgtcatcgtcggcccgcagctcgacgccggtgtaggtgatcttcacgtccttgttgacgtggaaaatgaccttgttttgcagcgcctcgcgcgggattttcttgttgcgcgtggtgaacagggcagagcgggccgtgtcgtttggcatcgctcgcatcgtgtccggccacggcgcaatatcgaacaaggaaagctgcatttccttgatctgctgcttcgtgtgtttcagcaacgcggcctgcttggcctcgctgacctgttttgccaggtcctcgccggcggtttttcgcttcttggtcgtcatagttcctcgcgtgtcgatggtcatcgacttcgccaaacctgccgcctcctgttcgagacgacgcgaacgctccacggcggccgatggcgcgggcagggcagggggagccagttgcacgctgtcgcgctcgatcttggccgtagcttgctggaccatcgagccgacggactggaaggtttcgcggggcgcacgcatgacggtgcggcttgcgatggtttcggcatcctcggcggaaaaccccgcgtcgatcagttcttgcctgtatgccttccggtcaaacgtccgattcattcaccctccttgcgggattgccccgactcacgccggggcaatgtgcccttattcctgatttgacccgcctggtgccttggtgtccagataatccaccttatcggcaatgaagtcggtcccgtagaccgtctggccgtccttctcgtacttggtattccgaatcttgccctgcacgaataccagctccgcgaagtcgctcttcttgatggagcgcatggggacgtgcttggcaatcacgcgcaccccccggccgttttagcggctaaaaaagtcatggctctgccctcgggcggaccacgcccatcatgaccttgccaagctcgtcctgcttctcttcgatcttcgccagcagggcgaggatcgtggcatcaccgaaccgcgccgtgcgcgggtcgtcggtgagccagagtttcagcaggccgcccaggcggcccaggtcgccattgatgcgggccagctcgcggacgtgctcatagtccacgacgcccgtgattttgtagccctggccgacggccagcaggtaggcctacaggctcatgccggccgccgccgccttttcctcaatcgctcttcgttcgtctggaaggcagtacaccttgataggtgggctgcccttcctggttggcttggtttcatcagccatccgcttgccctcatctgttacgccggcggtagccggccagcctcgcagagcaggattcccgttgagcaccgccaggtgcgaataagggacagtgaagaaggaacacccgctcgcgggtgggcctacttcacctatcctgcccggctgacgccgttggatacaccaaggaaagtctacacgaaccctttggcaaaatcctgtatatcgtgcgaaaaaggatggatataccgaaaaaatcgctataatgaccccgaagcagggttatgcagcggaaaagatccgtc-3´

**pCM80-1418, the sequences in red encodes the gene META1_1418**

5´-

gaccctttccgacgctcaccgggctggttgccctcgccgctgggctggcggccgtctatggccctgcaaacgcgccagaaacgccgtcgaagccgtgtgcgagacaccgcggccgccggcgttgtggatacctcgcggaaaacttggccctcactgacagatgaggggcggacgttgacacttgaggggccgactcacccggcgcggcgttgacagatgaggggcaggctcgatttcggccggcgacgtggagctggccagcctcgcaaatcggcgaaaacgcctgattttacgcgagtttcccacagatgatgtggacaagcctggggataagtgccctgcggtattgacacttgaggggcgcgactactgacagatgaggggcgcgatccttgacacttgaggggcagagtgctgacagatgaggggcgcacctattgacatttgaggggctgtccacaggcagaaaatccagcatttgcaagggtttccgcccgtttttcggccaccgctaacctgtcttttaacctgcttttaaaccaatatttataaaccttgtttttaaccagggctgcgccctgtgcgcgtgaccgcgcacgccgaaggggggtgcccccccttctcgaaccctcccggcccgctaacgcgggcctcccatccccccaggggctgcgcccctcggccgcgaacggcctcaccccaaaaatggcagccaagctgaccacttctgcgctcggcccttccggctggctggtttattgctgataaatctggagccggtgagcgtgggtctcgcggtatcattgcagcactggggccagatggtaagccctcccgtatcgtagttatctacacgacggggagtcaggcaactatggatgaacgaaatagacagatcgctgagataggtgcctcactgattaagcattggtaactgtcagaccaagtttactcatatatactttagattgatttaaaacttcatttttaatttaaaaggatctaggtgaagatcctttttgataatctcatgaccaaaatcccttaacgtgagttttcgttccactgagcgtcagaccccgtagaaaagatcaaaggatcttcttgagatcctttttttctgcgcgtaatctgctgcttgcaaacaaaaaaaccaccgctaccagcggtggtttgtttgccggatcaagagctaccaactctttttccgaaggtaactggcttcagcagagcgcagataccaaatactgtccttctagtgtagccgtagttaggccaccacttcaagaactctgtagcaccgcctacatacctcgctctgctaatcctgttaccagtggctgctgccagtggcgataagtcgtgtcttaccgggttggactcaagacgatagttaccggataaggcgcagcggtcgggctgaacggggggttcgtgcacacagcccagcttggagcgaacgacctacaccgaactgagatacctacagcgtgagctatgagaaagcgccacgcttcccgaagggagaaaggcggacaggtatccggtaagcggcagggtcggaacaggagagcgcacgagggagcttccagggggaaacgcctggtatctttatagtcctgtcgggtttcgccacctctgacttgagcgtcgatttttgtgatgctcgtcaggggggcggagcctatggaaaaacgccagcaacgcggcctttttacggttcctggccttttgctggccttttgctcacatgttctttcctgcgttatcccctgattctgtggataaccgtattaccgcctttgagtgagctgataccgctcgccgcagccgaacgaccgagcgcagcgagtcagtgagcgaggaagcggaagagcgcccaatacgcaaaccgcctctccccgcgcgttggccgattcattaatgcagctggcacgacaggtttcccgactggaaagcgggcagtgagcgcaacgcaattaatgtgagttagctcactcattaggcaccccaggctttacactttatgcttccggctcgtatgttgtgtggaattgtgagcggataacaatttcacacaggaaacagctatgaccatgattacgccaagctagcttcccgcttggtcgggccgcttcgcgagggcccgttgacgacaacggtgcgatgggtcccggccccggtcaagacgatgccaatacgttgcgacactacgccttggcacttttagaattgccttatcgtcctgataagaaatgtccgaccagctaaagacatcgcgtccaatcaaagcctagaaaatataggcgaagggacgctaataagtctttcataagaccgcgcaaatctaaaaatatccttagattcacgatgcggcacttcggatgacttccgagcgagcctggaacctcagaaaaacgtctgagagataccgcggatctcacacaggaaacagctatgaccatgattacgccaagcttATGTTTCCTCAGGTTCTGACCGACGGTTACCGCAGCTTCCTCGGCGAGCGGCTGCCGAACGAGCGGCGCAAATACGAGATGCTCGGCAAGGAAGGCCAGGAGCCTGAAGTCCTGCTGATCGGCTGCTGCGACAGCCGCGTCGCCCCCGAGGTGATCTTCAATACGGGCCCGGGCCAGATATTCACCATCCGCAACGTGGCCAACATCGTGCCGCCCTCCCAGCCCGACGGCGCCTATCACGGCACCTCTTCGGCGATCGAGTTCGCGGTGCAGGCCTTGAAGGTGAAGCACATCGTCGTGCTCGGCCACGCCACCTGCGGCGGCATCAAGGCGGCCGGTTTCGGTGCCGAGCCGCTCTCCTCGGGCAACTTCATCGGCAGCTGGGTCTCGCTGGTCGAGCCGGCGACGAAGAAGCTCGCCGAGGCCGGCGATAGCAAGGACAAGGAGGGCTACCTCACCCGCCTCGAATACACGATGATCGGGCAGAGCCTCGAAAACCTGATGACCTTCGACTTCATCCGCGAGGCGGTCGAGGCGGGCAAGCTGCACCTGCACGGCGCGCATTTCGGCATCGAGACGGGTGAGCTGCGCATCCGCAACCCGAACACCGGCGAGTTCGAATCCGTGGTCTCCCGCGACGGCCAGTCGCTCGCGGCGTCCGCCCTGATCGGCTGCACCGCGGCTTAAggatccccgggtaccgagctcgaattcactggccgtcgttttacaacgtcgtgactgggaaaaccctggcgttacccaacttaatcgccttgcagcacatccccctttcgccagctggcgtaatagcgaagaggcccgcaccgatcgcccttcccaacagttgcgcagcctgaatggcgaatggcgcctgatgcggtattttctccttacgcatctgtgcggtatttcacaccgcatatggtgcactctcagtacaatctgctctgatgccgcatagttaagccagccccgacacccgccaacacccgctgacgcgccctgacgggcttgtctgctcccggcatccgcttacagacaagctgtgaccgtctccgggagctgcatgtgtcagaggttttcaccgtcatcaccgaaacgcgcgagacgaaagggcctcgtgatacgcctatttttataggttaatgtcatgataataatggtttcttagcaccctttctcggtccttcaacgttcctgacaacgagcctccttttcgccaatccatcgacaatcaccgcgagtccctgctcgaacgctgcgtccggaccggcttcgtcgaaggcgtctatcgcggcccgcaacagcggcgagagcggagcctgttcaacggtgccgccgcgctcgccggcatcgctgtcgccggcctgctcctcaagcacggccccaacagtgaagtagctgattgtcatcagcgcattgacggcgtccccggccgaaaaacccgcctcgcagaggaagcgaagctgcgcgtcggccgtttccatctgcggtgcgcccggtcgcgtgccggcatggatgcgcgcgccatcgcggtaggcgagcagcgcctgcctgaagctgcgggcattcccgatcagaaatgagcgccagtcgtcgtcggctctcggcaccgaatgcgtatgattctccgccagcatggcttcggccagtgcgtcgagcagcgcccgcttgttcctgaagtgccagtaaagcgccggctgctgaacccccaaccgttccgccagtttgcgtgtcgtcagaccgtctacgccgacctcgttcaacaggtccagggcggcacggatcactgtattcggctgcaactttgtcatgattgacactttatcactgataaacataatatgtccaccaacttatcagtgataaagaatccgcgcgttcaatcggaccagcggaggctggtccggaggccagacgtgaaacccaacatacccctgatcgtaattctgagcactgtcgcgctcgacgctgtcggcatcggcctgattatgccggtgctgccgggcctcctgcgcgatctggttcactcgaacgacgtcaccgcccactatggcattctgctggcgctgtatgcgttggtgcaatttgcctgcgcacctgtgctgggcgcgctgtcggatcgtttcgggcggcggccaatcttgctcgtctcgctggccggcgccactgtcgactacgccatcatggcgacagcgcctttcctttgggttctctatatcgggcggatcgtggccggcatcaccggggcgactggggcggtagccggcgcttatattgccgatatcactgatggcgatgagcgcgcgcggcacttcggcttcatgagcgcctgtttcgggttcgggatggtcgcgggacctgtgctcggtgggctgatgggcggtttctccccccacgctccgttcttcgccgcggcagccttgaacggcctcaatttcctgacgggctgtttccttttgccggagtcgcacaaaggcgaacgccggccgttacgccgggaggctctcaacccgctcgcttcgttccggtgggcccggggcatgaccgtcgtcgccgccctgatggcggtcttcttcatcatgcaacttgtcggacaggtgccggccgcgctttgggtcattttcggcgaggatcgctttcactgggacgcgaccacgatcggcatttcgcttgccgcatttggcattctgcattcactcgcccaggcaatgatcaccggccctgtagccgcccggctcggcgaaaggcgggcactcatgctcggaatgattgccgacggcacaggctacatcctgcttgccttcgcgacacggggatggatggcgttcccgatcatggtcctgcttgcttcgggtggcatcggaatgccggcgctgcaagcaatgttgtccaggcaggtggatgaggaacgtcaggggcagctgcaaggctcactggcggcgctcaccagcctgacctcgatcgtcggacccctcctcttcacggcgatctatgcggcttctataacaacgtggaacgggtgggcatggattgcaggcgctgccctctacttgctctgcctgccggcgctgcgtcgcgggctttggagcggcgcagggcaacgagccgatcgctgatcgtggaaacgataggcctatgccatgcgggtcaaggcgacttccggcaagctatacgcgccctagaattgtcaattttaatcctctgtttatcggcagttcgtagagcgcgccgtgcgtcccgagcgatactgagcgaagcaagtgcgtcgagcagtgcccgcttgttcctgaaatgccagtaaagcgctggctgctgaacccccagccggaactgaccccacaaggccctagcgtttgcaatgcaccaggtcatcattgacccaggcgtgttccaccaggccgctgcctcgcaactcttcgcaggcttcgccgacctgctcgcgccacttcttcacgcgggtggaatccgatccgcacatgaggcggaaggtttccagcttgagcgggtacggctcccggtgcgagctgaaatagtcgaacatccgtcgggccgtcggcgacagcttgcggtacttctcccatatgaatttcgtgtagtggtcgccagcaaacagcacgacgatttcctcgtcgatcaggacctggcaacgggacgttttcttgccacggtccaggacgcggaagcggtgcagcagcgacaccgattccaggtgcccaacgcggtcggacgtgaagcccatcgccgtcgcctgtaggcgcgacaggcattcctcggccttcgtgtaataccggccattgatcgaccagcccaggtcctggcaaagctcgtagaacgtgaaggtgatcggctcgccgataggggtgcgcttcgcgtactccaacacctgctgccacaccagttcgtcatcgtcggcccgcagctcgacgccggtgtaggtgatcttcacgtccttgttgacgtggaaaatgaccttgttttgcagcgcctcgcgcgggattttcttgttgcgcgtggtgaacagggcagagcgggccgtgtcgtttggcatcgctcgcatcgtgtccggccacggcgcaatatcgaacaaggaaagctgcatttccttgatctgctgcttcgtgtgtttcagcaacgcggcctgcttggcctcgctgacctgttttgccaggtcctcgccggcggtttttcgcttcttggtcgtcatagttcctcgcgtgtcgatggtcatcgacttcgccaaacctgccgcctcctgttcgagacgacgcgaacgctccacggcggccgatggcgcgggcagggcagggggagccagttgcacgctgtcgcgctcgatcttggccgtagcttgctggaccatcgagccgacggactggaaggtttcgcggggcgcacgcatgacggtgcggcttgcgatggtttcggcatcctcggcggaaaaccccgcgtcgatcagttcttgcctgtatgccttccggtcaaacgtccgattcattcaccctccttgcgggattgccccgactcacgccggggcaatgtgcccttattcctgatttgacccgcctggtgccttggtgtccagataatccaccttatcggcaatgaagtcggtcccgtagaccgtctggccgtccttctcgtacttggtattccgaatcttgccctgcacgaataccagctccgcgaagtcgctcttcttgatggagcgcatggggacgtgcttggcaatcacgcgcaccccccggccgttttagcggctaaaaaagtcatggctctgccctcgggcggaccacgcccatcatgaccttgccaagctcgtcctgcttctcttcgatcttcgccagcagggcgaggatcgtggcatcaccgaaccgcgccgtgcgcgggtcgtcggtgagccagagtttcagcaggccgcccaggcggcccaggtcgccattgatgcgggccagctcgcggacgtgctcatagtccacgacgcccgtgattttgtagccctggccgacggccagcaggtaggcctacaggctcatgccggccgccgccgccttttcctcaatcgctcttcgttcgtctggaaggcagtacaccttgataggtgggctgcccttcctggttggcttggtttcatcagccatccgcttgccctcatctgttacgccggcggtagccggccagcctcgcagagcaggattcccgttgagcaccgccaggtgcgaataagggacagtgaagaaggaacacccgctcgcgggtgggcctacttcacctatcctgcccggctgacgccgttggatacaccaaggaaagtctacacgaaccctttggcaaaatcctgtatatcgtgcgaaaaaggatggatataccgaaaaaatcgctataatgaccccgaagcagggttatgcagcggaaaagatccgtc-3´

**pCM80-3027 Plasmid overexpressing the gene META1_3027**

5´-

gaccctttccgacgctcaccgggctggttgccctcgccgctgggctggcggccgtctatggccctgcaaacgcgccagaaacgccgtcgaagccgtgtgcgagacaccgcggccgccggcgttgtggatacctcgcggaaaacttggccctcactgacagatgaggggcggacgttgacacttgaggggccgactcacccggcgcggcgttgacagatgaggggcaggctcgatttcggccggcgacgtggagctggccagcctcgcaaatcggcgaaaacgcctgattttacgcgagtttcccacagatgatgtggacaagcctggggataagtgccctgcggtattgacacttgaggggcgcgactactgacagatgaggggcgcgatccttgacacttgaggggcagagtgctgacagatgaggggcgcacctattgacatttgaggggctgtccacaggcagaaaatccagcatttgcaagggtttccgcccgtttttcggccaccgctaacctgtcttttaacctgcttttaaaccaatatttataaaccttgtttttaaccagggctgcgccctgtgcgcgtgaccgcgcacgccgaaggggggtgcccccccttctcgaaccctcccggcccgctaacgcgggcctcccatccccccaggggctgcgcccctcggccgcgaacggcctcaccccaaaaatggcagccaagctgaccacttctgcgctcggcccttccggctggctggtttattgctgataaatctggagccggtgagcgtgggtctcgcggtatcattgcagcactggggccagatggtaagccctcccgtatcgtagttatctacacgacggggagtcaggcaactatggatgaacgaaatagacagatcgctgagataggtgcctcactgattaagcattggtaactgtcagaccaagtttactcatatatactttagattgatttaaaacttcatttttaatttaaaaggatctaggtgaagatcctttttgataatctcatgaccaaaatcccttaacgtgagttttcgttccactgagcgtcagaccccgtagaaaagatcaaaggatcttcttgagatcctttttttctgcgcgtaatctgctgcttgcaaacaaaaaaaccaccgctaccagcggtggtttgtttgccggatcaagagctaccaactctttttccgaaggtaactggcttcagcagagcgcagataccaaatactgtccttctagtgtagccgtagttaggccaccacttcaagaactctgtagcaccgcctacatacctcgctctgctaatcctgttaccagtggctgctgccagtggcgataagtcgtgtcttaccgggttggactcaagacgatagttaccggataaggcgcagcggtcgggctgaacggggggttcgtgcacacagcccagcttggagcgaacgacctacaccgaactgagatacctacagcgtgagctatgagaaagcgccacgcttcccgaagggagaaaggcggacaggtatccggtaagcggcagggtcggaacaggagagcgcacgagggagcttccagggggaaacgcctggtatctttatagtcctgtcgggtttcgccacctctgacttgagcgtcgatttttgtgatgctcgtcaggggggcggagcctatggaaaaacgccagcaacgcggcctttttacggttcctggccttttgctggccttttgctcacatgttctttcctgcgttatcccctgattctgtggataaccgtattaccgcctttgagtgagctgataccgctcgccgcagccgaacgaccgagcgcagcgagtcagtgagcgaggaagcggaagagcgcccaatacgcaaaccgcctctccccgcgcgttggccgattcattaatgcagctggcacgacaggtttcccgactggaaagcgggcagtgagcgcaacgcaattaatgtgagttagctcactcattaggcaccccaggctttacactttatgcttccggctcgtatgttgtgtggaattgtgagcggataacaatttcacacaggaaacagctatgaccatgattacgccaagctagcttcccgcttggtcgggccgcttcgcgagggcccgttgacgacaacggtgcgatgggtcccggccccggtcaagacgatgccaatacgttgcgacactacgccttggcacttttagaattgccttatcgtcctgataagaaatgtccgaccagctaaagacatcgcgtccaatcaaagcctagaaaatataggcgaagggacgctaataagtctttcataagaccgcgcaaatctaaaaatatccttagattcacgatgcggcacttcggatgacttccgagcgagcctggaacctcagaaaaacgtctgagagataccgcggatctcacacaggaaacagctatgaccatgattacgccaagcttATGCGCGCCTCCCTCGCCCGGCTGATGCCATCGACCATCGGCCGGGACCTTCCGGCCTCCTTCGTCGTCTTCCTCGTGGCGATGCCTCTCTGCATGGGCATCGCTATGGCCTCCGGTGTGCCCGCCGAGCGCGGGCTCATCACCGGCATCATCGGCGGTATCGTCGTCGGCTTCCTCGCCGGCTCGCCGCTTCAGGTCAGCGGCCCGGCCGCGGGCCTTGCCGTCATCGTCTTCGAGTTCGTGCGTGAGCACGGCATCGACGCGCTCGGGCCGGTCCTCGTCGCGGCGGGCGCCATCCAGCTTCTCGCCGGGGCGCTCCGGGTCGGCGGCTGGTTCAGGGCGATCTCGCCGGCGGTCGTGCACGGCATGCTCGCCGGCATCGGCATCCTGATCGTGCTGGCCCAGATCCACGTGCTGACCGACGCCCTGCCCAAGGCCAGCGGCCTCGACAACCTCGTCGCCATCCCCGCCGCGTTCTTCAACTTCGTCTCCGGTCCGGACGGCAACCGCCCCGGGGCCGTGATCGTCGGCCTTGCCACCATCGTTGCGATGATCGGCTGGGAGAAGATCCGGCCGGCTAAGCTCAAGCTGATTCCCGGTGCGCTCATGGGCGTGCTGGCCGGCACCGTCGTGGCCGTCGTCGGCAGCATGGACGTCAAGCGCGTCGAGGTGCCGGAGAACATCTTCTCCGCCGTGACCATGCCGGGAATGGGCGACTGGAGCCGCTTGGCCGAGCCGGCGATGATCCTGATGGCGGTCACCCTCGCGGTCATCGCCAGCGCGGAGAGCCTGCTCTCGGCAGCGGCGGTGGACCGGATGCATGACGGCCCGCGCACGCAGTACAACCGCGAACTCGGGGCGCAGGGCATCGGCAACATCCTCTGCGGCCTCGCGGGCGGCCTGCCGATGACCGGTGTGATTGTCCGGTCCTCGGCCAACGTCCAGGCGGGTGCGGCGACGCGCGCCTCCACCATCCTGCACGGGAGCTGGATCCTCGCCTTCCTGCTGGTCCTGCCGATGGTGCTGAAGGTCGTGCCGACCGCCTCCCTCGCCGGTATCCTCGTGGTGACGGGCTGGCGCCTCGTGAGCCCCGCCCACGCCTTCCACCTGCACGAGCGCTACGGCCTGCCCACCGCCGCGATCTGGCTCGCGACCATGGTGATGGTCGTCGCGACCGACCTGTTGACCGGTGTTCTCACGGGCCTTGCGCTCAGCCTCCTCCAGGTGATCCCGCACTACCTGCGTGGTCCGCTCAAGATCGAGGGCGGCGCGTCGCAGACGGTGCAAGGCGGAGCGCTTCAGGCGGTGCCGGAACTGCGGCTCTCGGGTTCGGCGACCTTCCTCCAACTGCCGCATCTCAATGCGGCGCTGGAGCGGACGCCGGAAGGCTCGCCGATCCGTCTCGCAGCACAGGATCTACGCCACGTCGATCACACCTGCCTGGAGATGATGCGGGAATGGGCGACGCGGCGGGCCAAGACCGGGTCGAGGGTCGAGGTCGTCGGCGGCGGCGCGAGCGGCCTGCATCACAGCCTCGCGATGGTCGCTCACGCCGCGCCGAAGGACTGAggatccccgggtaccgagctcgaattcactggccgtcgttttacaacgtcgtgactgggaaaaccctggcgttacccaacttaatcgccttgcagcacatccccctttcgccagctggcgtaatagcgaagaggcccgcaccgatcgcccttcccaacagttgcgcagcctgaatggcgaatggcgcctgatgcggtattttctccttacgcatctgtgcggtatttcacaccgcatatggtgcactctcagtacaatctgctctgatgccgcatagttaagccagccccgacacccgccaacacccgctgacgcgccctgacgggcttgtctgctcccggcatccgcttacagacaagctgtgaccgtctccgggagctgcatgtgtcagaggttttcaccgtcatcaccgaaacgcgcgagacgaaagggcctcgtgatacgcctatttttataggttaatgtcatgataataatggtttcttagcaccctttctcggtccttcaacgttcctgacaacgagcctccttttcgccaatccatcgacaatcaccgcgagtccctgctcgaacgctgcgtccggaccggcttcgtcgaaggcgtctatcgcggcccgcaacagcggcgagagcggagcctgttcaacggtgccgccgcgctcgccggcatcgctgtcgccggcctgctcctcaagcacggccccaacagtgaagtagctgattgtcatcagcgcattgacggcgtccccggccgaaaaacccgcctcgcagaggaagcgaagctgcgcgtcggccgtttccatctgcggtgcgcccggtcgcgtgccggcatggatgcgcgcgccatcgcggtaggcgagcagcgcctgcctgaagctgcgggcattcccgatcagaaatgagcgccagtcgtcgtcggctctcggcaccgaatgcgtatgattctccgccagcatggcttcggccagtgcgtcgagcagcgcccgcttgttcctgaagtgccagtaaagcgccggctgctgaacccccaaccgttccgccagtttgcgtgtcgtcagaccgtctacgccgacctcgttcaacaggtccagggcggcacggatcactgtattcggctgcaactttgtcatgattgacactttatcactgataaacataatatgtccaccaacttatcagtgataaagaatccgcgcgttcaatcggaccagcggaggctggtccggaggccagacgtgaaacccaacatacccctgatcgtaattctgagcactgtcgcgctcgacgctgtcggcatcggcctgattatgccggtgctgccgggcctcctgcgcgatctggttcactcgaacgacgtcaccgcccactatggcattctgctggcgctgtatgcgttggtgcaatttgcctgcgcacctgtgctgggcgcgctgtcggatcgtttcgggcggcggccaatcttgctcgtctcgctggccggcgccactgtcgactacgccatcatggcgacagcgcctttcctttgggttctctatatcgggcggatcgtggccggcatcaccggggcgactggggcggtagccggcgcttatattgccgatatcactgatggcgatgagcgcgcgcggcacttcggcttcatgagcgcctgtttcgggttcgggatggtcgcgggacctgtgctcggtgggctgatgggcggtttctccccccacgctccgttcttcgccgcggcagccttgaacggcctcaatttcctgacgggctgtttccttttgccggagtcgcacaaaggcgaacgccggccgttacgccgggaggctctcaacccgctcgcttcgttccggtgggcccggggcatgaccgtcgtcgccgccctgatggcggtcttcttcatcatgcaacttgtcggacaggtgccggccgcgctttgggtcattttcggcgaggatcgctttcactgggacgcgaccacgatcggcatttcgcttgccgcatttggcattctgcattcactcgcccaggcaatgatcaccggccctgtagccgcccggctcggcgaaaggcgggcactcatgctcggaatgattgccgacggcacaggctacatcctgcttgccttcgcgacacggggatggatggcgttcccgatcatggtcctgcttgcttcgggtggcatcggaatgccggcgctgcaagcaatgttgtccaggcaggtggatgaggaacgtcaggggcagctgcaaggctcactggcggcgctcaccagcctgacctcgatcgtcggacccctcctcttcacggcgatctatgcggcttctataacaacgtggaacgggtgggcatggattgcaggcgctgccctctacttgctctgcctgccggcgctgcgtcgcgggctttggagcggcgcagggcaacgagccgatcgctgatcgtggaaacgataggcctatgccatgcgggtcaaggcgacttccggcaagctatacgcgccctagaattgtcaattttaatcctctgtttatcggcagttcgtagagcgcgccgtgcgtcccgagcgatactgagcgaagcaagtgcgtcgagcagtgcccgcttgttcctgaaatgccagtaaagcgctggctgctgaacccccagccggaactgaccccacaaggccctagcgtttgcaatgcaccaggtcatcattgacccaggcgtgttccaccaggccgctgcctcgcaactcttcgcaggcttcgccgacctgctcgcgccacttcttcacgcgggtggaatccgatccgcacatgaggcggaaggtttccagcttgagcgggtacggctcccggtgcgagctgaaatagtcgaacatccgtcgggccgtcggcgacagcttgcggtacttctcccatatgaatttcgtgtagtggtcgccagcaaacagcacgacgatttcctcgtcgatcaggacctggcaacgggacgttttcttgccacggtccaggacgcggaagcggtgcagcagcgacaccgattccaggtgcccaacgcggtcggacgtgaagcccatcgccgtcgcctgtaggcgcgacaggcattcctcggccttcgtgtaataccggccattgatcgaccagcccaggtcctggcaaagctcgtagaacgtgaaggtgatcggctcgccgataggggtgcgcttcgcgtactccaacacctgctgccacaccagttcgtcatcgtcggcccgcagctcgacgccggtgtaggtgatcttcacgtccttgttgacgtggaaaatgaccttgttttgcagcgcctcgcgcgggattttcttgttgcgcgtggtgaacagggcagagcgggccgtgtcgtttggcatcgctcgcatcgtgtccggccacggcgcaatatcgaacaaggaaagctgcatttccttgatctgctgcttcgtgtgtttcagcaacgcggcctgcttggcctcgctgacctgttttgccaggtcctcgccggcggtttttcgcttcttggtcgtcatagttcctcgcgtgtcgatggtcatcgacttcgccaaacctgccgcctcctgttcgagacgacgcgaacgctccacggcggccgatggcgcgggcagggcagggggagccagttgcacgctgtcgcgctcgatcttggccgtagcttgctggaccatcgagccgacggactggaaggtttcgcggggcgcacgcatgacggtgcggcttgcgatggtttcggcatcctcggcggaaaaccccgcgtcgatcagttcttgcctgtatgccttccggtcaaacgtccgattcattcaccctccttgcgggattgccccgactcacgccggggcaatgtgcccttattcctgatttgacccgcctggtgccttggtgtccagataatccaccttatcggcaatgaagtcggtcccgtagaccgtctggccgtccttctcgtacttggtattccgaatcttgccctgcacgaataccagctccgcgaagtcgctcttcttgatggagcgcatggggacgtgcttggcaatcacgcgcaccccccggccgttttagcggctaaaaaagtcatggctctgccctcgggcggaccacgcccatcatgaccttgccaagctcgtcctgcttctcttcgatcttcgccagcagggcgaggatcgtggcatcaccgaaccgcgccgtgcgcgggtcgtcggtgagccagagtttcagcaggccgcccaggcggcccaggtcgccattgatgcgggccagctcgcggacgtgctcatagtccacgacgcccgtgattttgtagccctggccgacggccagcaggtaggcctacaggctcatgccggccgccgccgccttttcctcaatcgctcttcgttcgtctggaaggcagtacaccttgataggtgggctgcccttcctggttggcttggtttcatcagccatccgcttgccctcatctgttacgccggcggtagccggccagcctcgcagagcaggattcccgttgagcaccgccaggtgcgaataagggacagtgaagaaggaacacccgctcgcgggtgggcctacttcacctatcctgcccggctgacgccgttggatacaccaaggaaagtctacacgaaccctttggcaaaatcctgtatatcgtgcgaaaaaggatggatataccgaaaaaatcgctataatgaccccgaagcagggttatgcagcggaaaagatccgtc-3´

**pCM80-3028, the sequences in red encode the gene *META1_3028***

5´-

gaccctttccgacgctcaccgggctggttgccctcgccgctgggctggcggccgtctatggccctgcaaacgcgccagaaacgccgtcgaagccgtgtgcgagacaccgcggccgccggcgttgtggatacctcgcggaaaacttggccctcactgacagatgaggggcggacgttgacacttgaggggccgactcacccggcgcggcgttgacagatgaggggcaggctcgatttcggccggcgacgtggagctggccagcctcgcaaatcggcgaaaacgcctgattttacgcgagtttcccacagatgatgtggacaagcctggggataagtgccctgcggtattgacacttgaggggcgcgactactgacagatgaggggcgcgatccttgacacttgaggggcagagtgctgacagatgaggggcgcacctattgacatttgaggggctgtccacaggcagaaaatccagcatttgcaagggtttccgcccgtttttcggccaccgctaacctgtcttttaacctgcttttaaaccaatatttataaaccttgtttttaaccagggctgcgccctgtgcgcgtgaccgcgcacgccgaaggggggtgcccccccttctcgaaccctcccggcccgctaacgcgggcctcccatccccccaggggctgcgcccctcggccgcgaacggcctcaccccaaaaatggcagccaagctgaccacttctgcgctcggcccttccggctggctggtttattgctgataaatctggagccggtgagcgtgggtctcgcggtatcattgcagcactggggccagatggtaagccctcccgtatcgtagttatctacacgacggggagtcaggcaactatggatgaacgaaatagacagatcgctgagataggtgcctcactgattaagcattggtaactgtcagaccaagtttactcatatatactttagattgatttaaaacttcatttttaatttaaaaggatctaggtgaagatcctttttgataatctcatgaccaaaatcccttaacgtgagttttcgttccactgagcgtcagaccccgtagaaaagatcaaaggatcttcttgagatcctttttttctgcgcgtaatctgctgcttgcaaacaaaaaaaccaccgctaccagcggtggtttgtttgccggatcaagagctaccaactctttttccgaaggtaactggcttcagcagagcgcagataccaaatactgtccttctagtgtagccgtagttaggccaccacttcaagaactctgtagcaccgcctacatacctcgctctgctaatcctgttaccagtggctgctgccagtggcgataagtcgtgtcttaccgggttggactcaagacgatagttaccggataaggcgcagcggtcgggctgaacggggggttcgtgcacacagcccagcttggagcgaacgacctacaccgaactgagatacctacagcgtgagctatgagaaagcgccacgcttcccgaagggagaaaggcggacaggtatccggtaagcggcagggtcggaacaggagagcgcacgagggagcttccagggggaaacgcctggtatctttatagtcctgtcgggtttcgccacctctgacttgagcgtcgatttttgtgatgctcgtcaggggggcggagcctatggaaaaacgccagcaacgcggcctttttacggttcctggccttttgctggccttttgctcacatgttctttcctgcgttatcccctgattctgtggataaccgtattaccgcctttgagtgagctgataccgctcgccgcagccgaacgaccgagcgcagcgagtcagtgagcgaggaagcggaagagcgcccaatacgcaaaccgcctctccccgcgcgttggccgattcattaatgcagctggcacgacaggtttcccgactggaaagcgggcagtgagcgcaacgcaattaatgtgagttagctcactcattaggcaccccaggctttacactttatgcttccggctcgtatgttgtgtggaattgtgagcggataacaatttcacacaggaaacagctatgaccatgattacgccaagctagcttcccgcttggtcgggccgcttcgcgagggcccgttgacgacaacggtgcgatgggtcccggccccggtcaagacgatgccaatacgttgcgacactacgccttggcacttttagaattgccttatcgtcctgataagaaatgtccgaccagctaaagacatcgcgtccaatcaaagcctagaaaatataggcgaagggacgctaataagtctttcataagaccgcgcaaatctaaaaatatccttagattcacgatgcggcacttcggatgacttccgagcgagcctggaacctcagaaaaacgtctgagagataccgcggatctcacacaggaaacagctatgaccatgattacgccaagcttGTGGACGGCATCATCCAAGGCCTGTCGAACTTCCGGGGAACGGTCTTTCCGGGTCAGCAGCAGATGTATCAGCGGCTGGTGCGCGACGGTCAGCAGCCTAAGGCGCTCATGATCGCCTGCGCCGATTCCCGCGTCTCGCCCGAGCACATCACGCAGTCCGGTCCGGGCGACCTGTTCGTATGCCGGAATGCCGGCAACATCGTCCCGCCCTTCTCGCAGCAGAACGGCGGCGTGTCCTCGGCGATCGAGTACGCGGTCGTCGCCCTCGGCGTGCTGGACATCGTGATCTGCGGTCATTCCGACTGCGGCGCCATGAAGGGGCTCATGAATCCCGAGGCGCTGGGCAACATGCCCAACGTGGCGGCGTGGCTGCGCCACAGCCATGCGGCGAAACAGATCGTCTGCGAGGCCTATCCCGGGGGCATGGACCCGAAGGAGCGCCACCGCGCCGTCGCCCTCGAGAACGTCGTGGTGCAACTCAACCATCTGCGCACCCACCCGAGCGTGGCGACGGCTCTGGCCCGCGGCAAGCTGCGTCTGCACGGCTGGTTCTTCGAAATCGAGAGCGGCCAGGTGCTCGCCTATTGCGGCGAGCGCGGCCGCTTCATCCCCATCGACGAAGCCACGGGTGTCCCCGTCGCCCAGGCTTCGGCGTCGCGGATCGCCACACCGGAATTCGCCGGCCCCCATGCTGGTCCCCATGCCAGCCCCCAAGCCGGGCCCGAACCCATCCCCCACGCTATCGCAGCGGAGTAGggatccccgggtaccgagctcgaattcactggccgtcgttttacaacgtcgtgactgggaaaaccctggcgttacccaacttaatcgccttgcagcacatccccctttcgccagctggcgtaatagcgaagaggcccgcaccgatcgcccttcccaacagttgcgcagcctgaatggcgaatggcgcctgatgcggtattttctccttacgcatctgtgcggtatttcacaccgcatatggtgcactctcagtacaatctgctctgatgccgcatagttaagccagccccgacacccgccaacacccgctgacgcgccctgacgggcttgtctgctcccggcatccgcttacagacaagctgtgaccgtctccgggagctgcatgtgtcagaggttttcaccgtcatcaccgaaacgcgcgagacgaaagggcctcgtgatacgcctatttttataggttaatgtcatgataataatggtttcttagcaccctttctcggtccttcaacgttcctgacaacgagcctccttttcgccaatccatcgacaatcaccgcgagtccctgctcgaacgctgcgtccggaccggcttcgtcgaaggcgtctatcgcggcccgcaacagcggcgagagcggagcctgttcaacggtgccgccgcgctcgccggcatcgctgtcgccggcctgctcctcaagcacggccccaacagtgaagtagctgattgtcatcagcgcattgacggcgtccccggccgaaaaacccgcctcgcagaggaagcgaagctgcgcgtcggccgtttccatctgcggtgcgcccggtcgcgtgccggcatggatgcgcgcgccatcgcggtaggcgagcagcgcctgcctgaagctgcgggcattcccgatcagaaatgagcgccagtcgtcgtcggctctcggcaccgaatgcgtatgattctccgccagcatggcttcggccagtgcgtcgagcagcgcccgcttgttcctgaagtgccagtaaagcgccggctgctgaacccccaaccgttccgccagtttgcgtgtcgtcagaccgtctacgccgacctcgttcaacaggtccagggcggcacggatcactgtattcggctgcaactttgtcatgattgacactttatcactgataaacataatatgtccaccaacttatcagtgataaagaatccgcgcgttcaatcggaccagcggaggctggtccggaggccagacgtgaaacccaacatacccctgatcgtaattctgagcactgtcgcgctcgacgctgtcggcatcggcctgattatgccggtgctgccgggcctcctgcgcgatctggttcactcgaacgacgtcaccgcccactatggcattctgctggcgctgtatgcgttggtgcaatttgcctgcgcacctgtgctgggcgcgctgtcggatcgtttcgggcggcggccaatcttgctcgtctcgctggccggcgccactgtcgactacgccatcatggcgacagcgcctttcctttgggttctctatatcgggcggatcgtggccggcatcaccggggcgactggggcggtagccggcgcttatattgccgatatcactgatggcgatgagcgcgcgcggcacttcggcttcatgagcgcctgtttcgggttcgggatggtcgcgggacctgtgctcggtgggctgatgggcggtttctccccccacgctccgttcttcgccgcggcagccttgaacggcctcaatttcctgacgggctgtttccttttgccggagtcgcacaaaggcgaacgccggccgttacgccgggaggctctcaacccgctcgcttcgttccggtgggcccggggcatgaccgtcgtcgccgccctgatggcggtcttcttcatcatgcaacttgtcggacaggtgccggccgcgctttgggtcattttcggcgaggatcgctttcactgggacgcgaccacgatcggcatttcgcttgccgcatttggcattctgcattcactcgcccaggcaatgatcaccggccctgtagccgcccggctcggcgaaaggcgggcactcatgctcggaatgattgccgacggcacaggctacatcctgcttgccttcgcgacacggggatggatggcgttcccgatcatggtcctgcttgcttcgggtggcatcggaatgccggcgctgcaagcaatgttgtccaggcaggtggatgaggaacgtcaggggcagctgcaaggctcactggcggcgctcaccagcctgacctcgatcgtcggacccctcctcttcacggcgatctatgcggcttctataacaacgtggaacgggtgggcatggattgcaggcgctgccctctacttgctctgcctgccggcgctgcgtcgcgggctttggagcggcgcagggcaacgagccgatcgctgatcgtggaaacgataggcctatgccatgcgggtcaaggcgacttccggcaagctatacgcgccctagaattgtcaattttaatcctctgtttatcggcagttcgtagagcgcgccgtgcgtcccgagcgatactgagcgaagcaagtgcgtcgagcagtgcccgcttgttcctgaaatgccagtaaagcgctggctgctgaacccccagccggaactgaccccacaaggccctagcgtttgcaatgcaccaggtcatcattgacccaggcgtgttccaccaggccgctgcctcgcaactcttcgcaggcttcgccgacctgctcgcgccacttcttcacgcgggtggaatccgatccgcacatgaggcggaaggtttccagcttgagcgggtacggctcccggtgcgagctgaaatagtcgaacatccgtcgggccgtcggcgacagcttgcggtacttctcccatatgaatttcgtgtagtggtcgccagcaaacagcacgacgatttcctcgtcgatcaggacctggcaacgggacgttttcttgccacggtccaggacgcggaagcggtgcagcagcgacaccgattccaggtgcccaacgcggtcggacgtgaagcccatcgccgtcgcctgtaggcgcgacaggcattcctcggccttcgtgtaataccggccattgatcgaccagcccaggtcctggcaaagctcgtagaacgtgaaggtgatcggctcgccgataggggtgcgcttcgcgtactccaacacctgctgccacaccagttcgtcatcgtcggcccgcagctcgacgccggtgtaggtgatcttcacgtccttgttgacgtggaaaatgaccttgttttgcagcgcctcgcgcgggattttcttgttgcgcgtggtgaacagggcagagcgggccgtgtcgtttggcatcgctcgcatcgtgtccggccacggcgcaatatcgaacaaggaaagctgcatttccttgatctgctgcttcgtgtgtttcagcaacgcggcctgcttggcctcgctgacctgttttgccaggtcctcgccggcggtttttcgcttcttggtcgtcatagttcctcgcgtgtcgatggtcatcgacttcgccaaacctgccgcctcctgttcgagacgacgcgaacgctccacggcggccgatggcgcgggcagggcagggggagccagttgcacgctgtcgcgctcgatcttggccgtagcttgctggaccatcgagccgacggactggaaggtttcgcggggcgcacgcatgacggtgcggcttgcgatggtttcggcatcctcggcggaaaaccccgcgtcgatcagttcttgcctgtatgccttccggtcaaacgtccgattcattcaccctccttgcgggattgccccgactcacgccggggcaatgtgcccttattcctgatttgacccgcctggtgccttggtgtccagataatccaccttatcggcaatgaagtcggtcccgtagaccgtctggccgtccttctcgtacttggtattccgaatcttgccctgcacgaataccagctccgcgaagtcgctcttcttgatggagcgcatggggacgtgcttggcaatcacgcgcaccccccggccgttttagcggctaaaaaagtcatggctctgccctcgggcggaccacgcccatcatgaccttgccaagctcgtcctgcttctcttcgatcttcgccagcagggcgaggatcgtggcatcaccgaaccgcgccgtgcgcgggtcgtcggtgagccagagtttcagcaggccgcccaggcggcccaggtcgccattgatgcgggccagctcgcggacgtgctcatagtccacgacgcccgtgattttgtagccctggccgacggccagcaggtaggcctacaggctcatgccggccgccgccgccttttcctcaatcgctcttcgttcgtctggaaggcagtacaccttgataggtgggctgcccttcctggttggcttggtttcatcagccatccgcttgccctcatctgttacgccggcggtagccggccagcctcgcagagcaggattcccgttgagcaccgccaggtgcgaataagggacagtgaagaaggaacacccgctcgcgggtgggcctacttcacctatcctgcccggctgacgccgttggatacaccaaggaaagtctacacgaaccctttggcaaaatcctgtatatcgtgcgaaaaaggatggatataccgaaaaaatcgctataatgaccccgaagcagggttatgcagcggaaaagatccgtc-3´

**pCM80-1261-1260, the sequences in red encode *META1_1261* and the sequences in blue encode *META1_1260***

gaccctttccgacgctcaccgggctggttgccctcgccgctgggctggcggccgtctatggccctgcaaacgcgccagaaacgccgtcgaagccgtgtgcgagacaccgcggccgccggcgttgtggatacctcgcggaaaacttggccctcactgacagatgaggggcggacgttgacacttgaggggccgactcacccggcgcggcgttgacagatgaggggcaggctcgatttcggccggcgacgtggagctggccagcctcgcaaatcggcgaaaacgcctgattttacgcgagtttcccacagatgatgtggacaagcctggggataagtgccctgcggtattgacacttgaggggcgcgactactgacagatgaggggcgcgatccttgacacttgaggggcagagtgctgacagatgaggggcgcacctattgacatttgaggggctgtccacaggcagaaaatccagcatttgcaagggtttccgcccgtttttcggccaccgctaacctgtcttttaacctgcttttaaaccaatatttataaaccttgtttttaaccagggctgcgccctgtgcgcgtgaccgcgcacgccgaaggggggtgcccccccttctcgaaccctcccggcccgctaacgcgggcctcccatccccccaggggctgcgcccctcggccgcgaacggcctcaccccaaaaatggcagccaagctgaccacttctgcgctcggcccttccggctggctggtttattgctgataaatctggagccggtgagcgtgggtctcgcggtatcattgcagcactggggccagatggtaagccctcccgtatcgtagttatctacacgacggggagtcaggcaactatggatgaacgaaatagacagatcgctgagataggtgcctcactgattaagcattggtaactgtcagaccaagtttactcatatatactttagattgatttaaaacttcatttttaatttaaaaggatctaggtgaagatcctttttgataatctcatgaccaaaatcccttaacgtgagttttcgttccactgagcgtcagaccccgtagaaaagatcaaaggatcttcttgagatcctttttttctgcgcgtaatctgctgcttgcaaacaaaaaaaccaccgctaccagcggtggtttgtttgccggatcaagagctaccaactctttttccgaaggtaactggcttcagcagagcgcagataccaaatactgtccttctagtgtagccgtagttaggccaccacttcaagaactctgtagcaccgcctacatacctcgctctgctaatcctgttaccagtggctgctgccagtggcgataagtcgtgtcttaccgggttggactcaagacgatagttaccggataaggcgcagcggtcgggctgaacggggggttcgtgcacacagcccagcttggagcgaacgacctacaccgaactgagatacctacagcgtgagctatgagaaagcgccacgcttcccgaagggagaaaggcggacaggtatccggtaagcggcagggtcggaacaggagagcgcacgagggagcttccagggggaaacgcctggtatctttatagtcctgtcgggtttcgccacctctgacttgagcgtcgatttttgtgatgctcgtcaggggggcggagcctatggaaaaacgccagcaacgcggcctttttacggttcctggccttttgctggccttttgctcacatgttctttcctgcgttatcccctgattctgtggataaccgtattaccgcctttgagtgagctgataccgctcgccgcagccgaacgaccgagcgcagcgagtcagtgagcgaggaagcggaagagcgcccaatacgcaaaccgcctctccccgcgcgttggccgattcattaatgcagctggcacgacaggtttcccgactggaaagcgggcagtgagcgcaacgcaattaatgtgagttagctcactcattaggcaccccaggctttacactttatgcttccggctcgtatgttgtgtggaattgtgagcggataacaatttcacacaggaaacagctatgaccatgattacgccaagctagcttcccgcttggtcgggccgcttcgcgagggcccgttgacgacaacggtgcgatgggtcccggccccggtcaagacgatgccaatacgttgcgacactacgccttggcacttttagaattgccttatcgtcctgataagaaatgtccgaccagctaaagacatcgcgtccaatcaaagcctagaaaatataggcgaagggacgctaataagtctttcataagaccgcgcaaatctaaaaatatccttagattcacgatgcggcacttcggatgacttccgagcgagcctggaacctcagaaaaacgtctgagagataccgcggatctcacacaggaaacagctatgaccatgattacgccaagcttATGCGCTCGAAACGCTCCGAAATCGTCGACCTGCTGGTGCCCTTGCGGCGCTACGCCCGGTCGCTCACGCGGGATTCGCTGAAAGCGGATGACCTCGTGCACGATACCATGGTTCGCGCCCTCGAATCGCGCGCGAACCTGAGGCCGGACACCAACCTGCGCACCTGGATGATGACGGTGCTGCACAACGTCTTCATCGATGAGCAGCGCCGCAAACGCGTCGAGGCACGCCACGCCGATGTGCTGGTGCAGCTCTCGGACGACGTGGCACCGCCGGCCCAAGAGGGGCAGGTGCGGCTCATGCAGATCCGCAAGGCGTTCGAAGGACTGCCCGAGGAGCAGCGCGCCGCGCTCCACCTCGTGACGCTCGAAGGCATGGCTTATGCCGACGCGGCGGCCGTGCTCGGTATCCCGATCGGAACCCTGATGTCGCGGCTCGGCCGCGGCCGTGCGGCCCTACGCGCCTTCGAGGAAGGCGGCCGCAAGGGCAAGGCCACCGCCGGATCCTCCGACGATGGCCCCGACCGTGGCCGTGCACATCTGCGCGTCGTCGGTGGGAATTGAtgaccctgttgagccgttaacaacgaagacaccggaaaactaagaggttcgacggtgggcaagcagggcagtcggagtgggctgATGGTCGATCCGGTTACGGAAACCGAACTCATCGCGTATGTCGATGGTGAACTCGAGCCGATGCGTCGCGTCGAAGTCGAGGCGCATCTCGCGCGCAATCCTCAGGACGCGCTGCAGGTCATGGCCGACCTGCGCGACCGCGACGCCCTGCGTGAAGCCTTCCTGCCGGTGCCGAGCCTGCCGCCGGAGCGGCTGCGCTCCAGTGCTCGCCGGATCGACCGCGCCTTCGCGTGGCAGCGGGTTCGCTCCCGCCTGCGCCGGGCGGCGGCGGTCGTGGTCCTGGTCGGAGCGGGCTGGCTCGCCCACACCGAGAGCGGCAGCTTCGGCGTTCCCGGCTCCATCGCCTCGCCGATCGATCCGTCCCTAGCCGAGGAAGCGAAGCAGGCCCGCGAGGCCGTGCAGATCCGCGCCCGCACGACCAGTCAGCCCATGGCCTCGGCCTACGATCCGGCCGACCTCGAAGCTGCGACCGGTGTTGATCTGCCGCCTCTGCCGAAGGGGTGGATCGTCCGGGACGTGCAGATCTTCCCGAGCCGGAGCGGCACCGGGATCGAGGTCTCGATCCAGTCGGCCGAGATCGGCGAACTCGCGCTGTTCGCCAAGCACAGCAGCGGCGCGCCCCTGTCCGAAGGGACGGAGCCGGTTGCACGCTCGAGCGACGGGACGACGGCCTATTGGCGGACCGGCGGCACCGCCTACGCACTGAGCGGCGCGCGCGACGATCCGGCCCTGCACGCCGCCGCCGCCCGCCTTGCCGCGCTCCGGAAGTAAggatccccgggtaccgagctcgaattcactggccgtcgttttacaacgtcgtgactgggaaaaccctggcgttacccaacttaatcgccttgcagcacatccccctttcgccagctggcgtaatagcgaagaggcccgcaccgatcgcccttcccaacagttgcgcagcctgaatggcgaatggcgcctgatgcggtattttctccttacgcatctgtgcggtatttcacaccgcatatggtgcactctcagtacaatctgctctgatgccgcatagttaagccagccccgacacccgccaacacccgctgacgcgccctgacgggcttgtctgctcccggcatccgcttacagacaagctgtgaccgtctccgggagctgcatgtgtcagaggttttcaccgtcatcaccgaaacgcgcgagacgaaagggcctcgtgatacgcctatttttataggttaatgtcatgataataatggtttcttagcaccctttctcggtccttcaacgttcctgacaacgagcctccttttcgccaatccatcgacaatcaccgcgagtccctgctcgaacgctgcgtccggaccggcttcgtcgaaggcgtctatcgcggcccgcaacagcggcgagagcggagcctgttcaacggtgccgccgcgctcgccggcatcgctgtcgccggcctgctcctcaagcacggccccaacagtgaagtagctgattgtcatcagcgcattgacggcgtccccggccgaaaaacccgcctcgcagaggaagcgaagctgcgcgtcggccgtttccatctgcggtgcgcccggtcgcgtgccggcatggatgcgcgcgccatcgcggtaggcgagcagcgcctgcctgaagctgcgggcattcccgatcagaaatgagcgccagtcgtcgtcggctctcggcaccgaatgcgtatgattctccgccagcatggcttcggccagtgcgtcgagcagcgcccgcttgttcctgaagtgccagtaaagcgccggctgctgaacccccaaccgttccgccagtttgcgtgtcgtcagaccgtctacgccgacctcgttcaacaggtccagggcggcacggatcactgtattcggctgcaactttgtcatgattgacactttatcactgataaacataatatgtccaccaacttatcagtgataaagaatccgcgcgttcaatcggaccagcggaggctggtccggaggccagacgtgaaacccaacatacccctgatcgtaattctgagcactgtcgcgctcgacgctgtcggcatcggcctgattatgccggtgctgccgggcctcctgcgcgatctggttcactcgaacgacgtcaccgcccactatggcattctgctggcgctgtatgcgttggtgcaatttgcctgcgcacctgtgctgggcgcgctgtcggatcgtttcgggcggcggccaatcttgctcgtctcgctggccggcgccactgtcgactacgccatcatggcgacagcgcctttcctttgggttctctatatcgggcggatcgtggccggcatcaccggggcgactggggcggtagccggcgcttatattgccgatatcactgatggcgatgagcgcgcgcggcacttcggcttcatgagcgcctgtttcgggttcgggatggtcgcgggacctgtgctcggtgggctgatgggcggtttctccccccacgctccgttcttcgccgcggcagccttgaacggcctcaatttcctgacgggctgtttccttttgccggagtcgcacaaaggcgaacgccggccgttacgccgggaggctctcaacccgctcgcttcgttccggtgggcccggggcatgaccgtcgtcgccgccctgatggcggtcttcttcatcatgcaacttgtcggacaggtgccggccgcgctttgggtcattttcggcgaggatcgctttcactgggacgcgaccacgatcggcatttcgcttgccgcatttggcattctgcattcactcgcccaggcaatgatcaccggccctgtagccgcccggctcggcgaaaggcgggcactcatgctcggaatgattgccgacggcacaggctacatcctgcttgccttcgcgacacggggatggatggcgttcccgatcatggtcctgcttgcttcgggtggcatcggaatgccggcgctgcaagcaatgttgtccaggcaggtggatgaggaacgtcaggggcagctgcaaggctcactggcggcgctcaccagcctgacctcgatcgtcggacccctcctcttcacggcgatctatgcggcttctataacaacgtggaacgggtgggcatggattgcaggcgctgccctctacttgctctgcctgccggcgctgcgtcgcgggctttggagcggcgcagggcaacgagccgatcgctgatcgtggaaacgataggcctatgccatgcgggtcaaggcgacttccggcaagctatacgcgccctagaattgtcaattttaatcctctgtttatcggcagttcgtagagcgcgccgtgcgtcccgagcgatactgagcgaagcaagtgcgtcgagcagtgcccgcttgttcctgaaatgccagtaaagcgctggctgctgaacccccagccggaactgaccccacaaggccctagcgtttgcaatgcaccaggtcatcattgacccaggcgtgttccaccaggccgctgcctcgcaactcttcgcaggcttcgccgacctgctcgcgccacttcttcacgcgggtggaatccgatccgcacatgaggcggaaggtttccagcttgagcgggtacggctcccggtgcgagctgaaatagtcgaacatccgtcgggccgtcggcgacagcttgcggtacttctcccatatgaatttcgtgtagtggtcgccagcaaacagcacgacgatttcctcgtcgatcaggacctggcaacgggacgttttcttgccacggtccaggacgcggaagcggtgcagcagcgacaccgattccaggtgcccaacgcggtcggacgtgaagcccatcgccgtcgcctgtaggcgcgacaggcattcctcggccttcgtgtaataccggccattgatcgaccagcccaggtcctggcaaagctcgtagaacgtgaaggtgatcggctcgccgataggggtgcgcttcgcgtactccaacacctgctgccacaccagttcgtcatcgtcggcccgcagctcgacgccggtgtaggtgatcttcacgtccttgttgacgtggaaaatgaccttgttttgcagcgcctcgcgcgggattttcttgttgcgcgtggtgaacagggcagagcgggccgtgtcgtttggcatcgctcgcatcgtgtccggccacggcgcaatatcgaacaaggaaagctgcatttccttgatctgctgcttcgtgtgtttcagcaacgcggcctgcttggcctcgctgacctgttttgccaggtcctcgccggcggtttttcgcttcttggtcgtcatagttcctcgcgtgtcgatggtcatcgacttcgccaaacctgccgcctcctgttcgagacgacgcgaacgctccacggcggccgatggcgcgggcagggcagggggagccagttgcacgctgtcgcgctcgatcttggccgtagcttgctggaccatcgagccgacggactggaaggtttcgcggggcgcacgcatgacggtgcggcttgcgatggtttcggcatcctcggcggaaaaccccgcgtcgatcagttcttgcctgtatgccttccggtcaaacgtccgattcattcaccctccttgcgggattgccccgactcacgccggggcaatgtgcccttattcctgatttgacccgcctggtgccttggtgtccagataatccaccttatcggcaatgaagtcggtcccgtagaccgtctggccgtccttctcgtacttggtattccgaatcttgccctgcacgaataccagctccgcgaagtcgctcttcttgatggagcgcatggggacgtgcttggcaatcacgcgcaccccccggccgttttagcggctaaaaaagtcatggctctgccctcgggcggaccacgcccatcatgaccttgccaagctcgtcctgcttctcttcgatcttcgccagcagggcgaggatcgtggcatcaccgaaccgcgccgtgcgcgggtcgtcggtgagccagagtttcagcaggccgcccaggcggcccaggtcgccattgatgcgggccagctcgcggacgtgctcatagtccacgacgcccgtgattttgtagccctggccgacggccagcaggtaggcctacaggctcatgccggccgccgccgccttttcctcaatcgctcttcgttcgtctggaaggcagtacaccttgataggtgggctgcccttcctggttggcttggtttcatcagccatccgcttgccctcatctgttacgccggcggtagccggccagcctcgcagagcaggattcccgttgagcaccgccaggtgcgaataagggacagtgaagaaggaacacccgctcgcgggtgggcctacttcacctatcctgcccggctgacgccgttggatacaccaaggaaagtctacacgaaccctttggcaaaatcctgtatatcgtgcgaaaaaggatggatataccgaaaaaatcgctataatgaccccgaagcagggttatgcagcggaaaagatccgtc

**pCM80-3028-3027, the sequences in red encode the gene META1_3028 and the sequences in blue encode the gene META1_3027**

5´-

gaccctttccgacgctcaccgggctggttgccctcgccgctgggctggcggccgtctatggccctgcaaacgcgccagaaacgccgtcgaagccgtgtgcgagacaccgcggccgccggcgttgtggatacctcgcggaaaacttggccctcactgacagatgaggggcggacgttgacacttgaggggccgactcacccggcgcggcgttgacagatgaggggcaggctcgatttcggccggcgacgtggagctggccagcctcgcaaatcggcgaaaacgcctgattttacgcgagtttcccacagatgatgtggacaagcctggggataagtgccctgcggtattgacacttgaggggcgcgactactgacagatgaggggcgcgatccttgacacttgaggggcagagtgctgacagatgaggggcgcacctattgacatttgaggggctgtccacaggcagaaaatccagcatttgcaagggtttccgcccgtttttcggccaccgctaacctgtcttttaacctgcttttaaaccaatatttataaaccttgtttttaaccagggctgcgccctgtgcgcgtgaccgcgcacgccgaaggggggtgcccccccttctcgaaccctcccggcccgctaacgcgggcctcccatccccccaggggctgcgcccctcggccgcgaacggcctcaccccaaaaatggcagccaagctgaccacttctgcgctcggcccttccggctggctggtttattgctgataaatctggagccggtgagcgtgggtctcgcggtatcattgcagcactggggccagatggtaagccctcccgtatcgtagttatctacacgacggggagtcaggcaactatggatgaacgaaatagacagatcgctgagataggtgcctcactgattaagcattggtaactgtcagaccaagtttactcatatatactttagattgatttaaaacttcatttttaatttaaaaggatctaggtgaagatcctttttgataatctcatgaccaaaatcccttaacgtgagttttcgttccactgagcgtcagaccccgtagaaaagatcaaaggatcttcttgagatcctttttttctgcgcgtaatctgctgcttgcaaacaaaaaaaccaccgctaccagcggtggtttgtttgccggatcaagagctaccaactctttttccgaaggtaactggcttcagcagagcgcagataccaaatactgtccttctagtgtagccgtagttaggccaccacttcaagaactctgtagcaccgcctacatacctcgctctgctaatcctgttaccagtggctgctgccagtggcgataagtcgtgtcttaccgggttggactcaagacgatagttaccggataaggcgcagcggtcgggctgaacggggggttcgtgcacacagcccagcttggagcgaacgacctacaccgaactgagatacctacagcgtgagctatgagaaagcgccacgcttcccgaagggagaaaggcggacaggtatccggtaagcggcagggtcggaacaggagagcgcacgagggagcttccagggggaaacgcctggtatctttatagtcctgtcgggtttcgccacctctgacttgagcgtcgatttttgtgatgctcgtcaggggggcggagcctatggaaaaacgccagcaacgcggcctttttacggttcctggccttttgctggccttttgctcacatgttctttcctgcgttatcccctgattctgtggataaccgtattaccgcctttgagtgagctgataccgctcgccgcagccgaacgaccgagcgcagcgagtcagtgagcgaggaagcggaagagcgcccaatacgcaaaccgcctctccccgcgcgttggccgattcattaatgcagctggcacgacaggtttcccgactggaaagcgggcagtgagcgcaacgcaattaatgtgagttagctcactcattaggcaccccaggctttacactttatgcttccggctcgtatgttgtgtggaattgtgagcggataacaatttcacacaggaaacagctatgaccatgattacgccaagctagcttcccgcttggtcgggccgcttcgcgagggcccgttgacgacaacggtgcgatgggtcccggccccggtcaagacgatgccaatacgttgcgacactacgccttggcacttttagaattgccttatcgtcctgataagaaatgtccgaccagctaaagacatcgcgtccaatcaaagcctagaaaatataggcgaagggacgctaataagtctttcataagaccgcgcaaatctaaaaatatccttagattcacgatgcggcacttcggatgacttccgagcgagcctggaacctcagaaaaacgtctgagagataccgcggatctcacacaggaaacagctatgaccatgattacgccaagcttGTGGACGGCATCATCCAAGGCCTGTCGAACTTCCGGGGAACGGTCTTTCCGGGTCAGCAGCAGATGTATCAGCGGCTGGTGCGCGACGGTCAGCAGCCTAAGGCGCTCATGATCGCCTGCGCCGATTCCCGCGTCTCGCCCGAGCACATCACGCAGTCCGGTCCGGGCGACCTGTTCGTATGCCGGAATGCCGGCAACATCGTCCCGCCCTTCTCGCAGCAGAACGGCGGCGTGTCCTCGGCGATCGAGTACGCGGTCGTCGCCCTCGGCGTGCTGGACATCGTGATCTGCGGTCATTCCGACTGCGGCGCCATGAAGGGGCTCATGAATCCCGAGGCGCTGGGCAACATGCCCAACGTGGCGGCGTGGCTGCGCCACAGCCATGCGGCGAAACAGATCGTCTGCGAGGCCTATCCCGGGGGCATGGACCCGAAGGAGCGCCACCGCGCCGTCGCCCTCGAGAACGTCGTGGTGCAACTCAACCATCTGCGCACCCACCCGAGCGTGGCGACGGCTCTGGCCCGCGGCAAGCTGCGTCTGCACGGCTGGTTCTTCGAAATCGAGAGCGGCCAGGTGCTCGCCTATTGCGGCGAGCGCGGCCGCTTCATCCCCATCGACGAAGCCACGGGTGTCCCCGTCGCCCAGGCTTCGGCGTCGCGGATCGCCACACCGGAATTCGCCGGCCCCCATGCTGGTCCCCATGCCAGCCCCCAAGCCGGGCCCGAACCCATCCCCCACGCTATCGCAGCGGAGTAGatttttgctATGCGCGCCTCCCTCGCCCGGCTGATGCCATCGACCATCGGCCGGGACCTTCCGGCCTCCTTCGTCGTCTTCCTCGTGGCGATGCCTCTCTGCATGGGCATCGCTATGGCCTCCGGTGTGCCCGCCGAGCGCGGGCTCATCACCGGCATCATCGGCGGTATCGTCGTCGGCTTCCTCGCCGGCTCGCCGCTTCAGGTCAGCGGCCCGGCCGCGGGCCTTGCCGTCATCGTCTTCGAGTTCGTGCGTGAGCACGGCATCGACGCGCTCGGGCCGGTCCTCGTCGCGGCGGGCGCCATCCAGCTTCTCGCCGGGGCGCTCCGGGTCGGCGGCTGGTTCAGGGCGATCTCGCCGGCGGTCGTGCACGGCATGCTCGCCGGCATCGGCATCCTGATCGTGCTGGCCCAGATCCACGTGCTGACCGACGCCCTGCCCAAGGCCAGCGGCCTCGACAACCTCGTCGCCATCCCCGCCGCGTTCTTCAACTTCGTCTCCGGTCCGGACGGCAACCGCCCCGGGGCCGTGATCGTCGGCCTTGCCACCATCGTTGCGATGATCGGCTGGGAGAAGATCCGGCCGGCTAAGCTCAAGCTGATTCCCGGTGCGCTCATGGGCGTGCTGGCCGGCACCGTCGTGGCCGTCGTCGGCAGCATGGACGTCAAGCGCGTCGAGGTGCCGGAGAACATCTTCTCCGCCGTGACCATGCCGGGAATGGGCGACTGGAGCCGCTTGGCCGAGCCGGCGATGATCCTGATGGCGGTCACCCTCGCGGTCATCGCCAGCGCGGAGAGCCTGCTCTCGGCAGCGGCGGTGGACCGGATGCATGACGGCCCGCGCACGCAGTACAACCGCGAACTCGGGGCGCAGGGCATCGGCAACATCCTCTGCGGCCTCGCGGGCGGCCTGCCGATGACCGGTGTGATTGTCCGGTCCTCGGCCAACGTCCAGGCGGGTGCGGCGACGCGCGCCTCCACCATCCTGCACGGGAGCTGGATCCTCGCCTTCCTGCTGGTCCTGCCGATGGTGCTGAAGGTCGTGCCGACCGCCTCCCTCGCCGGTATCCTCGTGGTGACGGGCTGGCGCCTCGTGAGCCCCGCCCACGCCTTCCACCTGCACGAGCGCTACGGCCTGCCCACCGCCGCGATCTGGCTCGCGACCATGGTGATGGTCGTCGCGACCGACCTGTTGACCGGTGTTCTCACGGGCCTTGCGCTCAGCCTCCTCCAGGTGATCCCGCACTACCTGCGTGGTCCGCTCAAGATCGAGGGCGGCGCGTCGCAGACGGTGCAAGGCGGAGCGCTTCAGGCGGTGCCGGAACTGCGGCTCTCGGGTTCGGCGACCTTCCTCCAACTGCCGCATCTCAATGCGGCGCTGGAGCGGACGCCGGAAGGCTCGCCGATCCGTCTCGCAGCACAGGATCTACGCCACGTCGATCACACCTGCCTGGAGATGATGCGGGAATGGGCGACGCGGCGGGCCAAGACCGGGTCGAGGGTCGAGGTCGTCGGCGGCGGCGCGAGCGGCCTGCATCACAGCCTCGCGATGGTCGCTCACGCCGCGCCGAAGGACTGAggatccccgggtaccgagctcgaattcactggccgtcgttttacaacgtcgtgactgggaaaaccctggcgttacccaacttaatcgccttgcagcacatccccctttcgccagctggcgtaatagcgaagaggcccgcaccgatcgcccttcccaacagttgcgcagcctgaatggcgaatggcgcctgatgcggtattttctccttacgcatctgtgcggtatttcacaccgcatatggtgcactctcagtacaatctgctctgatgccgcatagttaagccagccccgacacccgccaacacccgctgacgcgccctgacgggcttgtctgctcccggcatccgcttacagacaagctgtgaccgtctccgggagctgcatgtgtcagaggttttcaccgtcatcaccgaaacgcgcgagacgaaagggcctcgtgatacgcctatttttataggttaatgtcatgataataatggtttcttagcaccctttctcggtccttcaacgttcctgacaacgagcctccttttcgccaatccatcgacaatcaccgcgagtccctgctcgaacgctgcgtccggaccggcttcgtcgaaggcgtctatcgcggcccgcaacagcggcgagagcggagcctgttcaacggtgccgccgcgctcgccggcatcgctgtcgccggcctgctcctcaagcacggccccaacagtgaagtagctgattgtcatcagcgcattgacggcgtccccggccgaaaaacccgcctcgcagaggaagcgaagctgcgcgtcggccgtttccatctgcggtgcgcccggtcgcgtgccggcatggatgcgcgcgccatcgcggtaggcgagcagcgcctgcctgaagctgcgggcattcccgatcagaaatgagcgccagtcgtcgtcggctctcggcaccgaatgcgtatgattctccgccagcatggcttcggccagtgcgtcgagcagcgcccgcttgttcctgaagtgccagtaaagcgccggctgctgaacccccaaccgttccgccagtttgcgtgtcgtcagaccgtctacgccgacctcgttcaacaggtccagggcggcacggatcactgtattcggctgcaactttgtcatgattgacactttatcactgataaacataatatgtccaccaacttatcagtgataaagaatccgcgcgttcaatcggaccagcggaggctggtccggaggccagacgtgaaacccaacatacccctgatcgtaattctgagcactgtcgcgctcgacgctgtcggcatcggcctgattatgccggtgctgccgggcctcctgcgcgatctggttcactcgaacgacgtcaccgcccactatggcattctgctggcgctgtatgcgttggtgcaatttgcctgcgcacctgtgctgggcgcgctgtcggatcgtttcgggcggcggccaatcttgctcgtctcgctggccggcgccactgtcgactacgccatcatggcgacagcgcctttcctttgggttctctatatcgggcggatcgtggccggcatcaccggggcgactggggcggtagccggcgcttatattgccgatatcactgatggcgatgagcgcgcgcggcacttcggcttcatgagcgcctgtttcgggttcgggatggtcgcgggacctgtgctcggtgggctgatgggcggtttctccccccacgctccgttcttcgccgcggcagccttgaacggcctcaatttcctgacgggctgtttccttttgccggagtcgcacaaaggcgaacgccggccgttacgccgggaggctctcaacccgctcgcttcgttccggtgggcccggggcatgaccgtcgtcgccgccctgatggcggtcttcttcatcatgcaacttgtcggacaggtgccggccgcgctttgggtcattttcggcgaggatcgctttcactgggacgcgaccacgatcggcatttcgcttgccgcatttggcattctgcattcactcgcccaggcaatgatcaccggccctgtagccgcccggctcggcgaaaggcgggcactcatgctcggaatgattgccgacggcacaggctacatcctgcttgccttcgcgacacggggatggatggcgttcccgatcatggtcctgcttgcttcgggtggcatcggaatgccggcgctgcaagcaatgttgtccaggcaggtggatgaggaacgtcaggggcagctgcaaggctcactggcggcgctcaccagcctgacctcgatcgtcggacccctcctcttcacggcgatctatgcggcttctataacaacgtggaacgggtgggcatggattgcaggcgctgccctctacttgctctgcctgccggcgctgcgtcgcgggctttggagcggcgcagggcaacgagccgatcgctgatcgtggaaacgataggcctatgccatgcgggtcaaggcgacttccggcaagctatacgcgccctagaattgtcaattttaatcctctgtttatcggcagttcgtagagcgcgccgtgcgtcccgagcgatactgagcgaagcaagtgcgtcgagcagtgcccgcttgttcctgaaatgccagtaaagcgctggctgctgaacccccagccggaactgaccccacaaggccctagcgtttgcaatgcaccaggtcatcattgacccaggcgtgttccaccaggccgctgcctcgcaactcttcgcaggcttcgccgacctgctcgcgccacttcttcacgcgggtggaatccgatccgcacatgaggcggaaggtttccagcttgagcgggtacggctcccggtgcgagctgaaatagtcgaacatccgtcgggccgtcggcgacagcttgcggtacttctcccatatgaatttcgtgtagtggtcgccagcaaacagcacgacgatttcctcgtcgatcaggacctggcaacgggacgttttcttgccacggtccaggacgcggaagcggtgcagcagcgacaccgattccaggtgcccaacgcggtcggacgtgaagcccatcgccgtcgcctgtaggcgcgacaggcattcctcggccttcgtgtaataccggccattgatcgaccagcccaggtcctggcaaagctcgtagaacgtgaaggtgatcggctcgccgataggggtgcgcttcgcgtactccaacacctgctgccacaccagttcgtcatcgtcggcccgcagctcgacgccggtgtaggtgatcttcacgtccttgttgacgtggaaaatgaccttgttttgcagcgcctcgcgcgggattttcttgttgcgcgtggtgaacagggcagagcgggccgtgtcgtttggcatcgctcgcatcgtgtccggccacggcgcaatatcgaacaaggaaagctgcatttccttgatctgctgcttcgtgtgtttcagcaacgcggcctgcttggcctcgctgacctgttttgccaggtcctcgccggcggtttttcgcttcttggtcgtcatagttcctcgcgtgtcgatggtcatcgacttcgccaaacctgccgcctcctgttcgagacgacgcgaacgctccacggcggccgatggcgcgggcagggcagggggagccagttgcacgctgtcgcgctcgatcttggccgtagcttgctggaccatcgagccgacggactggaaggtttcgcggggcgcacgcatgacggtgcggcttgcgatggtttcggcatcctcggcggaaaaccccgcgtcgatcagttcttgcctgtatgccttccggtcaaacgtccgattcattcaccctccttgcgggattgccccgactcacgccggggcaatgtgcccttattcctgatttgacccgcctggtgccttggtgtccagataatccaccttatcggcaatgaagtcggtcccgtagaccgtctggccgtccttctcgtacttggtattccgaatcttgccctgcacgaataccagctccgcgaagtcgctcttcttgatggagcgcatggggacgtgcttggcaatcacgcgcaccccccggccgttttagcggctaaaaaagtcatggctctgccctcgggcggaccacgcccatcatgaccttgccaagctcgtcctgcttctcttcgatcttcgccagcagggcgaggatcgtggcatcaccgaaccgcgccgtgcgcgggtcgtcggtgagccagagtttcagcaggccgcccaggcggcccaggtcgccattgatgcgggccagctcgcggacgtgctcatagtccacgacgcccgtgattttgtagccctggccgacggccagcaggtaggcctacaggctcatgccggccgccgccgccttttcctcaatcgctcttcgttcgtctggaaggcagtacaccttgataggtgggctgcccttcctggttggcttggtttcatcagccatccgcttgccctcatctgttacgccggcggtagccggccagcctcgcagagcaggattcccgttgagcaccgccaggtgcgaataagggacagtgaagaaggaacacccgctcgcgggtgggcctacttcacctatcctgcccggctgacgccgttggatacaccaaggaaagtctacacgaaccctttggcaaaatcctgtatatcgtgcgaaaaaggatggatataccgaaaaaatcgctataatgaccccgaagcagggttatgcagcggaaaagatccgtc-3´

**pCM80-3028-3027-3029, the sequences in red encode the gene META1_3028, the sequences in blue encode the gene META1_3027, and the sequences in green encode the gene META1_3029**

5´-

gaccctttccgacgctcaccgggctggttgccctcgccgctgggctggcggccgtctatggccctgcaaacgcgccagaaacgccgtcgaagccgtgtgcgagacaccgcggccgccggcgttgtggatacctcgcggaaaacttggccctcactgacagatgaggggcggacgttgacacttgaggggccgactcacccggcgcggcgttgacagatgaggggcaggctcgatttcggccggcgacgtggagctggccagcctcgcaaatcggcgaaaacgcctgattttacgcgagtttcccacagatgatgtggacaagcctggggataagtgccctgcggtattgacacttgaggggcgcgactactgacagatgaggggcgcgatccttgacacttgaggggcagagtgctgacagatgaggggcgcacctattgacatttgaggggctgtccacaggcagaaaatccagcatttgcaagggtttccgcccgtttttcggccaccgctaacctgtcttttaacctgcttttaaaccaatatttataaaccttgtttttaaccagggctgcgccctgtgcgcgtgaccgcgcacgccgaaggggggtgcccccccttctcgaaccctcccggcccgctaacgcgggcctcccatccccccaggggctgcgcccctcggccgcgaacggcctcaccccaaaaatggcagccaagctgaccacttctgcgctcggcccttccggctggctggtttattgctgataaatctggagccggtgagcgtgggtctcgcggtatcattgcagcactggggccagatggtaagccctcccgtatcgtagttatctacacgacggggagtcaggcaactatggatgaacgaaatagacagatcgctgagataggtgcctcactgattaagcattggtaactgtcagaccaagtttactcatatatactttagattgatttaaaacttcatttttaatttaaaaggatctaggtgaagatcctttttgataatctcatgaccaaaatcccttaacgtgagttttcgttccactgagcgtcagaccccgtagaaaagatcaaaggatcttcttgagatcctttttttctgcgcgtaatctgctgcttgcaaacaaaaaaaccaccgctaccagcggtggtttgtttgccggatcaagagctaccaactctttttccgaaggtaactggcttcagcagagcgcagataccaaatactgtccttctagtgtagccgtagttaggccaccacttcaagaactctgtagcaccgcctacatacctcgctctgctaatcctgttaccagtggctgctgccagtggcgataagtcgtgtcttaccgggttggactcaagacgatagttaccggataaggcgcagcggtcgggctgaacggggggttcgtgcacacagcccagcttggagcgaacgacctacaccgaactgagatacctacagcgtgagctatgagaaagcgccacgcttcccgaagggagaaaggcggacaggtatccggtaagcggcagggtcggaacaggagagcgcacgagggagcttccagggggaaacgcctggtatctttatagtcctgtcgggtttcgccacctctgacttgagcgtcgatttttgtgatgctcgtcaggggggcggagcctatggaaaaacgccagcaacgcggcctttttacggttcctggccttttgctggccttttgctcacatgttctttcctgcgttatcccctgattctgtggataaccgtattaccgcctttgagtgagctgataccgctcgccgcagccgaacgaccgagcgcagcgagtcagtgagcgaggaagcggaagagcgcccaatacgcaaaccgcctctccccgcgcgttggccgattcattaatgcagctggcacgacaggtttcccgactggaaagcgggcagtgagcgcaacgcaattaatgtgagttagctcactcattaggcaccccaggctttacactttatgcttccggctcgtatgttgtgtggaattgtgagcggataacaatttcacacaggaaacagctatgaccatgattacgccaagctagcttcccgcttggtcgggccgcttcgcgagggcccgttgacgacaacggtgcgatgggtcccggccccggtcaagacgatgccaatacgttgcgacactacgccttggcacttttagaattgccttatcgtcctgataagaaatgtccgaccagctaaagacatcgcgtccaatcaaagcctagaaaatataggcgaagggacgctaataagtctttcataagaccgcgcaaatctaaaaatatccttagattcacgatgcggcacttcggatgacttccgagcgagcctggaacctcagaaaaacgtctgagagataccgcggatctcacacaggaaacagctatgaccatgattacgccaagcttGTGGACGGCATCATCCAAGGCCTGTCGAACTTCCGGGGAACGGTCTTTCCGGGTCAGCAGCAGATGTATCAGCGGCTGGTGCGCGACGGTCAGCAGCCTAAGGCGCTCATGATCGCCTGCGCCGATTCCCGCGTCTCGCCCGAGCACATCACGCAGTCCGGTCCGGGCGACCTGTTCGTATGCCGGAATGCCGGCAACATCGTCCCGCCCTTCTCGCAGCAGAACGGCGGCGTGTCCTCGGCGATCGAGTACGCGGTCGTCGCCCTCGGCGTGCTGGACATCGTGATCTGCGGTCATTCCGACTGCGGCGCCATGAAGGGGCTCATGAATCCCGAGGCGCTGGGCAACATGCCCAACGTGGCGGCGTGGCTGCGCCACAGCCATGCGGCGAAACAGATCGTCTGCGAGGCCTATCCCGGGGGCATGGACCCGAAGGAGCGCCACCGCGCCGTCGCCCTCGAGAACGTCGTGGTGCAACTCAACCATCTGCGCACCCACCCGAGCGTGGCGACGGCTCTGGCCCGCGGCAAGCTGCGTCTGCACGGCTGGTTCTTCGAAATCGAGAGCGGCCAGGTGCTCGCCTATTGCGGCGAGCGCGGCCGCTTCATCCCCATCGACGAAGCCACGGGTGTCCCCGTCGCCCAGGCTTCGGCGTCGCGGATCGCCACACCGGAATTCGCCGGCCCCCATGCTGGTCCCCATGCCAGCCCCCAAGCCGGGCCCGAACCCATCCCCCACGCTATCGCAGCGGAGTAGatttttgctATGCGCGCCTCCCTCGCCCGGCTGATGCCATCGACCATCGGCCGGGACCTTCCGGCCTCCTTCGTCGTCTTCCTCGTGGCGATGCCTCTCTGCATGGGCATCGCTATGGCCTCCGGTGTGCCCGCCGAGCGCGGGCTCATCACCGGCATCATCGGCGGTATCGTCGTCGGCTTCCTCGCCGGCTCGCCGCTTCAGGTCAGCGGCCCGGCCGCGGGCCTTGCCGTCATCGTCTTCGAGTTCGTGCGTGAGCACGGCATCGACGCGCTCGGGCCGGTCCTCGTCGCGGCGGGCGCCATCCAGCTTCTCGCCGGGGCGCTCCGGGTCGGCGGCTGGTTCAGGGCGATCTCGCCGGCGGTCGTGCACGGCATGCTCGCCGGCATCGGCATCCTGATCGTGCTGGCCCAGATCCACGTGCTGACCGACGCCCTGCCCAAGGCCAGCGGCCTCGACAACCTCGTCGCCATCCCCGCCGCGTTCTTCAACTTCGTCTCCGGTCCGGACGGCAACCGCCCCGGGGCCGTGATCGTCGGCCTTGCCACCATCGTTGCGATGATCGGCTGGGAGAAGATCCGGCCGGCTAAGCTCAAGCTGATTCCCGGTGCGCTCATGGGCGTGCTGGCCGGCACCGTCGTGGCCGTCGTCGGCAGCATGGACGTCAAGCGCGTCGAGGTGCCGGAGAACATCTTCTCCGCCGTGACCATGCCGGGAATGGGCGACTGGAGCCGCTTGGCCGAGCCGGCGATGATCCTGATGGCGGTCACCCTCGCGGTCATCGCCAGCGCGGAGAGCCTGCTCTCGGCAGCGGCGGTGGACCGGATGCATGACGGCCCGCGCACGCAGTACAACCGCGAACTCGGGGCGCAGGGCATCGGCAACATCCTCTGCGGCCTCGCGGGCGGCCTGCCGATGACCGGTGTGATTGTCCGGTCCTCGGCCAACGTCCAGGCGGGTGCGGCGACGCGCGCCTCCACCATCCTGCACGGGAGCTGGATCCTCGCCTTCCTGCTGGTCCTGCCGATGGTGCTGAAGGTCGTGCCGACCGCCTCCCTCGCCGGTATCCTCGTGGTGACGGGCTGGCGCCTCGTGAGCCCCGCCCACGCCTTCCACCTGCACGAGCGCTACGGCCTGCCCACCGCCGCGATCTGGCTCGCGACCATGGTGATGGTCGTCGCGACCGACCTGTTGACCGGTGTTCTCACGGGCCTTGCGCTCAGCCTCCTCCAGGTGATCCCGCACTACCTGCGTGGTCCGCTCAAGATCGAGGGCGGCGCGTCGCAGACGGTGCAAGGCGGAGCGCTTCAGGCGGTGCCGGAACTGCGGCTCTCGGGTTCGGCGACCTTCCTCCAACTGCCGCATCTCAATGCGGCGCTGGAGCGGACGCCGGAAGGCTCGCCGATCCGTCTCGCAGCACAGGATCTACGCCACGTCGATCACACCTGCCTGGAGATGATGCGGGAATGGGCGACGCGGCGGGCCAAGACCGGGTCGAGGGTCGAGGTCGTCGGCGGCGGCGCGAGCGGCCTGCATCACAGCCTCGCGATGGTCGCTCACGCCGCGCCGAAGGACTGAggatccccgggtaccATAACAACCGTTGGGGAGGCATCCCATGTTCGATATGAAGCGGATTGTGACACTCGGTCTCCTGGCGGCTGCGGCCGTTGCTTTGCCGCTCACGGCGCAGGCGCAGGACGGTGGAAACGGCAGCCGCCAGCAGGCCGGCGGCGGTTTCATGAGCAGCTACGTGGACGATCCCTACCACGACCCCCGTTCGCTCCACTCGCAGCGTGCGGGCTCGGGCCAGATCCTCGGCGCGCCGATGCTGAGCGAGAACGCCGGCGCCAAGGGTGGCGACATTCGCCCCGCCGCCCGTCTCGGCGCCGTGCAGACGCATTGGTCCGCCGGCACCGCGCCGCGCCGCGCGCGCTAAgagctcgaattcactggccgtcgttttacaacgtcgtgactgggaaaaccctggcgttacccaacttaatcgccttgcagcacatccccctttcgccagctggcgtaatagcgaagaggcccgcaccgatcgcccttcccaacagttgcgcagcctgaatggcgaatggcgcctgatgcggtattttctccttacgcatctgtgcggtatttcacaccgcatatggtgcactctcagtacaatctgctctgatgccgcatagttaagccagccccgacacccgccaacacccgctgacgcgccctgacgggcttgtctgctcccggcatccgcttacagacaagctgtgaccgtctccgggagctgcatgtgtcagaggttttcaccgtcatcaccgaaacgcgcgagacgaaagggcctcgtgatacgcctatttttataggttaatgtcatgataataatggtttcttagcaccctttctcggtccttcaacgttcctgacaacgagcctccttttcgccaatccatcgacaatcaccgcgagtccctgctcgaacgctgcgtccggaccggcttcgtcgaaggcgtctatcgcggcccgcaacagcggcgagagcggagcctgttcaacggtgccgccgcgctcgccggcatcgctgtcgccggcctgctcctcaagcacggccccaacagtgaagtagctgattgtcatcagcgcattgacggcgtccccggccgaaaaacccgcctcgcagaggaagcgaagctgcgcgtcggccgtttccatctgcggtgcgcccggtcgcgtgccggcatggatgcgcgcgccatcgcggtaggcgagcagcgcctgcctgaagctgcgggcattcccgatcagaaatgagcgccagtcgtcgtcggctctcggcaccgaatgcgtatgattctccgccagcatggcttcggccagtgcgtcgagcagcgcccgcttgttcctgaagtgccagtaaagcgccggctgctgaacccccaaccgttccgccagtttgcgtgtcgtcagaccgtctacgccgacctcgttcaacaggtccagggcggcacggatcactgtattcggctgcaactttgtcatgattgacactttatcactgataaacataatatgtccaccaacttatcagtgataaagaatccgcgcgttcaatcggaccagcggaggctggtccggaggccagacgtgaaacccaacatacccctgatcgtaattctgagcactgtcgcgctcgacgctgtcggcatcggcctgattatgccggtgctgccgggcctcctgcgcgatctggttcactcgaacgacgtcaccgcccactatggcattctgctggcgctgtatgcgttggtgcaatttgcctgcgcacctgtgctgggcgcgctgtcggatcgtttcgggcggcggccaatcttgctcgtctcgctggccggcgccactgtcgactacgccatcatggcgacagcgcctttcctttgggttctctatatcgggcggatcgtggccggcatcaccggggcgactggggcggtagccggcgcttatattgccgatatcactgatggcgatgagcgcgcgcggcacttcggcttcatgagcgcctgtttcgggttcgggatggtcgcgggacctgtgctcggtgggctgatgggcggtttctccccccacgctccgttcttcgccgcggcagccttgaacggcctcaatttcctgacgggctgtttccttttgccggagtcgcacaaaggcgaacgccggccgttacgccgggaggctctcaacccgctcgcttcgttccggtgggcccggggcatgaccgtcgtcgccgccctgatggcggtcttcttcatcatgcaacttgtcggacaggtgccggccgcgctttgggtcattttcggcgaggatcgctttcactgggacgcgaccacgatcggcatttcgcttgccgcatttggcattctgcattcactcgcccaggcaatgatcaccggccctgtagccgcccggctcggcgaaaggcgggcactcatgctcggaatgattgccgacggcacaggctacatcctgcttgccttcgcgacacggggatggatggcgttcccgatcatggtcctgcttgcttcgggtggcatcggaatgccggcgctgcaagcaatgttgtccaggcaggtggatgaggaacgtcaggggcagctgcaaggctcactggcggcgctcaccagcctgacctcgatcgtcggacccctcctcttcacggcgatctatgcggcttctataacaacgtggaacgggtgggcatggattgcaggcgctgccctctacttgctctgcctgccggcgctgcgtcgcgggctttggagcggcgcagggcaacgagccgatcgctgatcgtggaaacgataggcctatgccatgcgggtcaaggcgacttccggcaagctatacgcgccctagaattgtcaattttaatcctctgtttatcggcagttcgtagagcgcgccgtgcgtcccgagcgatactgagcgaagcaagtgcgtcgagcagtgcccgcttgttcctgaaatgccagtaaagcgctggctgctgaacccccagccggaactgaccccacaaggccctagcgtttgcaatgcaccaggtcatcattgacccaggcgtgttccaccaggccgctgcctcgcaactcttcgcaggcttcgccgacctgctcgcgccacttcttcacgcgggtggaatccgatccgcacatgaggcggaaggtttccagcttgagcgggtacggctcccggtgcgagctgaaatagtcgaacatccgtcgggccgtcggcgacagcttgcggtacttctcccatatgaatttcgtgtagtggtcgccagcaaacagcacgacgatttcctcgtcgatcaggacctggcaacgggacgttttcttgccacggtccaggacgcggaagcggtgcagcagcgacaccgattccaggtgcccaacgcggtcggacgtgaagcccatcgccgtcgcctgtaggcgcgacaggcattcctcggccttcgtgtaataccggccattgatcgaccagcccaggtcctggcaaagctcgtagaacgtgaaggtgatcggctcgccgataggggtgcgcttcgcgtactccaacacctgctgccacaccagttcgtcatcgtcggcccgcagctcgacgccggtgtaggtgatcttcacgtccttgttgacgtggaaaatgaccttgttttgcagcgcctcgcgcgggattttcttgttgcgcgtggtgaacagggcagagcgggccgtgtcgtttggcatcgctcgcatcgtgtccggccacggcgcaatatcgaacaaggaaagctgcatttccttgatctgctgcttcgtgtgtttcagcaacgcggcctgcttggcctcgctgacctgttttgccaggtcctcgccggcggtttttcgcttcttggtcgtcatagttcctcgcgtgtcgatggtcatcgacttcgccaaacctgccgcctcctgttcgagacgacgcgaacgctccacggcggccgatggcgcgggcagggcagggggagccagttgcacgctgtcgcgctcgatcttggccgtagcttgctggaccatcgagccgacggactggaaggtttcgcggggcgcacgcatgacggtgcggcttgcgatggtttcggcatcctcggcggaaaaccccgcgtcgatcagttcttgcctgtatgccttccggtcaaacgtccgattcattcaccctccttgcgggattgccccgactcacgccggggcaatgtgcccttattcctgatttgacccgcctggtgccttggtgtccagataatccaccttatcggcaatgaagtcggtcccgtagaccgtctggccgtccttctcgtacttggtattccgaatcttgccctgcacgaataccagctccgcgaagtcgctcttcttgatggagcgcatggggacgtgcttggcaatcacgcgcaccccccggccgttttagcggctaaaaaagtcatggctctgccctcgggcggaccacgcccatcatgaccttgccaagctcgtcctgcttctcttcgatcttcgccagcagggcgaggatcgtggcatcaccgaaccgcgccgtgcgcgggtcgtcggtgagccagagtttcagcaggccgcccaggcggcccaggtcgccattgatgcgggccagctcgcggacgtgctcatagtccacgacgcccgtgattttgtagccctggccgacggccagcaggtaggcctacaggctcatgccggccgccgccgccttttcctcaatcgctcttcgttcgtctggaaggcagtacaccttgataggtgggctgcccttcctggttggcttggtttcatcagccatccgcttgccctcatctgttacgccggcggtagccggccagcctcgcagagcaggattcccgttgagcaccgccaggtgcgaataagggacagtgaagaaggaacacccgctcgcgggtgggcctacttcacctatcctgcccggctgacgccgttggatacaccaaggaaagtctacacgaaccctttggcaaaatcctgtatatcgtgcgaaaaaggatggatataccgaaaaaatcgctataatgaccccgaagcagggttatgcagcggaaaagatccgtc-3´

**pCM433-MxaF*** **the sequences in red are homologous sequences locate in the upstream, and the sequences in blue are homologous sequences locate in the downstream**

ttaccaatgcttaatcagtgaggcacctatctcagcgatctgtctatttcgttcatccatagttgcctgactccccgtcgtgtagataactacgatacgggagggcttaccatctggccccagtgctgcaatgataccgcgagacccacgctcaccggctccagatttatcagcaataaaccagccagccggaagggccgagcgcagaagtggtcctgcaactttatccgcctccatccagtctattaattgttgccgggaagctagagtaagtagttcgccagttaatagtttgcgcaacgttgttgccattgctacaggcatcgtggtgtcacgctcgtcgtttggtatggcttcattcagctccggttcccaacgatcaaggcgagttacatgatcccccatgttgtgcaaaaaagcggttagctccttcggtcctccgatcgttgtcagaagtaagttggccgcagtgttatcactcatggttatggcagcactgcataattctcttactgtcatgccatccgtaagatgcttttctgtgactggtgagtactcaaccaagtcattctgagaatagtgtatgcggcgaccgagttgctcttgcccggcgtcaatacgggataataccgcgccacatagcagaactttaaaagtgctcatcattggaaaacgttcttcggggcgaaaactctcaaggatcttaccgctgttgagatccagttcgatgtaacccactcgtgcacccaactgatcttcagcatcttttactttcaccagcgtttctgggtgagcaaaaacaggaaggcaaaatgccgcaaaaaagggaataagggcgacacggaaatgttgaatactcatactcttcctttttcaatattattgaagcatttatcagggttattgtctcatgagcggatacatatttgaatgtatttagaaaaataaacaaataggggttccgcgcacatttccccgaaaagtgccacctgacgtctagatctGATCGTCATCGTCCACTTGTTGTCGCCCGGACGCATGGTCTCGTTCCACGGCGCCGGGTTGCCGGTGCCGAAGTAGATCAGGTTCGTGCCCGGATCGTAGGCGTACCAGCCCCAGTTGGTGCCGCCGCCGATCTTCCAGGCATCGCCCTCCCAGGTGCCGGTGCCGAGGCCCTTCTGACCGTAATGGGGGTTCTTGATGTTGAAGTCGGAGGCCAGCAGCAGGTCCTTGTCCGGACCCGTGGCGTAGGCGCGCCACACCTGCTCGCCGGTCTTCACGTCGTAGGCGGTCAGGTAGCCGCGCACGCCGAGCTCGGCGCCCGAGGAGCCGATGATGACCTTGTCCTTCACGACGTAGGGGGCGATCGTGAGCGTCGAGCCGACCTTGATGTCGGAGTTCTCGACCTTCCACACCGTCTCGCCGGTCTCGGCGTTGAGGGCGGCCACGTTGCCGTCGAGCTGGGTCTTGAGGATCAGCGCGGGGGTCTTGCCGTCGCCGGGCCAGTAGGCGAGGCCGCGGTTGACGAGGTCACAGCAGGCGACGGCGCGGGCGGCCGGATTCTGCTTCGGCTTGTCCTGCCACAGGATCGTGCCCGGATCGTCAAGGCCGAGAGCGAAGGTGTTGTTCGGGAACGAGGTGTGGATGTACATCTTGCCGTCGACGACGAGCGGCGCACCCTCGTGGCCGTTCAGCAGGCCGGTCGAGAACGTCCAAGCCGGCCGAAGCTGCTTCACGTTGCCCTTGTTGATCTGCTTCAGATCGCTGAAGTTGTTCGAATCGTAGTTCTTTCCGGGCATCACCCAGTTGTCGTCGCTCTTCGACAGCTCGACCAGCTTATCGTTGGCGTAGGCCCCGCTCGACAGCGCGGCCGGCGCGAGCGCCAGCATCGCCAAGGCCGAGACTGATGTCACAAACCTGCTCATCCTGCGTCTCCTCGCTGAACCGCCTCGCCTTTCGGCCTCGCGGTATCTCTCAGACGTTTTTCTGAGGTTCCAGGCTCGCTCGGAAGTCATCCGAAGTGCCGCATCGTGAATCTAAGGATATTCTTAGATTTGCGCGGTCTTATGAAAGACTTATTAGCGTCCCTTCGCCTATATTTTCTAGGCTTTGATTGGACGCGATGTCTTTAGCTGGTCGGACATTTCTTATCAGGACGATAAGGCAATTCTAAAAGTGCCAAGGCGTAGTGTCGCAACGTATTGGCATCGTCTTGACCGGGGCCGGGACCCATCGCACCGTTGTCGTCAACGGGCCCTCGCGAAGCGGCCCGACCAAGCGGGAGGCTTGTCGGCCATGGCGCAGGGGCAGCGCGCGCAGGGGAGGCTGGCGGTGTGCGGGCTCCTCGCCCTCGTGGTTTGGGCCGGGGGCGGCACCGCACTTGGAGACGATGCGCCGCACCCGCCGCCCGATGCCGCGGCGCCGGCCCGAGAGCGCGCACGCGCCATCACCGAAACGGCGGCGCTCTATCCCCAGGACGGGCGCCGCGACCTGACGAGCAGCGACCGCGTCCTCTATCCGGGGGCGGGCGTCCTCTGGTGCCGCCGTTCGGACGGCGTGCCGCAAAAGGCGGCCGCCGCGTGGCTGATCGGCACGCCGTCGCTCGTGATGCTGAACGCGCACAACTTCCGCAACCGGCAGCTCGAAACGACGCGGGCCGTCGGCGACTGCTACTTCCAGATCGGCGGCCGCAACTACGATTTCGTCGCCGAGAGCCTGCATCTCGGCGTCGCCGCGGACGCGACGCGCCTCCACATCACCGACGATTGGGCGCTGCTGCGCCTCGCGCGACCGGCCGAGGCGCTGCGCCAGCCGGTGCCCGATGCGCCCCCCGACCTCTCCACCGGCGACCTGTCGCTCGCCGTGACCCTGGTCGCCCCCGGCGGTCACCAGAACTACCGGGGCGACAGCAGCCTGGAGACCTGCACCGTGCGCCGGATCGACCCGCCGACGGAAGGCGGCATCCGGCGGGCCCGCCACGATTGCAACGACGGCTACGGCGGCTCCGGTTCGGGACTGTTCGACGAGGgagctcactagaggatccagccgaccaggctttccacgcccgcgtgccgctccatgtcgttcgcgcggttctcggaaacgcgctgccgcgtttcgtgattgtcacgctcaagcccgtagtcccgttcgagcgtcgcgcagaggtcagcgagggcgcggtaggcccgatacggctcatggatggtgtttcgggtcgggtgaatcttgttgatggcgatatggatgtgcaggttgtcggtgtcgtgatgcacggcactgacgcgctgatgctcggcgaagccaagcccagcgcagatgcggtcctcaatcgcgcgcaacgtctccgcgtcgggcttctctcccgcgcggaagctaaccagcaggtgataggtcttgtcggcctcggaacgggtgttgccgtgctgggtcgccatcacctcggccatgacagcgggcagggtgtttgcctcgcagttcgtgacgcgcacgtgacccaggcgctcggtcttgccttgctcgtcggtgatgtacttcaccagctccgcgaagtcgctcttcttgatggagcgcatggggacgtgcttggcaatcacgcgcaccccccggccgttttagcggctaaaaaagtcatggctctgccctcgggcggaccacgcccatcatgaccttgccaagctcgtcctgcttctcttcgatcttcgccagcagggcgaggatcgtggcatcaccgaaccgcgccgtgcgcgggtcgtcggtgagccagagtttcagcaggccgcccaggcggcccaggtcgccattgatgcgggccagctcgcggacgtgctcatagtccacgacgcccgtgattttgtagccctggccgacggccagcaggtaggccgacaggctcatgccggccgccgccgccttttcctcaatcgctcttcgttcgtctggaaggcagtacaccttgataggtgggctgcccttcctggttggcttggtttcatcagccatccgcttgccctcatctgttacgccggcggtagccggccagcctcgcagagcaggattcccgttgagcaccgccaggtgcgaataagggacagtgaagaaggaacacccgctcgcgggtgggcctacttcacctatcctgcccggctgacgccgttggatacaccaaggaaagtctacacgaaccctttggcaaaatcctgtatatcgtgcgaaaaaggatggatataccgaaaaaatcgctataatgaccccgaagcagggttatgcagcggaaaagcgctgcttccctgctgttttgtggaatatctaccgactggaaacaggcaaatgcaggaaattactgaactgaggggacaggcgagagacgatgccaaagagctacaccgacgagctggccgagtgggttgaatcccgcgcggccaagaagcgccggcgtgatgaggctgcggttgcgttcctggcggtgagggcggatgtcgaggcggcgttagcgtccggctatgcgctcgtcaccatttgggagcacatgcgggaaacggggaaggtcaagttctcctacgagacgttccgctcgcacgccaggcggcacatcaaggccaagcccgccgatgtgcccgcaccgcaggccaaggctgcggaacccgcgccggcacccaagacgccggagccacggcggccgaagcaggggggcaaggctgaaaagccggcccccgctgcggccccgaccggcttcaccttcaacccaacaccggacaaaaaggatccccaattctcatgtttgacagcttatcatcgataagctttaatgcggtagtttatcacagttaaattgctaacgcagtcaggcaccgtgtatgaaatctaacaatgcgctcatcgtcatcctcggcaccgtcaccctggatgctgtaggcataggcttggttatgccggtactgccgggcctcttgcgggatatcgtccattccgacagcatcgccagtcactatggcgtgctgctagcgctatatgcgttgatgcaatttctatgcgcacccgttctcggagcactgtccgaccgctttggccgccgcccagtcctgctcgcttcgctacttggagccactatcgactacgcgatcatggcgaccacacccgtcctgtggatcctctacgccggacgcatcgtggccggcatcaccggcgccacaggtgcggttgctggcgcctatatcgccgacatcaccgatggggaagatcgggctcgccacttcgggctcatgagcgcttgtttcggcgtgggtatggtggcaggccccgtggccgggggactgttgggcgccatctccttgcatgcaccattccttgcggcggcggtgctcaacggcctcaacctactactgggctgcttcctaatgcaggagtcgcataagggagagcgtcgaccgatgcccttgagagccttcaacccagtcagctccttccggtgggcgcggggcatgactatcgtcgccgcacttatgactgtcttctttatcatgcaactcgtaggacaggtgccggcagcgctctgggtcattttcggcgaggaccgctttcgctggagcgcgacgatgatcggcctgtcgcttgcggtattcggaatcttgcacgccctcgctcaagccttcgtcactggtcccgccaccaaacgtttcggcgagaagcaggccattatcgccggcatggcggccgacgcgctgggctacgtcttgctggcgttcgcgacgcgaggctggatggccttccccattatgattcttctcgcttccggcggcatcgggatgcccgcgttgcaggccatgctgtccaggcaggtagatgacgaccatcagggacagcttcaaggatcgctcgcggctcttaccagcctaacttcgatcactggaccgctgatcgtcacggcgatttatgccgcctcggcgagcacatggaacgggttggcatggattgtaggcgccgccctataccttgtctgcctccccgcgttgcgtcgcggtgcatggagccgggccacctcgacctgaatggaagccggcggcacctcgctaacggattcaccactccaagaattggagccaatcaattcttgcggagaactgtgaatgcgcaaaccaacccttggcagaacatatccatcgcgtccgccatctccagcagccgcacgcggcgcatctcgggcagcgttgggtcctggccgggaattacgccccgccctgccactcatcgcagtactgttgtaattcattaagcattctgccgacatggaagccatcacaaacggcatgatgaacctgaatcgccagcggcatcagcaccttgtcgccttgcgtataatatttgcccatggtgaaaacgggggcgaagaagttgtccatattggccacgtttaaatcaaaactggtgaaactcacccagggattggctgagacgaaaaacatattctcaataaaccctttagggaaataggccaggttttcaccgtaacacgccacatcttgcgaatatatgtgtagaaactgccggaaatcgtcgtggtattcactccagagcgatgaaaacgtttcagtttgctcatggaaaacggtgtaacaagggtgaacactatcccatatcaccagctcaccgtctttcattgccatacggaattccggatgagcattcatcaggcgggcaagaatgtgaataaaggccggataaaacttgtgcttatttttctttacggtctttaaaaaggccgtaatatccagctgaacggtctggttataggtacattgagcaactgactgaaatgcctcaaaatgttctttacgatgccattgggatatatcaacggtggtatatccagtgatttttttctccattttagcttccttagctcctgccctatgggattcacctttatgttgataagaaataaaagaaaatgccaataggatatcggcattttcttttgcgtttttatttgttaactgttaattgtccttgttcaaggatgctgtctttgacaacagatgttttcttgcctttgatgttcagcaggaagcttggcgcaaacgttgattgtttgtctgcgtagaatcctctgtttgtcatatagcttgtaatcacgacattgtttcctttcgcttgaggtacagcgaagtgtgagtaagtaaaggttacatcgttaggatcaagatccatttttaacacaaggccagttttgttcagcggcttgtatgggccagttaaagaattagaaacataaccaagcatgtaaatatcgttagacgtaatgccgtcaatcgtcatttttgatccgcgggagtcagtgaacaggtaccatttgccgttcattttaaagacgttcgcgcgttcaatttcatctgttactgtgttagatgcaatcagcggtttcatcacttttttcagtgtgtaatcatcgtttagctcaatcataccgagagcgccgtttgctaactcagccgtgcgttttttatcgctttgcagaagtttttgactttcttgacggaagaatgatgtgcttttgccatagtatgctttgttaaataaagattcttcgccttggtagccatcttcagttccagtgtttgcttcaaatactaagtatttgtggcctttatcttctacgtagtgaggatctctcagcgtatggttgtcgcctgagctgtagttgccttcatcgatgaactgctgtacattttgatacgtttttccgtcaccgtcaaagattgatttataatcctctacaccgttgatgttcaaagagctgtctgatgctgatacgttaacttgtgcagttgtcagtgtttgtttgccgtaatgtttaccggagaaatcagtgtagaataaacggatttttccgtcagatgtaaatgtggctgaacctgaccattcttgtgtttggtcttttaggatagaatcatttgcatcgaatttgtcgctgtctttaaagacgcggccagcgtttttccagctgtcaatagaagtttcgccgactttttgatagaacatgtaaatcgatgtgtcatccgcatttttaggatctccggctaatgcaaagacgatgtggtagccgtgatagtttgcgacagtgccgtcagcgttttgtaatggccagctgtcccaaacgtccaggccttttgcagaagagatatttttaattgtggacgaatcgaattcaggaacttgatatttttcatttttttgctgttcagggatttgcagcatatcatggcgtgtaatatgggaaatgccgtatgtttccttatatggcttttggttcgtttctttcgcaaacgcttgagttgcgcctcctgccagcagtgcggtagtaaaggttaatactgttgcttgttttgcaaactttttgatgttcatcgttcatgtctccttttttatgtactgtgttagcggtctgcttcttccagccctcctgtttgaagatggcaagttagttacgcacaataaaaaaagacctaaaatatgtaaggggtgacgccaaagtatacactttgccctttacacattttaggtcttgcctgctttatcagtaacaaacccgcgcgatttacttttcgacctcattctattagactctcgtttggattgcaactggtctattttcctcttttgtttgatagaaaatcataaaaggatttgcagactacgggcctaaagaactaaaaaatctatctgtttcttttcattctctgtattttttatagtttctgttgcatgggcataaagttgcctttttaatcacaattcagaaaatatcataatatctcatttcactaaataatagtgaacggcaggtatatgtgatgggttaaaaaggatcgatcctctagccacgggtgcgcatgatcgtgctcctgtcgttgaggacccggctaggctggcggggttgccttactggttagcagaatgaatcaccgatacgcgagcgaacgtgaagcgactgctgctgcaaaacgtctgcgacctgagcaacaacatgaatggtcttcggtttccgtgtttcgtaaagtctggaaacgcggaagtcagcgctcttccgcttcctcgctcactgactcgctgcgctcggtcgttcggctgcggcgagcggtatcagctcactcaaaggcggtaatacggttatccacagaatcaggggataacgcaggaaagaacatgtgagcaaaaggccagcaaaaggccaggaaccgtaaaaaggccgcgttgctggcgtttttccataggctccgcccccctgacgagcatcacaaaaatcgacgctcaagtcagaggtggcgaaacccgacaggactataaagataccaggcgtttccccctggaagctccctcgtgcgctctcctgttccgaccctgccgcttaccggatacctgtccgcctttctcccttcgggaagcgtggcgctttctcatagctcacgctgtaggtatctcagttcggtgtaggtcgttcgctccaagctgggctgtgtgcacgaaccccccgttcagcccgaccgctgcgccttatccggtaactatcgtcttgagtccaacccggtaagacacgacttatcgccactggcagcagccactggtaacaggattagcagagcgaggtatgtaggcggtgctacagagttcttgaagtggtggcctaactacggctacactagaaggacagtatttggtatctgcgctctgctgaagccagttaccttcggaaaaagagttggtagctcttgatccggcaaacaaaccaccgctggtagcggtggtttttttgtttgcaagcagcagattacgcgcagaaaaaaaggatctcaagaagatcctttgatcttttctacggggtctgacgctcagtggaacgaaaactcacgttaagggattttggtcatgagattatcaaaaaggatcttcacctagatccttttaaattaaaaatgaagttttaaatcaatctaaagtatatatgagtaaacttggtctgacag

**pCM433-*P_mxaF_-P_3028-3027_*, the sequences in red are homologous sequences locate in the upstream of the native promoter of the operon META1_3028-META1_3027, and the sequences in blue are *P_mxaF_* promoter, and the sequences in purple are homologous sequences locate in the downstream of the native promoter of the operon META1_3028-META1_3027.**

ttaccaatgcttaatcagtgaggcacctatctcagcgatctgtctatttcgttcatccatagttgcctgactccccgtcgtgtagataactacgatacgggagggcttaccatctggccccagtgctgcaatgataccgcgagacccacgctcaccggctccagatttatcagcaataaaccagccagccggaagggccgagcgcagaagtggtcctgcaactttatccgcctccatccagtctattaattgttgccgggaagctagagtaagtagttcgccagttaatagtttgcgcaacgttgttgccattgctacaggcatcgtggtgtcacgctcgtcgtttggtatggcttcattcagctccggttcccaacgatcaaggcgagttacatgatcccccatgttgtgcaaaaaagcggttagctccttcggtcctccgatcgttgtcagaagtaagttggccgcagtgttatcactcatggttatggcagcactgcataattctcttactgtcatgccatccgtaagatgcttttctgtgactggtgagtactcaaccaagtcattctgagaatagtgtatgcggcgaccgagttgctcttgcccggcgtcaatacgggataataccgcgccacatagcagaactttaaaagtgctcatcattggaaaacgttcttcggggcgaaaactctcaaggatcttaccgctgttgagatccagttcgatgtaacccactcgtgcacccaactgatcttcagcatcttttactttcaccagcgtttctgggtgagcaaaaacaggaaggcaaaatgccgcaaaaaagggaataagggcgacacggaaatgttgaatactcatactcttcctttttcaatattattgaagcatttatcagggttattgtctcatgagcggatacatatttgaatgtatttagaaaaataaacaaataggggttccgcgcacatttccccgaaaagtgccacctgacgtctagatcttcggtcagcacgtggatctgggccagCACGATCAGGATGCCGATGCCGGCGAGCATGCCGTGCACGACCGCCGGCGAGATCGCCCTGAACCAGCCGCCGACCCGGAGCGCCCCGGCGAGAAGCTGGATGGCGCCCGCCGCGACGAGGACCGGCCCGAGCGCGTCGATGCCGTGCTCACGCACGAACTCGAAGACGATGACGGCAAGGCCCGCGGCCGGGCCGCTGACCTGAAGCGGCGAGCCGGCGAGGAAGCCGACGACGATACCGCCGATGATGCCGGTGATGAGCCCGCGCTCGGCGGGCACACCGGAGGCCATAGCGATGCCCATGCAGAGAGGCATCGCCACGAGGAAGACGACGAAGGAGGCCGGAAGGTCCCGGCCGATGGTCGATGGCATCAGCCGGGCGAGGGAGGCGCGCATAGCAAAAATCTACTCCGCTGCGATAGCGTGGGGGATGGGTTCGGGCCCGGCTTGGGGGCTGGCATGGGGACCAGCATGGGGGCCGGCGAATTCCGGTGTGGCGATCCGCGACGCCGAAGCCTGGGCGACGGGGACACCCGTGGCTTCGTCGATGGGGATGAAGCGGCCGCGCTCGCCGCAATAGGCGAGCACCTGGCCGCTCTCGATTTCGAAGAACCAGCCGTGCAGACGCAGCTTGCCGCGGGCCAGAGCCGTCGCCACGCTCGGGTGGGTGCGCAGATGGTTGAGTTGCACCACGACGTTCTCGAGGGCGACGGCGCGGTGGCGCTCCTTCGGGTCCATGCCCCCGGGATAGGCCTCGCAGACGATCTGTTTCGCCGCATGGCTGTGGCGCAGCCACGCCGCCACGTTGGGCATGTTGCCCAGCGCCTCGGGATTCATGAGCCCCTTCATGGCGCCGCAGTCGGAATGACCGCAGATCACGATGTCCAGCACGCCGAGGGCGACGACCGCGTACTCGATCGCCGAGGACACGCCGCCGTTCTGCTGCGAGAAGGGCGGGACGATGTTGCCGGCATTCCGGCATACGAACAGGTCGCCCGGACCGGACTGCGTGATGTGCTCGGGCGAGACGCGGGAATCGGCGCAGGCGATCATGAGCGCCTTAGGCTGCTGACCGTCGCGCACCAGCCGCTGATACATCTGCTGCTGACCCGGAAAGACCGTTCCCCGGAAGTTCGACAGGCCTTGGATGATGCCGTCCACGCGGTATCTCTCAGACGTTTTTCTGAGGTTCCAGGCTCGCTCGGAAGTCATCCGAAGTGCCGCATCGTGAATCTAAGGATATTTTTAGATTTGCGCGGTCTTATGAAAGACTTATTAGCGTCCCTTCGCCTATATTTTCTAGGCTTTGATTGGACGCGATGTCTTTAGCTGGTCGGACATTTCTTATCAGGACGATAAGGCAATTCTAAAAGTGCCAAGGCGTAGTGTCGCAACGTATTGGCATCGTCTTGACCGGGGCCGGGACCCATCGCACCGTTGTCGTCAACGGGCCCTCGCGAAGCGGCCCGACCAAGCGGGTTAGCGCGCGCGGCGCGGCGCGGTGCCGGCGGACCAATGCGTCTGCACGGCGCCGAGACGGGCGGCGGGGCGAATGTCGCCACCCTTGGCGCCGGCGTTCTCGCTCAGCATCGGCGCGCCGAGGATCTGGCCCGAGCCCGCACGCTGCGAGTGGAGCGAACGGGGGTCGTGGTAGGGATCGTCCACGTAGCTGCTCATGAAACCGCCGCCGGCCTGCTGGCGGCTGCCGTTTCCACCGTCCTGCGCCTGCGCCGTGAGCGGCAAAGCAACGGCCGCAGCCGCCAGGAGACCGAGTGTCACAATCCGCTTCATATCGAACATCCTCTCGTAATCCCTGAGTGGCGAACTTTTTTTCGTCTTCTTCAGGTGTCGCAATAAGACAGTAATTCCGTGAATTATATTTGGAAAGTGCGTTTTATCACGACGATATACTTTTAATTTAATCCTGGCAGCTCGTTTGCTTGGTGCGCAACGGCACCTTTCGCCGATAAACTTGACGTGATTGAGGGATGAAAATTGATTGCGTTTGATGAGGATTAAACTGGACTGCACTTCGGTGAACGACCCTGATCGTTCATCGACGAGAAGAGCGGCAGCCGATCATCAAGGTCAGCGAGCCGGTGAGACGGCGTAGCGCCTGGACGAGGGCCGCGACCTCCATCGCGAACGGATCAAGCAGCCATTGAACTGCCGGATTCGAACGGCGGACTTAGAGCAACCTGCCTTCCACTGGAGCAGGGCGGTGAGCAGCAAAGAGCAGACGTGCCGGCGAGGACCATTTCCAAAGGGCGGAGGAGAATATCAGCACTCGCACTTGATCGATCGTCTGTCGATCAAAGTGTCTTGAAGCAAGAGGGGCACTTGCAAATCGGACAAATACTCGTCAATTTTTATTAAGAGTGCTTCCCCAGTCTTCATACTGGTGCCGCTTCTCGAAGCACCGCTCCCGCTCCAACGATCGTTGCGCCACAATAACTCAGTGCTTTCGGTGGTCCCCACATtgagctcactagaggatccagccgaccaggctttccacgcccgcgtgccgctccatgtcgttcgcgcggttctcggaaacgcgctgccgcgtttcgtgattgtcacgctcaagcccgtagtcccgttcgagcgtcgcgcagaggtcagcgagggcgcggtaggcccgatacggctcatggatggtgtttcgggtcgggtgaatcttgttgatggcgatatggatgtgcaggttgtcggtgtcgtgatgcacggcactgacgcgctgatgctcggcgaagccaagcccagcgcagatgcggtcctcaatcgcgcgcaacgtctccgcgtcgggcttctctcccgcgcggaagctaaccagcaggtgataggtcttgtcggcctcggaacgggtgttgccgtgctgggtcgccatcacctcggccatgacagcgggcagggtgtttgcctcgcagttcgtgacgcgcacgtgacccaggcgctcggtcttgccttgctcgtcggtgatgtacttcaccagctccgcgaagtcgctcttcttgatggagcgcatggggacgtgcttggcaatcacgcgcaccccccggccgttttagcggctaaaaaagtcatggctctgccctcgggcggaccacgcccatcatgaccttgccaagctcgtcctgcttctcttcgatcttcgccagcagggcgaggatcgtggcatcaccgaaccgcgccgtgcgcgggtcgtcggtgagccagagtttcagcaggccgcccaggcggcccaggtcgccattgatgcgggccagctcgcggacgtgctcatagtccacgacgcccgtgattttgtagccctggccgacggccagcaggtaggccgacaggctcatgccggccgccgccgccttttcctcaatcgctcttcgttcgtctggaaggcagtacaccttgataggtgggctgcccttcctggttggcttggtttcatcagccatccgcttgccctcatctgttacgccggcggtagccggccagcctcgcagagcaggattcccgttgagcaccgccaggtgcgaataagggacagtgaagaaggaacacccgctcgcgggtgggcctacttcacctatcctgcccggctgacgccgttggatacaccaaggaaagtctacacgaaccctttggcaaaatcctgtatatcgtgcgaaaaaggatggatataccgaaaaaatcgctataatgaccccgaagcagggttatgcagcggaaaagcgctgcttccctgctgttttgtggaatatctaccgactggaaacaggcaaatgcaggaaattactgaactgaggggacaggcgagagacgatgccaaagagctacaccgacgagctggccgagtgggttgaatcccgcgcggccaagaagcgccggcgtgatgaggctgcggttgcgttcctggcggtgagggcggatgtcgaggcggcgttagcgtccggctatgcgctcgtcaccatttgggagcacatgcgggaaacggggaaggtcaagttctcctacgagacgttccgctcgcacgccaggcggcacatcaaggccaagcccgccgatgtgcccgcaccgcaggccaaggctgcggaacccgcgccggcacccaagacgccggagccacggcggccgaagcaggggggcaaggctgaaaagccggcccccgctgcggccccgaccggcttcaccttcaacccaacaccggacaaaaaggatccccaattctcatgtttgacagcttatcatcgataagctttaatgcggtagtttatcacagttaaattgctaacgcagtcaggcaccgtgtatgaaatctaacaatgcgctcatcgtcatcctcggcaccgtcaccctggatgctgtaggcataggcttggttatgccggtactgccgggcctcttgcgggatatcgtccattccgacagcatcgccagtcactatggcgtgctgctagcgctatatgcgttgatgcaatttctatgcgcacccgttctcggagcactgtccgaccgctttggccgccgcccagtcctgctcgcttcgctacttggagccactatcgactacgcgatcatggcgaccacacccgtcctgtggatcctctacgccggacgcatcgtggccggcatcaccggcgccacaggtgcggttgctggcgcctatatcgccgacatcaccgatggggaagatcgggctcgccacttcgggctcatgagcgcttgtttcggcgtgggtatggtggcaggccccgtggccgggggactgttgggcgccatctccttgcatgcaccattccttgcggcggcggtgctcaacggcctcaacctactactgggctgcttcctaatgcaggagtcgcataagggagagcgtcgaccgatgcccttgagagccttcaacccagtcagctccttccggtgggcgcggggcatgactatcgtcgccgcacttatgactgtcttctttatcatgcaactcgtaggacaggtgccggcagcgctctgggtcattttcggcgaggaccgctttcgctggagcgcgacgatgatcggcctgtcgcttgcggtattcggaatcttgcacgccctcgctcaagccttcgtcactggtcccgccaccaaacgtttcggcgagaagcaggccattatcgccggcatggcggccgacgcgctgggctacgtcttgctggcgttcgcgacgcgaggctggatggccttccccattatgattcttctcgcttccggcggcatcgggatgcccgcgttgcaggccatgctgtccaggcaggtagatgacgaccatcagggacagcttcaaggatcgctcgcggctcttaccagcctaacttcgatcactggaccgctgatcgtcacggcgatttatgccgcctcggcgagcacatggaacgggttggcatggattgtaggcgccgccctataccttgtctgcctccccgcgttgcgtcgcggtgcatggagccgggccacctcgacctgaatggaagccggcggcacctcgctaacggattcaccactccaagaattggagccaatcaattcttgcggagaactgtgaatgcgcaaaccaacccttggcagaacatatccatcgcgtccgccatctccagcagccgcacgcggcgcatctcgggcagcgttgggtcctggccgggaattacgccccgccctgccactcatcgcagtactgttgtaattcattaagcattctgccgacatggaagccatcacaaacggcatgatgaacctgaatcgccagcggcatcagcaccttgtcgccttgcgtataatatttgcccatggtgaaaacgggggcgaagaagttgtccatattggccacgtttaaatcaaaactggtgaaactcacccagggattggctgagacgaaaaacatattctcaataaaccctttagggaaataggccaggttttcaccgtaacacgccacatcttgcgaatatatgtgtagaaactgccggaaatcgtcgtggtattcactccagagcgatgaaaacgtttcagtttgctcatggaaaacggtgtaacaagggtgaacactatcccatatcaccagctcaccgtctttcattgccatacggaattccggatgagcattcatcaggcgggcaagaatgtgaataaaggccggataaaacttgtgcttatttttctttacggtctttaaaaaggccgtaatatccagctgaacggtctggttataggtacattgagcaactgactgaaatgcctcaaaatgttctttacgatgccattgggatatatcaacggtggtatatccagtgatttttttctccattttagcttccttagctcctgccctatgggattcacctttatgttgataagaaataaaagaaaatgccaataggatatcggcattttcttttgcgtttttatttgttaactgttaattgtccttgttcaaggatgctgtctttgacaacagatgttttcttgcctttgatgttcagcaggaagcttggcgcaaacgttgattgtttgtctgcgtagaatcctctgtttgtcatatagcttgtaatcacgacattgtttcctttcgcttgaggtacagcgaagtgtgagtaagtaaaggttacatcgttaggatcaagatccatttttaacacaaggccagttttgttcagcggcttgtatgggccagttaaagaattagaaacataaccaagcatgtaaatatcgttagacgtaatgccgtcaatcgtcatttttgatccgcgggagtcagtgaacaggtaccatttgccgttcattttaaagacgttcgcgcgttcaatttcatctgttactgtgttagatgcaatcagcggtttcatcacttttttcagtgtgtaatcatcgtttagctcaatcataccgagagcgccgtttgctaactcagccgtgcgttttttatcgctttgcagaagtttttgactttcttgacggaagaatgatgtgcttttgccatagtatgctttgttaaataaagattcttcgccttggtagccatcttcagttccagtgtttgcttcaaatactaagtatttgtggcctttatcttctacgtagtgaggatctctcagcgtatggttgtcgcctgagctgtagttgccttcatcgatgaactgctgtacattttgatacgtttttccgtcaccgtcaaagattgatttataatcctctacaccgttgatgttcaaagagctgtctgatgctgatacgttaacttgtgcagttgtcagtgtttgtttgccgtaatgtttaccggagaaatcagtgtagaataaacggatttttccgtcagatgtaaatgtggctgaacctgaccattcttgtgtttggtcttttaggatagaatcatttgcatcgaatttgtcgctgtctttaaagacgcggccagcgtttttccagctgtcaatagaagtttcgccgactttttgatagaacatgtaaatcgatgtgtcatccgcatttttaggatctccggctaatgcaaagacgatgtggtagccgtgatagtttgcgacagtgccgtcagcgttttgtaatggccagctgtcccaaacgtccaggccttttgcagaagagatatttttaattgtggacgaatcgaattcaggaacttgatatttttcatttttttgctgttcagggatttgcagcatatcatggcgtgtaatatgggaaatgccgtatgtttccttatatggcttttggttcgtttctttcgcaaacgcttgagttgcgcctcctgccagcagtgcggtagtaaaggttaatactgttgcttgttttgcaaactttttgatgttcatcgttcatgtctccttttttatgtactgtgttagcggtctgcttcttccagccctcctgtttgaagatggcaagttagttacgcacaataaaaaaagacctaaaatatgtaaggggtgacgccaaagtatacactttgccctttacacattttaggtcttgcctgctttatcagtaacaaacccgcgcgatttacttttcgacctcattctattagactctcgtttggattgcaactggtctattttcctcttttgtttgatagaaaatcataaaaggatttgcagactacgggcctaaagaactaaaaaatctatctgtttcttttcattctctgtattttttatagtttctgttgcatgggcataaagttgcctttttaatcacaattcagaaaatatcataatatctcatttcactaaataatagtgaacggcaggtatatgtgatgggttaaaaaggatcgatcctctagccacgggtgcgcatgatcgtgctcctgtcgttgaggacccggctaggctggcggggttgccttactggttagcagaatgaatcaccgatacgcgagcgaacgtgaagcgactgctgctgcaaaacgtctgcgacctgagcaacaacatgaatggtcttcggtttccgtgtttcgtaaagtctggaaacgcggaagtcagcgctcttccgcttcctcgctcactgactcgctgcgctcggtcgttcggctgcggcgagcggtatcagctcactcaaaggcggtaatacggttatccacagaatcaggggataacgcaggaaagaacatgtgagcaaaaggccagcaaaaggccaggaaccgtaaaaaggccgcgttgctggcgtttttccataggctccgcccccctgacgagcatcacaaaaatcgacgctcaagtcagaggtggcgaaacccgacaggactataaagataccaggcgtttccccctggaagctccctcgtgcgctctcctgttccgaccctgccgcttaccggatacctgtccgcctttctcccttcgggaagcgtggcgctttctcatagctcacgctgtaggtatctcagttcggtgtaggtcgttcgctccaagctgggctgtgtgcacgaaccccccgttcagcccgaccgctgcgccttatccggtaactatcgtcttgagtccaacccggtaagacacgacttatcgccactggcagcagccactggtaacaggattagcagagcgaggtatgtaggcggtgctacagagttcttgaagtggtggcctaactacggctacactagaaggacagtatttggtatctgcgctctgctgaagccagttaccttcggaaaaagagttggtagctcttgatccggcaaacaaaccaccgctggtagcggtggtttttttgtttgcaagcagcagattacgcgcagaaaaaaaggatctcaagaagatcctttgatcttttctacggggtctgacgctcagtggaacgaaaactcacgttaagggattttggtcatgagattatcaaaaaggatcttcacctagatccttttaaattaaaaatgaagttttaaatcaatctaaagtatatatgagtaaacttggtctgacag
